# Synthesized clinical notes enable training robust multimodal AI models from unimodal dermatology datasets

**DOI:** 10.1038/s41746-026-03040-3

**Published:** 2026-07-17

**Authors:** Niccolo Marini, Zhaohui Liang, Sivaramakrishnan Rajaraman, Zhiyun Xue, Sameer Antani

**Affiliations:** National Library of Medicine, National Institutes of Health, Bethesda, MD USA

**Keywords:** Computational biology and bioinformatics, Diseases, Health care, Mathematics and computing, Medical research

## Abstract

Multimodal (MM) algorithms and foundation models have shown strong potential for automated skin lesion analysis in dermatology, however, their translation to clinical practice is hindered by the limited availability of large, high-quality image-text datasets. Most public dermatology datasets are small, unimodal, and paired with heterogeneous labels and metadata, restricting effective model development. Large Language Models (LLMs) provide an opportunity to synthesize clinical notes from existing unimodal datasets, but their application is limited by hallucinations that can hinder MM training. This paper investigates and evaluates strategies to generate reliable clinical notes using existing LLMs and metadata paired with real images, aiming to mitigate hallucinations and improve performance on downstream tasks. We train a MM architecture on real dermatology images paired with synthesized notes and evaluate it across 15 datasets (6 internal, 9 external) on cross-modal retrieval and zero-shot learning tasks. Results demonstrate improved robustness and generalization compared to state-of-the-art medical foundation models, under specific synthesis conditions.

## Introduction

The use of Artificial Intelligence (AI) and Large Language Models (LLM) to support dermatologists in the diagnosis of skin lesions is of increasing interest. However, critical challenges still limit their application and translation to clinical practice.

Skin lesions represent a major global health issue, affecting a large proportion (30–70%) of the worldwide population^1–4^ and are ranked as the fourth major cause of non-fatal disease across ages and regions^1,5,6^. While most lesions are benign, those that are malignant require accurate and time-consuming analysis to determine risk^1,2,7,8^. The clinical assessment of skin lesions is challenging^1,3,5^, even for trained dermatologists, due to the heterogeneity of lesion manifestations in terms of their appearance, i.e., color, borders, and morphological structures^9–11^, ranging from benign nevi to malignant conditions such as melanomas, squamous cell cancers, and basal cell cancers^2,5,12,13^, respective to the skin tone of the affected patient. This complexity is aggravated by the limited availability of dermatologists, which does not scale with the increasing demand for dermatological diagnosis^2,13–17^. As a result, patients often experience delays in expert evaluation and are assessed by non-dermatologist clinicians, which may provide inaccurate diagnosis^3,12,18^. This increases the risk of undiagnosed or late-diagnosed malignant lesions with potentially lethal consequences. These constraints have motivated the growing interest in AI-driven computational approaches to support medical experts^2–4,7,13,19–21^.

The recent advancements in AI, in particular Deep Learning (DL) algorithms, have demonstrated clear potential for application to dermatology, with automatic models achieving dermatologist-level performance in specific tasks^2,6,22–26^, such as lesion classification. DL algorithms are especially promising when integrating multiple medical data modalities, including images, free-text clinical notes (e.g., textual descriptions), structured patient records. Multimodal (MM) algorithms aim to learn more comprehensive medical representations, leveraging the complementary nature of different modalities, as images and clinical notes jointly convey meaningful information^27,28^ about patient health. Not only do medical representations using images and clinical notes, collectively, offer meaningful information^27,28^, but they also enable text-based query of image datasets^29–34^. The possibility of querying images, using the text as input, allows DL models to be trained more flexibly, reducing the dependency on merely categorical labels, and aid their optimization to a specific task^31^. These characteristics can explain the recent success of large-scale foundation models^28,29,32,34–41^, which are pre-trained on vast multimodal and leverage text as a flexible query interface to retrieve, interpret, or generate relevant outputs without task-specific annotations. The development of foundation models is also reshaping the dermatology domain, with multiple models such as SkinGPT4^2^, PanDerm^4^, Derm1M^3^, paving the way for an increased use of such models in the domain.

Despite recent advances, the development of MM and foundation models in dermatology still faces challenging bottlenecks that are primarily related to data availability and quality. Most publicly available dermatology datasets are unimodal, limited in size, and composed mainly of dermoscopic or clinical images^3,4,13,20,21,42–47^, often photographs, paired with heterogeneous metadata or categorical labels that are lacking expressive textual information^48^. Annotations are often inconsistent across datasets, provide limited descriptive detail, and frequently focus on a narrow subset of skin conditions, particularly malignant lesions. The few existing multimodal datasets, such as Derm1M^3^, SKINCON^49^, show additional limitations affecting both modalities. Derm1M, for example, includes million-scale image-text couples, but the images are often noisy and heterogeneous, consisting largely of photographs, textbook figures, or internet images in which lesions are not centered and may appear as background elements. Furthermore, the images are paired with high-level textual descriptions, not expressive, limited to the lesion type, without any specific detail about the lesion characteristics. This noise introduces two major limitations: larger datasets are needed, which may be not always feasible, and algorithms perform poorly when applied to dermatology data acquired with more controlled procedures (e.g., dermoscopic images). Such limitations prevent the full exploitation of multimodal learning techniques and hinder the application of recent advances in foundation models to the dermatology domain^2–4^.

A potential strategy to address these issues is the automatic generation of clinical notes from images using LLMs, enabling better exploitation of existing unimodal datasets. Despite the availability of LLMs that are specialized for medicine, e.g., BioMedCLIP^34^ or MedImgInsights^50^, specific for dermatology, such as MONET^51^ or Derm1M^3^, or general purpose such as Generative Pre-trained Transformer (GPT), they are prone to hallucinations (i.e., incorrect outputs containing inaccurate content) and noisy outputs. Use of such inaccurate information can negatively affect MM training.

The goal of this paper is to investigate and evaluate strategies to generate reliable clinical notes describing dermatology images, using existing LLMs and metadata. The paper explores the circumstances and setups that mitigate hallucinations, to improve performance on foundation model downstream tasks. In contrast to state-of-the-art MM approaches, relying on manually curated image-text pairs or large-scale web-scraped corpora, this work introduces a learning paradigm centered on synthesized clinical notes, explicitly evaluating when and how automatically generated textual information can be reliably exploited under realistic data availability constraints.

**Fig. 1:**
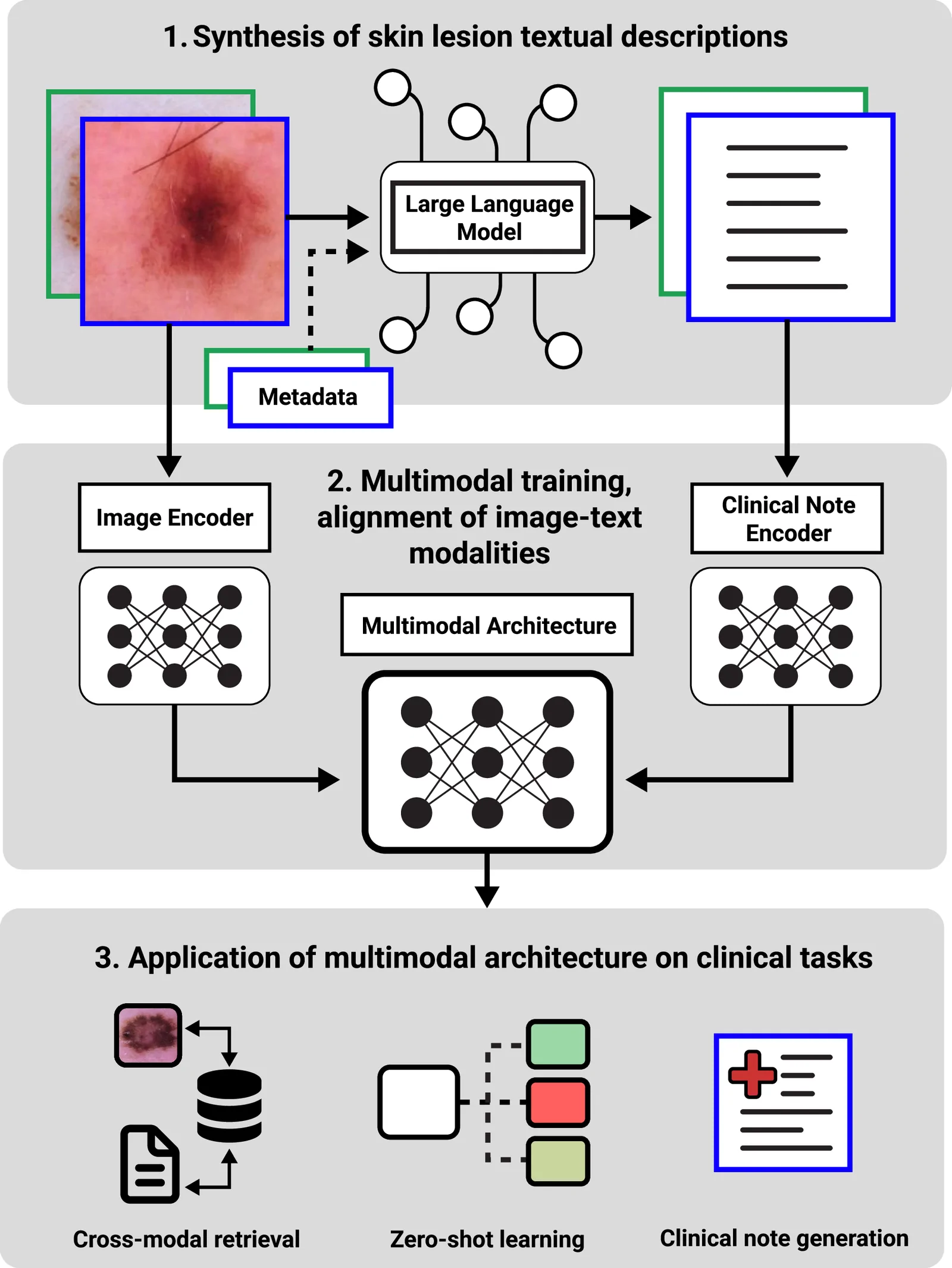
Overview of the MM architecture deployment, including the main three steps. 1. Synthesis of skin lesion clinical notes. A pre-trained LLM is used to generate clinical notes describing skin lesion images. The skin lesions are paired with the textual clinical notes, to create image-text couples. Metadata are used to synthesize clinical notes in M (filling a fixed clinical note template) and L (added to the LLM prompt) strategies, while they are not used for OL strategy. 2. Multimodal training, alignment of image-text modalities. The image-text couples are used to train an independent MM architecture, which includes specific input branches for each modality and aligns the representations of both images and clinical notes. 3. Application of MM architecture on clinical tasks. After training, the MM architecture backbone can be used to solve downstream tasks (such as cross-modal retrieval, zero-shot learning classification and clinical note generation) despite not being explicitly trained to perform these tasks.

Specifically, the paper examines multiple assumptions related to the reliability of LLM-synthesized clinical notes to support multimodal learning in dermatology. To empirically test the assumptions, we design a flexible MM architecture that integrates images and texts through three main steps, summarized in Fig. 1: (1) a pre-trained LLM generates clinical notes describing dermatology images, creating a dataset of paired image-text couples; (2) an independent MM architecture is trained to align representation of images and clinical notes, feeding specific encoders for each modality and using shared layers to create a multimodal representation; and (3) the MM architecture can be adopted to solve clinical practice tasks. The architecture is trained on a limited-size dataset of approximately 16,000 skin lesions (a small amount compared with the volume of data typically required to train foundation models) collected from six publicly available datasets and evaluated on 15 datasets, including nine external datasets, to assess generalization on new data. The architecture performance is assessed on three main clinical tasks: cross-modal retrieval, zero-shot learning, text generation. While the first two tasks are not directly optimized during training, text generation is addressed in a subsequent phase and represents a relevant direction for telemedicine applications, as demonstrated by existing dermatology-related LLMs^2,3^.

## Results

### Hypothesis and assumptions

This paper is built on key assumptions about the limitations of current LLMs and foundation models in dermatology. For each assumption, we present a hypothesis about its possible solution.

The first assumption we made is that LLMs are prone to hallucination when describing dermatological images in the absence of lesion-specific information. Consequently, the hypothesis we formulated is that a MM architecture trained using clinical notes that are structured according to a fixed template leads to higher performance. This template contains lesion-specific information collected from metadata.

The second assumption we made is that LLMs can output more detailed descriptions of skin lesions if queried non-strictly on the medical characteristics (e.g., asymmetry, color, lesion morphology). However, they may be less accurate when queried on dermatology-specific characteristics (e.g., dermatology features). Consequently, the hypothesis we formulated is that prompts targeting non-medical characteristics lead to more accurate outputs. In contrast, queries on dermatology-specific characteristics inherently lead to hallucinations.

The third assumption we made is that enriching clinical notes with lesion-related characteristics improves MM performance on targeted tasks. Consequently, the hypothesis we formulated is that clinical notes including additional details lead the model to learn a stronger multimodal representation. This results in higher performance on specific tasks.

The fourth assumption we made is that state-of-the-art foundation models, despite being trained on large datasets, show limited performance when evaluated on heterogeneous datasets. Consequently, the hypothesis we formulated is that a MM architecture trained on a reduced size dataset, 16,000 samples including primarily dermoscopic images, reaches higher performance than the current state-of-the-art foundation models. The improvement is especially evident when evaluated on heterogeneous external datasets.

### LLMs hallucinate on dermatology data if no lesion information is provided

LLMs generate noisy clinical notes on dermatology data when prompted without any lesion information.

To validate this assumption, this section analyzes the performance of clinical notes generated by four LLMs when no lesion-specific information is provided in the prompt (OL LLM). The model is compared with the same architecture trained on clinical notes generated by filling a fixed template with metadata. The performance is assessed on cross-modal retrieval (Table 1) and zero-shot learning (Table 2) tasks.

**Table 1:** Results of cross-modal retrieval task for the MM architecture, comparing the performance reached training the model with clinical notes generated filling a template with metadata (M) and clinical notes synthesized using LLMs, without any information about the lesion (OL, LLM)

	Metadata	No metadata			
Dataset	M	OL, GPT	OL, MedGemma	OL, SkinGPT4	OL, DermLIP
*Image-to-text*					
BCN20000	**0.769** ± 0.048*	0.456 ± 0.018	0.340 ± 0.016	0.380 ± 0.029	0.596 ± 0.016
derm12345	**0.886** ± 0.037*	0.460 ± 0.047	0.483 ± 0.037	0.582 ± 0.063	0.811 ± 0.012
Derm7pt	**0.766** ± 0.056*	0.397 ± 0.025	0.255 ± 0.009	0.467 ± 0.038	0.560 ± 0.035
DermNet	**0.689** ± 0.081*	0.321 ± 0.012	0.239 ± 0.005	0.211 ± 0.009	0.446 ± 0.054
FLUO_SC	**0.714** ± 0.071*	0.386 ± 0.032	0.235 ± 0.017	0.209 ± 0.035	0.379 ± 0.021
MRA_MIDAS	**0.538** ± 0.038*	0.362 ± 0.012	0.315 ± 0.012	0.293 ± 0.012	0.468 ± 0.015
HAM10000	**0.774** ± 0.016*	0.478 ± 0.035	0.445 ± 0.031	0.493 ± 0.048	0.705 ± 0.016
SKINL2	**0.774** ± 0.048*	0.408 ± 0.034	0.392 ± 0.035	0.406 ± 0.032	0.631 ± 0.018
Fitzpatrick17k	**0.596** ± 0.027*	0.330 ± 0.006	0.271 ± 0.011	0.306 ± 0.020	0.398 ± 0.020
HIBA	**0.569** ± 0.038*	0.352 ± 0.020	0.345 ± 0.018	0.364 ± 0.034	0.529 ± 0.016
PAD UFES 20	**0.596** ± 0.032*	0.335 ± 0.029	0.264 ± 0.014	0.230 ± 0.018	0.351 ± 0.015
SD198	**0.446** ± 0.059*	0.367 ± 0.026	0.222 ± 0.014	0.253 ± 0.018	0.338 ± 0.017
MSK	**0.638** ± 0.021*	0.409 ± 0.023	0.376 ± 0.019	0.396 ± 0.027	0.563 ± 0.007
Milk10k (clinic)	**0.536** ± 0.051*	0.296 ± 0.009	0.259 ± 0.011	0.237 ± 0.013	0.392 ± 0.013
Milk10k (dermo)	**0.638** ± 0.045*	0.325 ± 0.013	0.264 ± 0.015	0.257 ± 0.016	0.435 ± 0.026
*Text-to-image*					
BCN20000	**0.734** ± 0.030*	0.297 ± 0.011	0.339 ± 0.010	0.255 ± 0.007	0.482 ± 0.030
derm12345	**0.856** ± 0.020*	0.403 ± 0.053	0.555 ± 0.048	0.33 ± 0.024	0.656 ± 0.051
Derm7pt	**0.623** ± 0.097*	0.215 ± 0.042	0.195 ± 0.009	0.16 ± 0.007	0.291 ± 0.020
DermNet	**0.599** ± 0.079*	0.235 ± 0.016	0.166 ± 0.009	0.219 ± 0.005	0.364 ± 0.019
FLUO_SC	**0.592** ± 0.037*	0.240 ± 0.012	0.174 ± 0.035	0.216 ± 0.006	0.294 ± 0.011
MRA_MIDAS	**0.668** ± 0.037*	0.280 ± 0.010	0.296 ± 0.013	0.257 ± 0.007	0.445 ± 0.016
HAM10000	0.289 ± 0.008	0.458 ± 0.035	**0.571** ± **0.028**	0.253 ± 0.003	0.484 ± 0.036
SKINL2	**0.742** ± 0.054*	0.339 ± 0.025	0.406 ± 0.021	0.272 ± 0.012	0.550 ± 0.017
Fitzpatrick17k	**0.589** ± 0.053*	0.241 ± 0.012	0.169 ± 0.003	0.224 ± 0.033	0.373 ± 0.027
HIBA	**0.747** ± 0.028*	0.292 ± 0.016	0.354 ± 0.014	0.263 ± 0.010	0.507 ± 0.026
PAD UFES 20	**0.626** ± 0.041*	0.231 ± 0.007	0.169 ± 0.009	0.216 ± 0.011	0.331 ± 0.023
SD198	**0.564** ± 0.057*	0.235 ± 0.020	0.218 ± 0.027	0.176 ± 0.012	0.336 ± 0.014
MSK	**0.747** ± 0.027*	0.330 ± 0.020	0.379 ± 0.022	0.280 ± 0.010	0.502 ± 0.034
Milk10k (clinic)	**0.677** ± 0.033*	0.234 ± 0.016	0.214 ± 0.011	0.236 ± 0.012	0.374 ± 0.023
Milk10k (dermo)	**0.677** ± 0.033*	0.234 ± 0.016	0.214 ± 0.011	0.236 ± 0.012	0.374 ± 0.023

The header reports if a setup involves the application of metadata (Metadata, No Metadata). Performance is evaluated on 15 datasets (top six internal partition, the rest external partition) using mAP for Image-to-Text (upper part) and Text-to-Image (lower part) cross-modal retrieval settings across multiple datasets, reporting average and standard deviation for ten repetitions; * indicates statistically significant differences (*p* < 0.05, Wilcoxon test). The method reaching the highest average is marked in bold. In case of equal values, the method reaching the lowest standard deviation is selected.

**Table 2:** Results of zero-shot learning task for the MM architecture, comparing the performance reached training the model with clinical notes generated filling a template with metadata (M) and clinical notes synthesized using LLMs, without any information about the lesion (OL, LLM)

	Metadata	No metadata			
Dataset	M	OL, GPT	OL, MedGemma	OL, SkinGPT4	OL, DermLIP
*8-classes*					
BCN20000	**0.554** ± **0.037**	0.160 ± 0.088	0.167 ± 0.042	0.398 ± 0.057	0.554 ± 0.092
derm12345	0.598 ± 0.216	0.187 ± 0.211	0.058 ± 0.014	0.268 ± 0.211	**0.799** ± 0.059*
Derm7pt	0.261 ± 0.049	0.187 ± 0.135	0.239 ± 0.099	**0.484** ± **0.099**	0.441 ± 0.196
DermNet	**0.562** ± 0.100*	0.242 ± 0.077	0.392 ± 0.045	0.288 ± 0.061	0.314 ± 0.135
FLUO_SC	**0.710** ± 0.094*	0.292 ± 0.113	0.253 ± 0.019	0.285 ± 0.113	0.504 ± 0.262
MRA_MIDAS	**0.390** ± **0.056**	0.202 ± 0.066	0.150 ± 0.031	0.216 ± 0.053	0.361 ± 0.058
HAM10000	0.571 ± 0.105	0.151 ± 0.149	0.067 ± 0.015	0.335 ± 0.141	**0.705** ± 0.029*
SKINL2	0.583 ± 0.105	0.207 ± 0.111	0.254 ± 0.034	0.271 ± 0.134	**0.635** ± **0.076**
Fitzpatrick17k	**0.398** ± **0.030**	0.219 ± 0.122	0.121 ± 0.015	0.378 ± 0.102	0.384 ± 0.100
HIBA	0.317 ± 0.078	0.147 ± 0.065	0.175 ± 0.019	0.212 ± 0.066	**0.391** ± **0.112**
PAD UFES 20	**0.486** ± 0.044*	0.229 ± 0.050	0.319 ± 0.048	0.220 ± 0.026	0.365 ± 0.130
SD198	**0.356** ± 0.032*	0.199 ± 0.058	0.190 ± 0.025	0.323 ± 0.077	0.272 ± 0.080
MSK	0.442 ± 0.048	0.139 ± 0.080	0.126 ± 0.036	0.297 ± 0.082	**0.501** ± 0.045*
Milk10k (clinic)	**0.410** ± **0.066**	0.185 ± 0.068	0.219 ± 0.024	0.132 ± 0.015	0.356 ± 0.096
Milk10k (dermo)	0.566 ± 0.027	0.122 ± 0.056	0.171 ± 0.027	0.205 ± 0.027	**0.587** ± **0.027**
*15-classes*					
BCN20000	0.269 ± 0.023	0.077 ± 0.034	0.115 ± 0.038	0.238 ± 0.047	**0.528** ± 0.084*
derm12345	**0.448** ± 0.026*	0.057 ± 0.044	0.091 ± 0.045	0.051 ± 0.018	0.237 ± 0.034
Derm7pt	0.177 ± 0.034	0.233 ± 0.161	0.166 ± 0.073	**0.418** ± **0.124**	0.400 ± 0.180
DermNet	**0.504** ± 0.086*	0.193 ± 0.052	0.335 ± 0.079	0.117 ± 0.069	0.299 ± 0.066
FLUO_SC	**0.665** ± 0.151*	0.177 ± 0.120	0.226 ± 0.063	0.088 ± 0.077	0.368 ± 0.190
MRA_MIDAS	0.203 ± 0.055	0.130 ± 0.033	0.151 ± 0.038	0.077 ± 0.015	**0.299** ± **0.050**
HAM10000	0.124 ± 0.016	0.053 ± 0.013	0.048 ± 0.008	0.155 ± 0.074	**0.691** ± 0.041*
SKINL2	0.275 ± 0.046	0.153 ± 0.077	0.196 ± 0.056	0.081 ± 0.009	**0.546** ± 0.059*
Fitzpatrick17k	0.298 ± 0.041	0.121 ± 0.072	0.081 ± 0.034	0.100 ± 0.072	**0.334** ± **0.144**
HIBA	0.142 ± 0.041	0.097 ± 0.048	0.188 ± 0.035	0.057 ± 0.023	**0.359** ± 0.131*
PAD UFES 20	**0.420** ± 0.073*	0.186 ± 0.059	0.291 ± 0.058	0.064 ± 0.009	0.303 ± 0.095
SD198	**0.270** ± **0.036**	0.117 ± 0.039	0.149 ± 0.035	0.121 ± 0.012	0.246 ± 0.050
MSK	**0.232** ± 0.020*	0.069 ± 0.028	0.108 ± 0.042	0.079 ± 0.033	0.140 ± 0.031
Milk10k (clinic)	**0.299** ± **0.096**	0.145 ± 0.066	0.198 ± 0.029	0.028 ± 0.025	0.257 ± 0.091
Milk10k (dermo)	0.423 ± 0.075	0.063 ± 0.024	0.109 ± 0.031	0.078 ± 0.054	**0.509** ± 0.022*

The header reports if a setup involves the application of metadata (Metadata, No Metadata). Performance is evaluated on 15 datasets (top six internal partition, the rest external partition) using weighted F1-score for 8-class (upper part) and 15-class (lower part) classification settings across multiple datasets, reporting average and standard deviation for ten repetitions; * indicates statistically significant differences (*p* < 0.05, Wilcoxon test). The method reaching the highest average is marked in bold. In case of equal values, the method reaching the lowest standard deviation is selected.

Results on cross-modal retrieval show a clear and systematic advantage for M over all OL variants. Two setups are tested: Image-to-text, using images as input modality to retrieve clinical notes; Text-to-image, using clinical notes as input to retrieve images. In both cases, M achieves the highest performance on every dataset, including both internal and external partitions. The performance gaps are large and statistically significant, with absolute differences in mAP ranging from 0.10 to 0.40 compared to OL, with only isolated exceptions (e.g., HAM10000) where OL partially closes the gap. The performance decrease associated with OL is more evident on external datasets, indicating poor generalization when models are trained on hallucinated clinical notes. Among the OL settings, DermLIP consistently reaches the strongest results. Still, this setup systematically achieves poorer performance compared to M in most cases, confirming that even dermatology-oriented LLMs cannot compensate for the lack of lesion-specific when prompting the model.

Results on zero-shot learning partially confirm the findings identified in the previous task, despite the margins between M and OL are reduced and OL models perform better on some datasets. On both 8- and 15-class setups, M and OL DermLIP generally achieves comparable performance, with statistically significant improvements on several datasets on both sides. While DermLIP occasionally matches or exceeds M on specific datasets and class granularities, OL models overall exhibit high variance (i.e., higher standard deviation) and inconsistent behavior, reflecting noisy and unreliable semantic supervision.

### Lesion descriptions can be queried

LLMs can generate detailed descriptions of dermatological data.

To validate this assumption, this section analyzes the clinical notes synthesized with LLMs, considering two strategies: L and OL. L involves prompting LLMs including metadata information in the query. OL involves prompting LLMs without including metadata information in the query. This set of experiments aims to analyze three aspects: whether LLMs can produce dermatology-related text, whether LLMs can produce valuable dermatology-related text related to the lesion type, whether LLMs can identify other relevant dermatology-related features.

Table 3 summarizes the results on the potential of LLMs to provide information related to the lesion, whether correct or not, when synthesizing clinical notes. The Table reports the percentage of occurrences including keywords related to a lesion type in the synthesized notes. Results show that strategy L leads to higher number of notes including lesion type keywords, whereas the OL setup frequently fails to produce meaningful dermatological content, especially considering LLM specialized in dermatology (SkinGPT4 and DermLIP).

**Table 3:** Overview on the LLM potential to generate dermatology-related clinical notes, focusing on the capability of including a lesion (whether correct or not) in the synthesized clinical notes

	GPT		MedGemma		SkinGPT4		DermLIP	
Dataset	L	OL	L	OL	L	OL	L	OL
BCN20000	1.000	0.992	0.972	0.958	0.844	0.005	0.684	0.418
derm12345	1.000	0.984	0.985	0.958	0.707	0.004	0.494	0.353
Derm7pt	1.000	1.000	0.916	0.960	0.788	0.010	0.575	0.654
DermNet	1.000	0.974	0.916	0.965	0.889	0.041	0.796	0.871
FLUO_SC	1.000	0.985	0.972	0.935	0.820	0.010	0.464	0.106
MRA_MIDAS	0.993	0.983	0.954	0.940	0.729	0.002	0.762	0.596
HAM10000	1.000	0.999	0.977	0.970	0.774	0.014	0.472	0.559
SKINL2	1.000	0.995	0.976	0.953	0.873	0.014	0.613	0.500
Fitzpatrick17k	1.000	0.976	0.907	0.925	0.784	0.027	0.811	0.656
HIBA	1.000	0.993	0.969	0.953	0.789	0.011	0.709	0.449
PAD UFES 20	1.000	0.972	0.987	0.934	0.765	0.010	0.669	0.069
SD198	1.000	0.987	0.938	0.965	0.757	0.049	0.558	0.491
MSK	1.000	0.992	0.978	0.958	0.778	0.008	0.542	0.481
Milk10k_clinic	1.000	0.983	0.973	0.928	0.705	0.015	0.617	0.146
Milk10k_dermo	1.000	0.985	0.982	0.944	0.737	0.010	0.750	0.232

Table includes two types of strategies, L (prompting a LLM with metadata), OL (prompting a LLM without metadata), evaluated on each dataset. Results are grouped by the LLM used to synthesize data, so that it is possible to compare strategies L and OL. For each setup, the percentage of correct lesion types included in the notes is reported in terms of accuracy.

The second set of experiments aims to investigate if the lesion reported in the clinical notes is correct. Table 4 summarizes the results on the potential of LLMs to provide correct information related to the lesion when synthesizing clinical notes. The Table reports the percentage of occurrences including the correct lesion type in the synthesized notes. Again, results show that strategy L, guided by metadata, leads to higher number of notes including lesion type keywords. In particular, L strategy leads to clinical notes including the right class very often when using GPT and MedGemma, which are trained with a larger variety of prompts, while the number of correct classes is lower on the two other LLMs. This result can be explained considering the query: while GPT and MedGemma are trained with a larger variety of prompts and allow larger input texts, SkinGPT4 and DermLIP are trained with specific prompts, limiting their variety. Despite SkinGPT4 (showing a dramatic drop in performance close to 99%), the other LLMs generally show a 66% performance drop when metadata are not adopted to query the model.

**Table 4:** Overview of the LLM potential to generate dermatology-related clinical notes, focusing on the capability of including the right type of lesion in the synthesized clinical notes

	GPT		MedGemma		SkinGPT4		DermLIP	
Dataset	L	OL	L	OL	L	OL	L	OL
BCN20000	1.000	0.284	0.936	0.353	0.793	0.001	0.619	0.201
derm12345	0.996	0.182	0.945	0.631	0.519	0.000	0.405	0.270
Derm7pt	1.000	0.605	0.911	0.291	0.785	0.005	0.479	0.054
DermNet	1.000	0.268	0.916	0.326	0.878	0.011	0.502	0.397
FLUO_SC	0.950	0.145	0.910	0.367	0.724	0.000	0.248	0.036
MRA_MIDAS	0.993	0.234	0.951	0.355	0.725	0.001	0.609	0.191
HAM10000	1.000	0.177	0.926	0.489	0.546	0.002	0.395	0.430
SKINL2	1.000	0.203	0.976	0.538	0.854	0.005	0.396	0.231
Fitzpatrick17k	1.000	0.461	0.901	0.248	0.755	0.013	0.688	0.408
HIBA	1.000	0.274	0.905	0.345	0.759	0.002	0.605	0.234
PAD UFES 20	1.000	0.134	0.976	0.395	0.729	0.000	0.540	0.029
SD198	1.000	0.279	0.934	0.212	0.743	0.009	0.310	0.226
MSK	1.000	0.298	0.951	0.379	0.622	0.003	0.467	0.290
Milk10k_clinic	0.965	0.175	0.934	0.296	0.620	0.004	0.488	0.074
Milk10k_dermo	0.965	0.140	0.937	0.264	0.646	0.003	0.676	0.110

Table includes two types of strategies, L (prompting a LLM with metadata), OL (prompting a LLM without metadata), evaluated on each dataset. Results are grouped by the LLM used to synthesize data, so that it is possible to compare strategies L and OL. For each setup, the percentage of correct lesion types included in the notes is reported in terms of accuracy.

Despite showing a good capability of reporting the right lesion type, LLMs show limited capability to provide further information related to the lesion features. Table 5 reports the LLM performance to identify nine specific lesion-related features on the PH2 dataset, which is annotated with those characteristics. The results show a clear discrepancy between feature types. Non-medical characteristics, such as symmetry and color, are generally described more accurately than dermatology-specific features, such as pigment patterns, dots, or streaks, which are poorly reported considering each setup. This behavior supports our hypothesis that LLMs are more reliable when queried about general visual attributes, while dermatology-specific prompts are more prone to hallucinations. Again, GPT and MedGemma reach the highest performance, but again the reason for this performance gap can be attributed to the prompt structure to query the architectures, which is limited in the dermatology-specialized LLMs.

**Table 5:** Overview on the LLM potential to identify dermatology-relevant features (excluding the lesion type)

	GPT		MedGemma		SkinGPT4		DermLIP	
Feature	L	OL	L	OL	L	OL	L	OL
Symmetry/Asymmetry	0.866	0.53	0.696	0.489	0.027	0	0	0
Pigment	0.558	0.74	0.259	0.457	0.445	0.033	0	0
Dot	0.652	0.575	0.482	0.596	0.595	0.6	0.583	0.583
Streak	0.256	0.405	0.052	0	0	0	0	0
White	0	0	0	0	0.051	0.075	0	0
Red	0	0.278	0	0.154	0.25	0.21	0	0
Light Brown	0.633	0.014	0.055	0	0.042	0.131	0.042	0
Dark Brown	0.763	0.085	0.885	0.625	0.562	0.25	0	0.013
Brown	0.807	0.82	0.824	0.792	0.674	0.68	0.082	0.13
Gray	0.038	0.038	0	0	0.025	0.037	0	0
Black	0.472	0.4	0.1	0.238	0.3	0.262	0.085	0.14

Table includes two types of strategies, L (prompting a LLM with metadata), OL (prompting a LLM without metadata), evaluated on each dataset, evaluated on PH2 dataset. Results are grouped by the LLM used to synthesize data, so that it is possible to compare strategies L and OL. For each setup, the F1-score for each feature is reported.

The capability of LLMs to query the right class is reported in Figure Supplementary Fig. 1 (Section Hallucinations and noisy LLM outputs, Supplementary Materials), showing confusion matrices about L and OL strategies, considering each LLM.

### Enriched clinical notes improve MM performance

Enriching clinical notes with lesion-related characteristics improves MM performance.

To validate this assumption, this section compares the performance reached by L, LLM-based notes, generated with metadata, with M, metadata-based notes, on cross-modal retrieval and zero-shot learning. The comparison aims to analyze if richer reports (L), describing other aspects of the lesion rather than the type, lead to higher performance, involving two tasks: cross-modal retrieval (Table 6, both image-to-text and text-to-image setups) and zero-shot learning (Table 7, both 8- and 15-classes).

**Table 6:** Results on the cross-modal retrieval task for the MM architecture, comparing the performance reached training the model with clinical notes generated filling a template with metadata (M) and clinical notes synthesized prompting a LLM providing lesion metadata (L; GPT, MedGemma, SkinGPT4, DermLIP)

	Template-based	LLM-based			
Dataset	M	L, GPT	L, MedGemma	L, SkinGPT4	L, DermLIP
*Image-to-text*					
BCN20000	0.769 ± 0.048	0.741 ± 0.022	0.785 ± 0.013	0.758 ± 0.013	**0.791** ± **0.012**
derm12345	**0.886** ± **0.037**	0.846 ± 0.011	0.872 ± 0.009	0.861 ± 0.006	0.879 ± 0.007
Derm7pt	0.766 ± 0.056	0.711 ± 0.027	**0.785** ± **0.032**	0.751 ± 0.01	0.774 ± 0.023
DermNet	0.689 ± 0.081	0.612 ± 0.030	0.717 ± 0.032	0.696 ± 0.017	**0.717** ± **0.018**
FLUO_SC	0.714 ± 0.071	0.682 ± 0.017	0.737 ± 0.016	0.726 ± 0.016	**0.736** ± **0.024**
MRA_MIDAS	0.538 ± 0.038	0.538 ± 0.013	0.591 ± 0.016*	0.526 ± 0.009	**0.597** ± 0.015*
HAM10000	0.774 ± 0.016	0.754 ± 0.016	0.785 ± 0.009	0.767 ± 0.011	**0.788** ± 0.007*
SKINL2	0.774 ± 0.048	0.750 ± 0.019	**0.808** ± 0.013*	0.767 ± 0.017	0.804 ± 0.016*
Fitzpatrick17k	0.596 ± 0.027	0.512 ± 0.036	0.575 ± 0.030	0.517 ± 0.016	**0.620** ± 0.012*
HIBA	0.569 ± 0.038	0.524 ± 0.045	0.577 ± 0.035	0.569 ± 0.035	**0.593** ± **0.036**
PAD UFES 20	0.596 ± 0.032	0.575 ± 0.022	0.622 ± 0.020*	0.607 ± 0.022	**0.633** ± 0.022*
SD198	0.446 ± 0.059	0.411 ± 0.041	0.440 ± 0.037	0.437 ± 0.037	**0.464** ± **0.034**
MSK	0.638 ± 0.021	0.613 ± 0.015	0.635 ± 0.009	0.624 ± 0.013	**0.657** ± 0.005*
Milk10k (clinic)	0.536 ± 0.051	0.499 ± 0.024	**0.573** ± 0.035*	0.522 ± 0.032	0.571 ± 0.032
Milk10k (dermo)	0.638 ± 0.045	0.619 ± 0.024	**0.666** ± **0.013**	0.61 ± 0.016	0.660 ± 0.012
*Text-to-image*					
BCN20000	0.734 ± 0.030	0.580 ± 0.016	0.711 ± 0.017	0.510 ± 0.004	**0.756** ± 0.012*
derm12345	0.856 ± 0.020	0.813 ± 0.007	0.840 ± 0.012	0.778 ± 0.020	**0.867** ± **0.005**
Derm7pt	0.623 ± 0.097	0.448 ± 0.043	0.613 ± 0.036	0.371 ± 0.015	**0.681** ± 0.008*
DermNet	0.599 ± 0.079	0.357 ± 0.033	0.556 ± 0.034	0.520 ± 0.022	**0.650** ± 0.007*
FLUO_SC	0.592 ± 0.037	0.374 ± 0.025	0.549 ± 0.044	0.451 ± 0.015	**0.616** ± 0.007*
MRA_MIDAS	0.668 ± 0.037	0.522 ± 0.027	0.650 ± 0.024	0.518 ± 0.010	**0.712** ± 0.012*
HAM10000	0.289 ± 0.008	0.370 ± 0.035	0.475 ± 0.039*	0.512 ± 0.047*	**0.578** ± 0.019*
SKINL2	0.742 ± 0.054	0.598 ± 0.022	0.711 ± 0.024	0.669 ± 0.014	**0.782** ± 0.005*
Fitzpatrick17k	0.589 ± 0.053	0.401 ± 0.030	0.619 ± 0.017	0.471 ± 0.021	**0.640** ± 0.010*
HIBA	0.747 ± 0.028	0.620 ± 0.013	0.723 ± 0.014	0.557 ± 0.004	**0.771** ± 0.011*
PAD UFES 20	0.626 ± 0.041	0.551 ± 0.015	0.613 ± 0.024	0.433 ± 0.017	**0.661** ± 0.015*
SD198	0.564 ± 0.057	0.471 ± 0.021	0.554 ± 0.029	0.509 ± 0.019	**0.622** ± 0.012*
MSK	0.747 ± 0.027	0.607 ± 0.015	0.740 ± 0.017	0.567 ± 0.004	**0.767** ± 0.012*
Milk10k (clinic)	0.677 ± 0.033	0.559 ± 0.017	0.673 ± 0.023	0.432 ± 0.012	**0.707** ± 0.015*
Milk10k (dermo)	0.677 ± 0.033	0.559 ± 0.017	0.673 ± 0.023	0.432 ± 0.012	**0.707** ± 0.015*

Performance is reported as average mAP ± standard deviation (ten repetitions) for image-to-text (upper part) and text-to-image (lower part) retrieval across datasets; * denotes statistically significant differences between M and L (*p* < 0.05, Wilcoxon test). The method reaching the highest average is marked in bold. In case of equal values, the method reaching the lowest standard deviation is selected.

**Table 7:** Results on the zero-shot learning task for the MM architecture, comparing the performance reached training the model with clinical notes generated filling a template with metadata (M) and clinical notes synthesized prompting a LLM providing lesion metadata (L; GPT, MedGemma, SkinGPT4, DermLIP)

	Template-based	LLM-based			
Dataset	M	L, GPT	L, MedGemma	L, SkinGPT4	L, DermLIP
*8-classes*					
BCN20000	0.554 ± 0.037	**0.738** ± 0.014*	0.718 ± 0.050*	0.724 ± 0.045*	0.691 ± 0.053*
derm12345	0.598 ± 0.216	0.811 ± 0.026*	0.837 ± 0.016*	0.840 ± 0.017*	**0.845** ± 0.017*
Derm7pt	0.261 ± 0.049	**0.744** ± 0.036*	0.664 ± 0.125*	0.669 ± 0.072*	0.670 ± 0.094*
DermNet	0.562 ± 0.100	0.671 ± 0.064*	0.729 ± 0.103*	**0.784** ± 0.080*	0.763 ± 0.077*
FLUO_SC	0.710 ± 0.094	0.743 ± 0.052	0.780 ± 0.061	**0.839** ± 0.033*	0.753 ± 0.082
MRA_MIDAS	0.390 ± 0.056	0.468 ± 0.026*	0.501 ± 0.021*	0.474 ± 0.017*	**0.501** ± 0.018*
HAM10000	0.571 ± 0.105	0.734 ± 0.012*	0.726 ± 0.018*	**0.735** ± 0.015*	0.732 ± 0.015*
SKINL2	0.583 ± 0.105	**0.772** ± 0.025*	0.771 ± 0.029*	0.740 ± 0.051*	0.744 ± 0.023*
Fitzpatrick17k	0.398 ± 0.030	0.516 ± 0.050*	0.533 ± 0.048*	0.525 ± 0.051*	**0.545** ± 0.038*
HIBA	0.317 ± 0.078	0.464 ± 0.073*	**0.503** ± 0.041*	0.499 ± 0.065*	0.496 ± 0.031*
PAD UFES 20	0.486 ± 0.044	0.523 ± 0.027	0.542 ± 0.017*	0.567 ± 0.019*	**0.570** ± 0.018*
SD198	0.356 ± 0.032	0.367 ± 0.042	0.353 ± 0.036	0.384 ± 0.040	**0.429** ± 0.027*
MSK	0.442 ± 0.048	**0.570** ± 0.015*	0.550 ± 0.025*	0.573 ± 0.024*	0.569 ± 0.022*
Milk10k (clinic)	0.410 ± 0.066	0.506 ± 0.027*	**0.534** ± 0.025*	0.510 ± 0.026*	0.502 ± 0.023*
Milk10k (dermo)	0.566 ± 0.027	**0.642** ± 0.010*	0.627 ± 0.013*	0.613 ± 0.016*	0.618 ± 0.009*
*15-classes*					
BCN20000	0.269 ± 0.023	0.386 ± 0.015*	0.419 ± 0.107*	0.511 ± 0.087*	**0.590** ± 0.009*
derm12345	0.448 ± 0.026	0.450 ± 0.007	0.440 ± 0.056	0.156 ± 0.045	**0.464** ± **0.103**
Derm7pt	0.177 ± 0.034	0.653 ± 0.047*	0.395 ± 0.151*	**0.679** ± 0.020*	0.402 ± 0.116*
DermNet	0.504 ± 0.086	0.570 ± 0.053*	0.537 ± 0.106	**0.739** ± 0.062*	0.642 ± 0.046*
FLUO_SC	0.665 ± 0.151	0.436 ± 0.095	0.282 ± 0.104	0.650 ± 0.123	**0.727** ± **0.061**
MRA_MIDAS	0.203 ± 0.055	0.233 ± 0.014	0.256 ± 0.068*	0.314 ± 0.043*	**0.391** ± 0.033*
HAM10000	0.124 ± 0.016	0.156 ± 0.009*	0.341 ± 0.141*	0.279 ± 0.134*	**0.554** ± 0.121*
SKINL2	0.275 ± 0.046	0.373 ± 0.016*	0.426 ± 0.088*	0.425 ± 0.057*	**0.525** ± 0.067*
Fitzpatrick17k	0.298 ± 0.041	0.215 ± 0.019	0.274 ± 0.086	**0.446** ± 0.013*	0.421 ± 0.021*
HIBA	0.142 ± 0.041	0.207 ± 0.032*	0.208 ± 0.094	0.300 ± 0.029*	**0.301** ± 0.087*
PAD UFES 20	0.420 ± 0.073	0.336 ± 0.048	0.281 ± 0.053	0.439 ± 0.066	**0.541** ± 0.019*
SD198	0.270 ± 0.036	0.275 ± 0.021	0.237 ± 0.033	0.268 ± 0.035	**0.285** ± **0.018**
MSK	0.232 ± 0.020	**0.313** ± 0.012*	0.235 ± 0.029	0.253 ± 0.009*	0.254 ± 0.055
Milk10k (clinic)	0.299 ± 0.096	0.374 ± 0.027*	0.300 ± 0.062	0.368 ± 0.036*	**0.422** ± 0.029*
Milk10k (dermo)	0.423 ± 0.075	0.462 ± 0.009*	0.379 ± 0.083	0.478 ± 0.032*	**0.516** ± 0.011*

Performance is reported as average F1-score ± standard deviation (ten repetitions) for 8-classes setup (upper part) and the 15-classes (lower part) retrieval across datasets; * denotes statistically significant differences between M and L (*p* < 0.05, Wilcoxon test). The method reaching the highest average is marked in bold. In case of equal values, the method reaching the lowest standard deviation is selected.

Results on the cross-modal retrieval task show that L models generally match or outperform the metadata-only baseline (M), with statistically significant improvements observed across several datasets and configurations, particularly when using MedGemma and DermLIP. The performance improvement is more pronounced in the text-to-image setting, where enriched clinical notes substantially improve retrieval performance, indicating a stronger semantic grounding of visual features. Notably, the statistically significant differences in performance are identified in external datasets, suggesting that LLM-based strategies better generalize on independent data.

Zero-shot learning results further reinforce these findings. Considering the 8-class setup, all L models significantly outperform M across nearly all datasets, with most models across all LLMs showing a difference in performance that is statistically significant. Considering the 15-class setting, performance differences are amplified: while M struggles to scale with increased class granularity, L models, particularly DermLIP and SkinGPT4, achieve large and often statistically significant gains. In this case, the statistically significant differences in performance can be identified in almost every dataset, despite if selected from the internal or the external partition.

Two relevant considerations can be made considering the results shown in this Section and in the previous one (Lesion descriptions can be queried). First, dermatology characteristics, not strictly related to the lesion type, have a limited influence on the modality alignment, since DermLIP shows a limited capability to identify the right characteristics, despite often reaching the highest performance on cross-modal retrieval and zero-shot learning. Second, the L strategy outperforms M, despite M shows a systematic advantage in label accuracy: clinical notes generated with M explicitly encode the correct lesion type in every note, whereas L-generated notes do not always include the correct class, which information is used to prompt the LLM generating the note, as shown in Table 4.

### MM architecture reaches higher performance than state-of-the-art dermatology foundation models

The MM architecture generally achieves higher performance than compared state-of-the-art foundation models on cross-modal retrieval and zero-shot learning tasks.

To validate this assumption, this section compares the performance reached by L with the performance of state-of-the-art foundation models (i.e., BioMedCLIP, MedImageInsights, MONET, and Derm1M), aiming to analyze if a MM network trained with limited size dataset, primarily dermoscopic images and valuable clinical descriptions of images leads to higher performance. The comparison involves two tasks, cross-modal retrieval (Table 8) and zero-shot learning (Table 9), and presents two the following characteristics: (i) the compared models are trained on datasets ranging from 100,000 to over one million image-text pairs, while the MM architecture on 16,000 samples collected from six repositories; (ii) cross-modal retrieval and concept-based classification are not the primary optimization objectives in both cases; (iii) the MM architecture is primarly trained on dermatology images, with clinical notes describing only the lesion instead of including noisy text. Despite the MM architecture reaching higher performance on most datasets, the last characteristics leads to larger performance gaps on dermoscopic datasets, while the performance difference is lighter on clinical ones.

**Table 8:** Results on the cross-modal retrieval task for the MM architecture, comparing the performance of state-of-the-art foundation models (BioMedCLIP, MedImageInsights, MONET, Derm1M) with the performance reached with our framework, involving training the model with clinical notes synthesized prompting a LLM providing lesion metadata (L; GPT, MedGemma, SkinGPT4, DermLIP)

	State-of-the-art Foundation Models				Ours			
	BioMedClip	MedImageInsights	MONET	Derm1M	GPT	MedGemma	SkinGPT4	DermLIP
*Image-to-text*								
BCN20000	0.320	0.682	0.333	0.403	0.741 ± 0.022	0.785 ± 0.013	0.758 ± 0.013	**0.791** ± **0.012**
derm12345	0.534	0.751	0.443	0.444	0.846 ± 0.011	0.872 ± 0.009	0.861 ± 0.006	**0.879** ± **0.007**
Derm7pt	0.168	0.501	0.23	0.411	0.711 ± 0.027	**0.785** ± **0.032**	0.751 ± 0.01	0.774 ± 0.023
DermNet	0.209	0.483	0.308	0.438	0.612 ± 0.030	0.717 ± 0.032	0.696 ± 0.017	**0.717** ± **0.018**
FLUO_SC	0.236	0.590	0.311	0.422	0.682 ± 0.017	**0.737** ± **0.016**	0.726 ± 0.016	0.736 ± 0.024
MRA_MIDAS	0.289	0.400	0.288	0.307	0.538 ± 0.013	0.591 ± 0.016	0.526 ± 0.009	**0.597** ± **0.015**
HAM10000	0.47	0.752	0.374	0.431	0.754 ± 0.016	0.785 ± 0.009	0.767 ± 0.011	**0.788** ± **0.007**
SKINL2	0.407	0.686	0.407	0.43	0.750 ± 0.019	**0.808** ± **0.013**	0.767 ± 0.017	0.804 ± 0.016
Fitzpatrick17k	0.212	0.406	0.304	0.331	0.512 ± 0.036	0.575 ± 0.030	0.517 ± 0.016	**0.620** ± **0.012**
HIBA	0.351	0.528	0.325	0.425	0.524 ± 0.045	0.577 ± 0.035	0.569 ± 0.035	**0.593** ± **0.036**
PAD UFES 20	0.271	**0.708**	0.416	0.49	0.575 ± 0.022	0.622 ± 0.020	0.607 ± 0.022	0.633 ± 0.022
SD198	0.217	**0.474**	0.267	0.333	0.411 ± 0.041	0.440 ± 0.037	0.437 ± 0.037	0.464 ± 0.034
MSK	0.383	0.597	0.336	0.398	0.613 ± 0.015	0.635 ± 0.009	0.624 ± 0.013	**0.657** ± **0.005**
Milk10k clinic	0.280	0.457	0.359	0.440	0.499 ± 0.024	**0.573** ± **0.035**	0.522 ± 0.032	0.571 ± 0.032
Milk10k dermo	0.278	0.564	0.329	0.417	0.619 ± 0.024	**0.666** ± **0.013**	0.61 ± 0.016	0.660 ± 0.012
*Text-to-image*								
BCN20000	0.320	0.588	0.397	0.463	0.580 ± 0.016	0.711 ± 0.017	0.510 ± 0.004	**0.756** ± **0.012**
derm12345	0.507	0.769	0.645	0.664	0.813 ± 0.007	0.840 ± 0.012	0.778 ± 0.020	**0.867** ± **0.005**
Derm7pt	0.205	0.525	0.237	0.306	0.448 ± 0.043	0.613 ± 0.036	0.371 ± 0.015	**0.681** ± **0.008**
DermNet	0.167	0.438	0.193	0.263	0.357 ± 0.033	0.556 ± 0.034	0.520 ± 0.022	**0.650** ± **0.007**
FLUO_SC	0.161	0.426	0.196	0.297	0.374 ± 0.025	0.549 ± 0.044	0.451 ± 0.015	**0.616** ± **0.007**
MRA_MIDAS	0.285	0.584	0.35	0.41	0.522 ± 0.027	0.650 ± 0.024	0.518 ± 0.010	**0.712** ± **0.012**
HAM10000	0.517	**0.711**	0.506	0.571	0.370 ± 0.035	0.475 ± 0.039	0.512 ± 0.047	0.578 ± 0.019
SKINL2	0.367	0.654	0.459	0.502	0.598 ± 0.022	0.711 ± 0.024	0.669 ± 0.014	**0.782** ± **0.005**
Fitzpatrick17k	0.186	0.443	0.223	0.291	0.401 ± 0.030	0.619 ± 0.017	0.471 ± 0.021	**0.640** ± **0.010**
HIBA	0.323	0.597	0.409	0.478	0.620 ± 0.013	0.723 ± 0.014	0.557 ± 0.004	**0.771** ± **0.011**
PAD UFES 20	0.176	0.445	0.236	0.331	0.551 ± 0.015	0.613 ± 0.024	0.433 ± 0.017	**0.661** ± **0.015**
SD198	0.19	0.417	0.231	0.287	0.471 ± 0.021	0.554 ± 0.029	0.509 ± 0.019	**0.622** ± **0.012**
MSK	0.375	0.632	0.459	0.501	0.607 ± 0.015	0.740 ± 0.017	0.567 ± 0.004	**0.767** ± **0.012**
Milk10k clinic	0.224	0.559	0.297	0.401	0.559 ± 0.017	0.673 ± 0.023	0.432 ± 0.012	**0.707** ± **0.015**
Milk10k dermo	0.224	0.559	0.297	0.401	0.559 ± 0.017	0.673 ± 0.023	0.432 ± 0.012	**0.707** ± **0.015**

Performance is reported as average mAP ± standard deviation (ten repetitions) for image-to-text (upper part) and text-to-image (lower part) retrieval across datasets. Statistically significant tests cannot be performed, since state-of-the-art models are evaluated at fixed publicly available checkpoints. The method reaching the highest average is marked in bold. In case of equal values, the method reaching the lowest standard deviation is selected.

**Table 9:** Results on the zero-shot learning task for the MM architecture, comparing the performance of state-of-the-art foundation models (BioMedCLIP, MedImageInsights, MONET, Derm1M) with the performance reached with our framework, involving training the model with clinical notes synthesized prompting a LLM providing lesion metadata (L; GPT, MedGemma, SkinGPT4, DermLIP)

	State-of-the-art Foundation Models				Ours			
	BioMedClip	MedImageInsights	MONET	Derm1M	Gpt	MedGemma	SkinGPT4	DermLIP
*8-class*								
BCN20000	0.251	0.006	0.149	0.439	**0.738** ± **0.014**	0.718 ± 0.050	0.724 ± 0.045	0.691 ± 0.053
derm12345	0.506	0.000	0.426	0.763	0.811 ± 0.026	0.837 ± 0.016	0.840 ± 0.017	**0.845** ± **0.017**
Derm7pt	0.322	0.000	0.343	0.59	**0.744** ± **0.036**	0.664 ± 0.125	0.669 ± 0.072	0.670 ± 0.094
DermNet	0.307	0.011	0.386	0.491	0.671 ± 0.064	0.729 ± 0.103	**0.784** ± **0.080**	0.763 ± 0.077
FLUO_SC	0.238	0.192	0.294	0.573	0.743 ± 0.052	0.780 ± 0.061	**0.839** ± **0.033**	0.753 ± 0.082
MRA_MIDAS	0.189	0.01	0.177	0.252	0.468 ± 0.026	0.501 ± 0.021	0.474 ± 0.017	**0.501** ± **0.018**
HAM10000	0.406	0.000	0.341	0.698	0.734 ± 0.012	0.726 ± 0.018	**0.735** ± **0.015**	0.732 ± 0.015
SKINL2	0.428	0.000	0.491	0.543	0.772 ± 0.025	**0.771** ± **0.029**	0.740 ± 0.051	0.744 ± 0.023
Fitzpatrick17k	0.395	0.000	0.287	0.410	0.516 ± 0.050	0.533 ± 0.048	0.525 ± 0.051	**0.545** ± **0.038**
HIBA	0.196	0.003	0.240	**0.512**	0.464 ± 0.073	0.503 ± 0.041	0.499 ± 0.065	0.496 ± 0.031
PAD UFES 20	0.35	0.153	0.295	**0.624**	0.523 ± 0.027	0.542 ± 0.017	0.567 ± 0.019	0.570 ± 0.018
SD198	0.327	0.118	0.404	**0.472**	0.367 ± 0.042	0.353 ± 0.036	0.384 ± 0.040	0.429 ± 0.027
MSK	0.353	0.004	0.186	0.523	0.570 ± 0.015	0.550 ± 0.025	**0.573** ± **0.024**	0.569 ± 0.022
Milk10k clinic	0.182	0.001	0.13	0.273	0.506 ± 0.027	**0.534** ± **0.025**	0.510 ± 0.026	0.502 ± 0.023
Milk10k dermo	0.174	0.001	0.089	0.269	**0.642** ± **0.010**	0.627 ± 0.013	0.613 ± 0.016	0.618 ± 0.009
*15-class*								
BCN20000	0.154	0.006	0.155	0.037	0.386 ± 0.015	0.419 ± 0.107	0.511 ± 0.087	**0.590** ± **0.009**
derm12345	0.309	0.000	0.100	0.335	0.450 ± 0.007	0.440 ± 0.056	0.156 ± 0.045	**0.464** ± **0.103**
Derm7pt	0.135	0.000	0.226	0.383	0.653 ± 0.047	0.395 ± 0.151	**0.679** ± **0.020**	0.402 ± 0.116
DermNet	0.323	0.011	0.313	0.488	0.570 ± 0.053	0.537 ± 0.106	**0.739** ± **0.062**	0.642 ± 0.046
FLUO_SC	0.346	0.192	0.224	0.244	0.436 ± 0.095	0.282 ± 0.104	0.650 ± 0.123	**0.727** ± **0.061**
MRA_MIDAS	0.108	0.010	0.124	0.096	0.233 ± 0.014	0.256 ± 0.068	0.314 ± 0.043	**0.391** ± **0.033**
HAM10000	0.261	0.000	0.049	0.018	0.156 ± 0.009	0.341 ± 0.141	0.279 ± 0.134	**0.554** ± **0.121**
SKINL2	0.21	0.000	0.347	0.318	0.373 ± 0.016	0.426 ± 0.088	0.425 ± 0.057	**0.525** ± **0.067**
Fitzpatrick17k	0.22	0.000	0.247	0.340	0.215 ± 0.019	0.274 ± 0.086	**0.446** ± **0.013**	0.421 ± 0.021
HIBA	0.109	0.003	0.098	0.142	0.207 ± 0.032	0.208 ± 0.094	0.300 ± 0.029	**0.301** ± **0.087**
PAD UFES 20	0.384	0.153	0.267	0.276	0.336 ± 0.048	0.281 ± 0.053	0.439 ± 0.066	**0.541** ± **0.019**
SD198	0.166	0.048	**0.385**	0.458	0.275 ± 0.021	0.237 ± 0.033	0.268 ± 0.035	0.285 ± 0.018
MSK	0.125	0.004	0.128	0.193	**0.313** ± **0.012**	0.235 ± 0.029	0.253 ± 0.009	0.254 ± 0.055
Milk10k clinic	0.211	0.001	0.101	0.105	0.374 ± 0.027	0.300 ± 0.062	0.368 ± 0.036	**0.422** ± **0.029**
Milk10k dermo	0.156	0.001	0.106	0.050	0.462 ± 0.009	0.379 ± 0.083	0.478 ± 0.032	**0.516** ± **0.011**

Performance is reported as average F1-score ± standard deviation (ten repetitions) for 8-classes (upper part) and 15-classes (lower part) setups across datasets. Statistically significant tests cannot be performed, since state-of-the-art models are evaluated at fixed publicly available checkpoints. The method reaching the highest average is marked in bold. In case of equal values, the method reaching the lowest standard deviation is selected.

The results on cross-modal retrieval, for both image-to-text and text-to-image retrieval tasks, show that our MM architecture, trained with L, achieves higher average F1-scores than large-scale foundation models such as BioMedCLIP, MedImageInsights, MONET, and Derm1M. The performance improvement is in the range of 0.10–0.30 in terms of mAP. While foundation models exhibit moderate performance on some datasets, they often underperform on datasets including dermoscopic images. On the other hand, the MM architecture, particularly when trained on notes generated using DermLIP and MedGemma, reach consistently high performance across both dermoscopic and clinical image domains.

The results on zero-shot learning show that the MM architecture reaches higher performance than state-of-the-art foundation models in both zero-shot learning setups, confirming its superior generalization capabilities to generalize on new data, especially when including dermoscopic images. Foundation models generally reach limited or unstable performance, particularly in the 15-class setting, where average F1-scores often drop below 0.3 and, in several cases, is close to zero (e.g., MedImageInsights outputs). On the contrary, the MM architecture trained with LLM-synthesized clinical notes achieve consistently higher F1-scores across all datasets and class granularity setups. The performance gap is especially pronounced on datasets including dermoscopic images, where MM models outperform foundation models by large margins, with absolute differences in F1-score ranging from 0.20 to 0.40. Despite reaching lower performance in the 15-classes setup, the relative performance drop from 8-class to 15-class setups is significantly smaller for MM architectures compared to state-of-the-art foundation models, suggesting improved scalability to identify finer-grained diagnostic lesion types.

### Generated clinical notes as a tool for telemedicine

The alignment across modalities learnt by the MM architecture can be potentially exploited to generate clinical notes from input skin lesion images.

This section compares the MM representations learnt with L strategy (i.e., training the architecture with clinical notes synthesized by LLM, prompted with metadata) across different LLMs. For each MM architecture, the image representation feeds the new auxiliary text decoder, which outputs a textual description of the image. The generated clinical notes are evaluated on two aspects: the capability of the model to identify the right lesion and how similar the new clinical note is to the one synthesized by the LLM.

Table 10 summarizes the capability of the MM architecture to identify the right lesion, reporting the average number of clinical notes including the right skin lesion. As shown in previous results, the MM architectures trained with clinical notes generated by SkinGPT4 and DermLIP reach the lowest performance, missing around 20–40% of right classes and showing a larger standard deviation, symptom of a larger variability in predictions. On the other hand, the architecture trained on GPT and MedGemma notes (the one generally achieving the highest performance) mislabel a smaller number of samples. The reason can be identified in the notes generated by SkinGPT4 and DermLIP, usually noisier and shorter, while GPT and MedGemma allow to input and output more tokens, which guarantees more stability and reduces noise.

**Table 10:** Overview of the capability of the textual decoder to identify the right lesion

	L strategy			
	GPT	MedGemma	SkinGPT4	DermLIP
BCN20000	0.845 ± 0.021	**0.859** ± **0.033**	0.771 ± 0.044	0.602 ± 0.066
derm12345	**0.936** ± **0.007**	0.922 ± 0.006	0.650 ± 0.247	0.464 ± 0.249
Derm7pt	0.844 ± 0.025	**0.888** ± **0.029**	0.736 ± 0.041	0.712 ± 0.023
DermNet	0.798 ± 0.022	**0.822** ± **0.033**	0.698 ± 0.109	0.679 ± 0.021
FLUO_SC	0.826 ± 0.020	**0.834** ± **0.014**	0.690 ± 0.100	0.511 ± 0.139
MRA_MIDAS	0.685 ± 0.026	**0.702** ± **0.038**	0.613 ± 0.043	0.481 ± 0.069
HAM10000	**0.787** ± **0.006**	0.787 ± 0.007	0.511 ± 0.186	0.360 ± 0.163
SKINL2	0.784 ± 0.008	**0.815** ± **0.017**	0.593 ± 0.114	0.519 ± 0.123
Fitzpatrick17k	**0.503** ± **0.019**	0.475 ± 0.070	0.359 ± 0.017	0.384 ± 0.042
HIBA	**0.521** ± **0.046**	0.513 ± 0.014	0.388 ± 0.091	0.275 ± 0.046
PAD UFES 20	0.561 ± 0.012	**0.568** ± **0.019**	0.485 ± 0.072	0.382 ± 0.053
SD198	0.400 ± 0.054	**0.412** ± **0.023**	0.263 ± 0.050	0.271 ± 0.024
MSK	**0.634** ± **0.012**	0.619 ± 0.007	0.496 ± 0.076	0.376 ± 0.073
Milk10k clinic	**0.532** ± **0.035**	0.528 ± 0.009	0.342 ± 0.071	0.387 ± 0.075
Milk10k dermo	**0.641** ± **0.021**	0.627 ± 0.027	0.562 ± 0.029	0.524 ± 0.012

The model is fed with the image representations learnt with strategy L, considering each LLM to synthesize data. For each dataset, the number of correct skin lesions are reported as average and standard deviation of the five experiment accuracies. The method reaching the highest average is marked in bold. In case multiple methods reach the same value, the best model is selected considering the lowest standard deviation.

A similar trend is experienced when analyzing qualitatively the clinical notes. Table 11 qualitatively analyzes the notes synthesized with the text decoder. Considering the MM architecture trained using clinical notes synthesized with LLM L, for each input image ${X}_{1}$, the clinical note ${C}_{1}$ generated using the LLM L is used as reference note. The same image ${X}_{1}$ is then processed by our text decoder to produce a clinical note ${T}_{1}$. We compute BERTScore^52^ by comparing ${T}_{1}$ against ${C}_{1}$, measuring the semantic similarity between the output of our decoder and the note generated by the LLM under the same input conditions. In the case of GPT and MedGemma, two reference notes (i.e., L1 and L2) are used, corresponding to the two types of notes used during training. SkinGPT4 and DermLIP are trained with a single type of note due to the limitations on prompt flexibility. Section Clinical note generation structure (Supplementary Materials) describes the prompt structure to generate notes. Results show a slightly higher score obtained by GPT and MedGemma, which generally outperform the other architectures in terms of F1-BertScore.

**Table 11:** Overview of the qualitative performance of the textual decoder to synthesize clinical notes from the images

	L1 – GPT	L2 – GPT	L1 – MedGemma	L2 – MedGemma	L – SkinGPT4	L – DermLIP
BCN20000	0.943 ± 0.028	0.933 ± 0.018	0.935 ± 0.015	**0.959** ± **0.018**	0.922 ± 0.000	0.913 ± 0.001
derm12345	0.924 ± 0.020	0.937 ± 0.011	0.940 ± 0.017	**0.947** ± **0.019**	0.921 ± 0.000	0.905 ± 0.002
Derm7pt	0.919 ± 0.016	0.937 ± 0.012	0.915 ± 0.005	**0.937** ± **0.005**	0.920 ± 0.001	0.897 ± 0.003
DermNet	0.910 ± 0.003	**0.955** ± **0.002**	0.912 ± 0.009	0.941 ± 0.007	0.917 ± 0.001	0.921 ± 0.002
FLUO_SC	0.912 ± 0.006	**0.954** ± **0.003**	0.921 ± 0.009	0.938 ± 0.007	0.917 ± 0.001	0.931 ± 0.003
MRA_MIDAS	0.926 ± 0.017	0.930 ± 0.010	0.922 ± 0.014	**0.931** ± **0.011**	0.915 ± 0.001	0.905 ± 0.001
HAM10000	0.935 ± 0.026	0.937 ± 0.016	0.937 ± 0.019	**0.950** ± **0.020**	0.924 ± 0.002	0.910 ± 0.005
SKINL2	0.926 ± 0.017	0.938 ± 0.008	0.927 ± 0.014	**0.931** ± **0.010**	0.920 ± 0.001	0.898 ± 0.003
Fitzpatrick17k	0.917 ± 0.013	**0.940** ± **0.008**	0.916 ± 0.009	0.924 ± 0.008	0.919 ± 0.001	0.917 ± 0.004
HIBA	0.913 ± 0.007	**0.943** ± **0.004**	0.921 ± 0.010	0.931 ± 0.009	0.918 ± 0.001	0.905 ± 0.002
PAD UFES 20	0.923 ± 0.012	**0.938** ± **0.008**	0.922 ± 0.010	0.936 ± 0.008	0.918 ± 0.001	0.932 ± 0.003
SD198	0.911 ± 0.007	**0.939** ± **0.006**	0.910 ± 0.011	0.925 ± 0.006	0.917 ± 0.001	0.915 ± 0.002
MSK	0.930 ± 0.021	0.930 ± 0.013	0.931 ± 0.014	**0.940** ± **0.013**	0.919 ± 0.000	0.900 ± 0.002
Milk10k (C)	0.920 ± 0.014	**0.937** ± **0.009**	0.925 ± 0.013	0.935 ± 0.009	0.919 ± 0.001	0.935 ± 0.004
Milk10k (D)	0.941 ± 0.026	0.927 ± 0.017	0.933 ± 0.013	**0.953** ± **0.017**	0.919 ± 0.000	0.917 ± 0.001

The performance is evaluated comparing the description generated by the decoder and the reference note reported in the column header. For each dataset, average ± standard deviation F1-BertScore is reported. Since GPT and MedGemma are queried with two different types of prompts, L strategy is reported as L1 and L2, respectively. The best result (highest F1-BertScore) is highlighted in bold. In case multiple methods reach the same value, the best model is selected considering the lowest standard deviation.

Clinical notes generated using images as input can be potentially used to deploy applications supporting telemedicine, providing a tool helpful for both dermatologists and patients. “Discussion” section describes all the intermediate steps needed to validate the tool.

## Discussion

The results show that existing LLMs can be used to synthesize valuable clinical notes of dermatology images, allowing the development of MM algorithms even when publicly available datasets are unimodal and limited in size.

LLMs can be prompted using metadata linked to the original images, which usually include heterogeneous information about the lesion, generating reliable clinical notes that can be paired with images. Their use leads to train MM architectures that achieve robust performance on both cross-modal retrieval and zero-shot learning tasks. Importantly, prompting LLMs with metadata results in better performance than either using metadata alone to fill a fixed note template (M) or prompting an LLM without any lesion-specific information (OL). Furthermore, using metadata to enrich prompts to generate a clinical note (L) leads to higher performance on downstream tasks than using metadata to fill a fixed clinical note template (M). The consistent and substantial outperformance of across datasets cannot be attributed to label access alone. Instead, it reflects the richer semantic content provided by the LLM, such as the descriptive detail about visual appearance and morphology that goes beyond a class label, and demonstrates that multimodal alignment benefits from expressive natural language descriptions rather than from structured label encoding. This finding shifts the perspective on the role of LLMs in dermatology, while prior literature mainly treated LLMs as standalone tools, our results highlight their value to pair images with meaningful clinical notes to support MM learning and foundation models.

The possibility of creating multimodal datasets from unimodal data, however, comes with an important trade-off related to the content of the generated descriptions. While lesion type information is reliably provided by metadata, LLMs struggle to provide accurate information about other dermatology-specific features. The analysis conducted on the PH2 dataset shows that LLMs are more effective at describing general-purpose concepts, such as lesion asymmetry or border regularity. Counterintuitively, LLMs specialized in dermatology tend to output more hallucinated content than general-purpose (GPT) or general biomedical (MedGemma) models. A key difference between these groups lies in input and output flexibility. GPT and MedGemma are designed to process longer and more flexible inputs, allowing users to explicitly query specific characteristics, using additional tokens to provide details. In contrast, SkinGPT4 and DermLIP are trained with a limited set of prompts, which reduces output variability and limits the amount of information that can be provided as input. This result is not trivial, as it highlights that the design of the input query is a fundamental element to obtain reliable outputs from LLMs, even when the model is trained on limited datasets or is specialized for a specific domain.

Building on these observations, the results achieved in this paper show that, with appropriate setups and training strategies, a relatively small MM architecture can outperform larger foundation models trained on datasets of significantly greater magnitude. The comparison with Derm1M clearly illustrates this aspect. Despite being trained with only 16,000 samples, two orders of magnitude fewer than Derm1M, our architecture achieves higher performance considering most datasets, for the two downstream tasks. The limited number of training samples is compensated by the quality of the data, which primarily includes dermoscopic images with lesions represented in the foreground and therefore allows the model to focus on clinically relevant visual content. On the other hand, the compared foundation models are trained on larger collections, but mostly including noisy samples, with the images including lesions acquired with heterogeneous protocols and the texts not focusing on the specific characteristic of the lesion. This suggests that carefully curated datasets can be effectively exploited to achieve equal or even higher performance compared to large-scale but less controlled data collections. Therefore, the increasing availability of public dermatology datasets may foster a virtuous cycle, where existing resources are progressively leveraged to create larger and more heterogeneous multimodal datasets.

Although the training set includes samples from six data sources and is primarily composed of dermoscopic images, the architecture reaches robust performance on external datasets, also considering clinical images, inherently noisier, collected from different sources. This result indicates that the model is not biased toward the specific characteristics of training data, while instead it can effectively adapt to heterogeneous inputs. Consequently, the generalization observed on external data suggests that the model can effectively handle the data variability expected in clinical practice. This generalization can be explained by the role of textual content in the learning process. While images are strongly influenced by acquisition procedures and device-specific characteristics, clinical notes are more standardized and encode a shared semantic space. Text therefore acts as a bridge that links visual concepts, expressed by different pixel distributions across datasets, to a limited and consistent set of dermatological concepts and terminology. This regularizing effect mitigates overfitting and improves robustness to domain shift. Conversely, when textual supervision is limited, as in the M strategy that focuses only on lesion type, generalization performance is reduced. This behavior is further supported by the role of prompt-based strategies. When multiple lesion descriptions are used during training, the model achieves robust performance across several external datasets. This suggests that combining multiple sources of information helps to build stronger clinical knowledge. This knowledge can be transferred to new data without a significant drop in performance. This observation is consistent with clinical practice, where medical experts rely on diverse sources of experience when making decisions. Therefore, the results suggest that generalization may not rely exclusively on the visual heterogeneity of training images. Instead, it may also benefit from the inclusion of clinical information encoded in clinical notes. Combining multiple lesion descriptions can be interpreted as integrating complementary clinical perspectives, which may support more robust decision-making, in a way that is consistent with how medical experts leverage diverse sources of knowledge in clinical practice.

Finally, the results obtained across the proposed tasks indicate that the MM architecture has potential applications in telemedicine and clinical documentation support. For example, the architecture could in principle be extended with a textual decoder to support the drafting of clinical notes from images, reducing documentation workload in telemedicine settings where dermatologist availability is limited as described in the section “Generated clinical notes as a tool for telemedicine”, in line with recent LLM-based applications in dermatology, such as the interactive use presented for SkinGPT4^2^. At the same time, LLM-synthesized notes are inherently noisy and may include inaccurate information, highlighting the risks of deploying such systems without appropriate safeguards. From a clinical perspective, these findings emphasize that while MM systems can improve efficiency and access to care, their deployment must be guided by rigorous validation. The present study does not include clinical validation against real clinical notes, reader studies, prospective evaluation, workflow integration testing, or safety analysis. Clinical deployment would require all of these validation steps. Generated notes should be treated as assistive outputs within human-in-the-loop workflows, not as autonomous clinical documents, and their use must be accompanied by appropriate uncertainty communication and clinical oversight.

## Methods

### Multimodal architecture

The MM architecture is designed combining multiple components to analyze and align visual and textual dermatology data in a multimodal representation^53,54^, adopted to perform multiple downstream tasks.

**Fig. 2:**
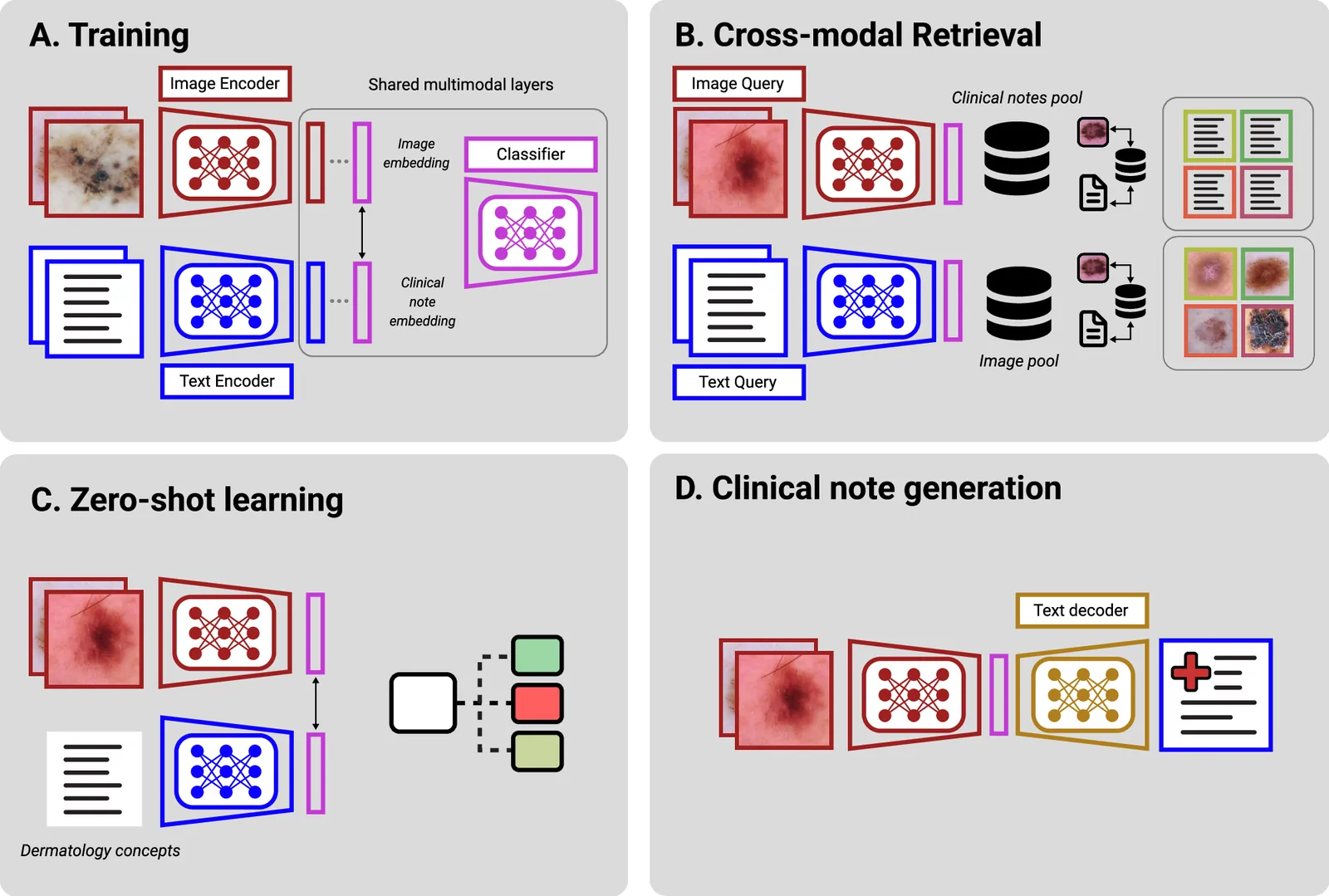
Overview of the architecture, including the training schema and tasks performed by the MM architecture. **A** Training. The training schema of the multimodal architecture, including two input branches for images (red) and text (blue). The encoders process different modality and produce fixed-size embeddings, which are aligned before feeding shared layers (magenta). The alignment involves a loss function including three terms: L1-loss, cosine similarity loss, NT-Xent. The shared layers (inside a box) include also a classifier, which outputs predictions for both modalities. **B** Cross-modal retrieval. The cross-modal retrieval involves single modality embeddings: the input sample (image or clinical note) embedding is compared with a pool of embeddings from the other modality. The embeddings from the pool are ranked according to their cosine similarity with the input, selecting the ones with the highest values. A retrieved sample is considered correct if it shares the class with the input sample, incorrect otherwise. **C** Zero-shot learning. The image embeddings are compared with the embeddings of specific textual dermatology concepts, that can be classes or keywords. The image is classified according to the concept reaching the highest cosine similarity. **D** Clinical note generation. An image embedding feeds a text decoder (light brown), which outputs a text including the image description.

Figure 2 illustrates the overall architecture structure, which includes two independent branches responsible for modality-specific processing and a shared block of layers where information from images and text is combined into a common latent space. The individual encoders are modular and can be readily substituted with alternative architectures. The image-encoder is the PanDerm^4^ image-encoder (red in Fig. 2), a large vision-transformer (ViT) architecture trained on dermatology data including 85 M parameters. The encoder is fed with 224 × 224 × 3 RGB images processed by 24 transformer blocks. The ViT parameters are not frozen during training, and the encoder is fully fine-tuned, despite containing a large number of parameters compared to the reduced number of the training samples. The textual encoder is a PubMedBERT^55,56^ encoder (blue in Fig. 2), a BERT-based large transformer architecture trained on medical texts. It is fed with textual tokens, 512 tokens as input, processed by 12 transformer blocks. The text-encoder includes a large number of parameters, around 110 M, but in contrast to the image-encoder, its transformer block parameters are frozen during training. The choices related to parameter freezing aims to reduce the risk of multimodal representation overfitting. Both input branches include an additional component introduced to project the resulting embeddings into a fixed dimension of 128. This embedding projection is functional to feed modality-specific representations into the shared block of the architecture. The shared layers consist of two fully connected layers, with the first operating on the 128-dimensional embeddings and the second acting as a classifier for the eight classes. The inclusion of a classification layer aims to implicitly regularize the learned embeddings and limit possible overfitting effects. The risk of overfitting remains a challenge due to the limited dataset size, the large number of parameters in the two encoders, and the simultaneous optimization of a loss function including three terms, that typically require larger data. However, it is mitigated through several complementary mechanisms. PanDerm is pre-trained on over 2 million dermatology images, so that the fine-tuning on 16,000 samples constitutes domain adaptation of an already well-calibrated representation space, not training from scratch. The PubMedBERT text encoder is frozen throughout training, substantially reducing trainable parameters. The classifier head acts as a regularizer: Section Ablation Study: Classifier (Supplementary Materials) shows two Tables (Supplementary Tables 3 and 4) reporting how performance decreases (10-30 points in both mAP and F1-score) when the architecture is trained without the optimization of the classifier.

During training, the shared layers are fed with embeddings from both modalities in an alternating manner rather than simultaneously. This choice allows the model to learn features that are discriminative for both modalities while encouraging the emergence of a common latent space that shares information across visual and textual inputs. Performance of the classifier is reported in Supplementary Table 5 and Supplementary Table 6 (Section Classification Results, Supplementary Materials).

Despite the modalities are processed separately, the output embeddings from the projection layers (both 128 in size) are aligned using a loss function including three terms, to explicitly enforce modality alignment in the shared latent space and to prevent embedding space collapse. These loss functions include L1 loss, a cosine similarity loss, and a modified variant of the NT-Xent (Normalized Temperature-scaled Cross Entropy) loss^57^, which is described in more detail in Section Loss function to align representation, Supplementary Materials. The combination of these losses promotes consistency between image and text representations and further contributes to regularization during training.

The aligned representations learned before the classifier enable the architecture to perform multiple tasks and facilitate the integration of additional lightweight networks for task-specific objectives. Figure 2B and Fig. 2C illustrate how cross-modal retrieval and zero-shot learning tasks are performed using the shared representations. Figure 2D shows how the note generation module (brown) operates as a decoder that receives image embeddings and outputs a clinical note, demonstrating the flexibility of the learned multimodal representation across heterogeneous downstream tasks. Further details regarding the architectural design and choices are described in another publication^58^.

### Downstream tasks

The MM architecture performance is evaluated considering three tasks: cross-modal retrieval, zero-shot learning, clinical note generation.

The cross-modal retrieval evaluates the ability of the system to retrieve relevant samples, based on a query from the opposite modality. Embeddings are extracted from the shared latent layer immediately before the classifier and cosine similarity is used to rank candidates, with higher cosine denotes greater similarity. Retrieval is evaluated in two directions: image-to-text, where the query is an image and the model ranks candidate notes; and text-to-image, where the query is a clinical note report and the model ranks candidate images. In the image-to-text scenario, the clinical note pool contains all the notes generated with M strategy. In the text-to-image scenario, multiple input queries involve notes generated with the M strategy. A retrieved sample is considered correct if it shares the class with the input sample, incorrect otherwise. This class-level criterion is adopted as an evaluation proxy for multimodal alignment quality, applied consistently across all 15 datasets. Fine-grained morphological retrieval criteria, based for example on border irregularity, pigmentation pattern, or dermoscopic structure, are not available across most of the datasets used and would prevent a consistent evaluation. The performance is assessed using micro-average precision (mAP), which treats retrieval results from all queries as a single set of predictions and computes precision across those predictions to provide a global measure of retrieval quality.

The zero-shot learning task evaluates the ability of the system to classify classes or concepts without using the trained classifier, by linking image embeddings directly to textual concept embeddings. Image embeddings, taken from the shared latent layer, are compared by cosine similarity to embeddings of candidate keywords; a concept is assigned to an image when it obtains the highest cosine similarity. In both setups, the trained classifier head is not used. We evaluate two setups: 8-classes setup that uses the eight diagnostic categories, and 15-classes setup that uses a predefined list of dermatology-related keywords. The 8-class setup involves the following concepts: ‘actinic keratosis’, ‘basal cell cancer’, ‘benign/seborrheic keratosis’, ‘dermatofibroma’, ‘melanocytic/benign nevus’, ‘melanoma’, ‘squamous cell cancer’, ‘vascular lesion’. The 15-class setup involves a broader set of 15 concepts, including: ‘actinic keratosis’, ‘basal cell carcinoma’, ‘benign melanocytic nevus’, ‘blue nevus’, ‘bowen disease/SCC in situ’, ‘congenital/special-pattern nevi’, ‘dermatofibroma/fibrous lesions’, ‘dysplastic/atypical nevus (clark-type)’, ‘lentigo maligna’, ‘lichenoid keratosis’, ‘melanoma’, ‘seborrheic keratosis & pigmented keratoses solar lentigo’, ‘squamous cell carcinoma (invasive)’, ‘vascular lesions’. The latter case evaluates semantic generalization to unseen dermatological concepts. Performance is reported with the weighted F1-score, emphasizing the model ability to generalize on classes or concepts not explicitly learned by the classifier. When assessing the performance of SOTA foundation models (see the section “MM architecture reaches higher performance than state-of-the-art dermatology foundation models”), the zero-shot learning task is evaluated using model specific interface provided to perform the task, described in the corresponding repository.

The clinical note generation task evaluates the ability of the system to produce a textual description of an input image. To perform this task, a supplementary lightweight decoder is trained, separately from the classifier-based objectives. The decoder is fed with an image embedding from the shared latent space as input and autoregressively generates a clinical note. The goal of this task is to evaluate two aspects: the textual style and the content of the note. The first aspect is evaluated using BERTScore, which compares contextual token embeddings with a reference input, the clinical note generated by the LLM capturing semantic and terminological consistency. The second aspect is evaluated with weighted F1-score, analyzing whether the correct lesion type (i.e., the primary hallucination risk for which ground truth is available) is present in the generated note. Both metrics serve as automated proxies for training-relevant note quality rather than clinical quality assessments. BERTScore is insensitive to factual clinical errors, while the weighted F1-score does not detect other incorrect outputs related to the lesion characteristics. Expert clinician review would be required for clinical accuracy assessment, which is not feasible at the scale of this study and is acknowledged in the Limitations section.

### Benchmark LLM and foundation models

The paper compares synthetic notes generated by four LLMs and the results of the MM architecture with four foundation models.

Four LLMs are used to synthesize clinical notes: GPT-4o-mini, MedGemma^59^, SkinGPT-4^2^, DermLIP^60^.

GPT-4o-mini^61^ is a compact and efficient, transformer-based large language model obtained from the GPT-4 family, including a decoder-only architecture optimized for efficiency. It is an autoregressive language model, generating token-by-token text, based on contextual probability modeling. Training involves large-scale self-supervision on diverse text corpora. Afterwards, supervised instruction tuning and reinforcement learning are performed with human feedbacks, to improve reliability and adherence to task instructions. When coupled with an external image encoder or multimodal interface, the model can transform dermatological image interpretations into coherent clinical descriptions or summaries.

MedGemma-4B-IT (medgemma-4b-it) is a multimodal medical instruction-tuned model, composed of a vision encoder coupled with a decoder-style transformer language model, containing approximately four billion parameters. The image encoder is based on a SigLIP-style vision transformer^62^, which converts medical images into visual embeddings while preserving spatial and semantic information. The text component is a Gemma-family language model trained to generate medical text conditioned on both visual and textual inputs. Training follows a two-stage process. The first stage involves a large-scale pretraining on heterogeneous medical data, including 33 M pairs de-identified medical images, such as dermatology, radiology, and pathology, paired with clinical text. The second stage involves instruction tuning, using curated medical question-answer and image-text supervision. The alignment allows the model to generate clinically relevant explanations, summaries, and reports from medical images.

SkinGPT-4 is a dermatology-focused multimodal system, integrating a pre-trained vision transformer with a large language model to generate clinically meaningful interpretations of skin images. The image encoder is a transformer-based^63^ visual backbone (e.g., ViT^64^ or CLIP-derived architecture^65^) trained to extract features from dermatological images. The textual component is a large autoregressive language model, commonly based on LLaMA-family architectures^66^, fine-tuned for medical dialogue and explanation. The model is trained using paired dermatology images and corresponding expert-annotated clinical descriptions, diagnoses, or educational text. The training dataset includes around 30,000 images collected from a private repository and 18,000 publicly available images collected from SKINCON and DermNet. The alignment between image embeddings and text representations allows the system to generate structured clinical narratives and diagnostic reasoning conditioned on visual input. The LLM is trained on a limited set of prompts, limiting the structures and content of prompts to query the LLM.

DermLIP-GPT2-Captioner is a domain-specific image captioning model designed for dermatology images. The image encoder is a Vision Transformer (ViT-B/16) trained on the Derm1M dataset^3^, optimized to capture dermatological visual features such as lesion morphology, color, and texture. The textual encoder/decoder is GPT-2, an autoregressive transformer language model adapted for short clinical text generation. GPT-2 is outdated, therefore it is only possible to provide a limited context for prompts, limiting the possibility to exploit long prompts. Training includes an initial general vision-language alignment phase followed by dermatology-specific fine-tuning using curated image-caption pairs describing skin conditions. Visual embeddings generated by the image encoder are projected into the language model input space to condition text generation.

Four foundation models are adopted as benchmark to contextualize the performance of the MM architecture with state-of-the-art algorithms: BioMedCLIP^34^, MedImageInsights^50^, MONET^51^, Derm1M^64^.

BioMedCLIP is a biomedical vision-language foundation model trained to align images and text within a shared embedding space using contrastive learning. The image encoder is a Vision Transformer (ViT) trained to extract high-level visual representations from biomedical images. The text encoder is PubMedBERT^56^, a transformer language model pre-trained on biomedical literature. The model is trained on a large corpus of image-text pairs extracted from scientific articles in PubMed Central, enabling robust alignment between visual data and domain-specific terminology. BioMedCLIP can be adopted for zero-shot image classification, cross-modal retrieval, rather than text generation.

MedImageInsights is a medical imaging foundation model that generates compact embeddings for images and associated clinical text using a dual-encoder transformer architecture. A ViT extracts visual features from diverse medical images including multiple modalities, while a separate transformer-based text encoder represents diagnostic labels and clinical descriptors. The model is trained using contrastive learning on large collections of paired medical images and text (3.7 M images), aligning both modalities in a shared embedding space. This design enables zero-shot classification, image retrieval, and similarity-based analysis without task-specific retraining.

MONET is a dermatology-focused vision-language model designed for skin images and clinical concepts. The image encoder is a CLIP ViT-L/14 trained on dermatology images, while the text encoder is a transformer-based language model trained on dermatology-related medical text. The model is trained using contrastive learning on paired image-text data (105,550 couples collected from PubMed and textbooks), forcing alignment between visual patterns and clinically meaningful descriptors. MONET is primarily used for concept retrieval, image annotation, and evaluation of dermatology-specific representation learning in research settings.

Derm1M (DermLIP PanDerm-base-w-PubMed-256) is a dermatology-focused vision–language foundation model built following a CLIP-style dual-encoder architecture. The image encoder is a Vision Transformer (ViT-B/16) optimized to capture fine-grained dermatological visual patterns such as lesion morphology, color variation, and texture, while the text encoder is PubMedBERT^56^, a transformer language model pre-trained on biomedical literature to represent clinical terminology and disease concepts. Both encoders are trained jointly using contrastive learning so that corresponding dermatology images and clinical notes are aligned in a shared embedding space to enable cross-modal retrieval and zero-shot inference. The model is designed as a foundation for tasks such as image-text retrieval, concept recognition, and zero-shot skin disease classification. The model is trained on Derm1M^3^, a large-scale dermatology vision–language dataset containing over one million image–text pairs covering hundreds of skin conditions. Derm1M aggregates images from diverse dermatology sources (textbooks, internet, videos and publicly available datasets) and pairs them with clinically grounded textual descriptions derived from medical literature and educational materials.

### Dataset description

The dataset contains dermatologic images (paired with a synthesized clinical note) collected from publicly available medical repositories.

**Fig. 3:**
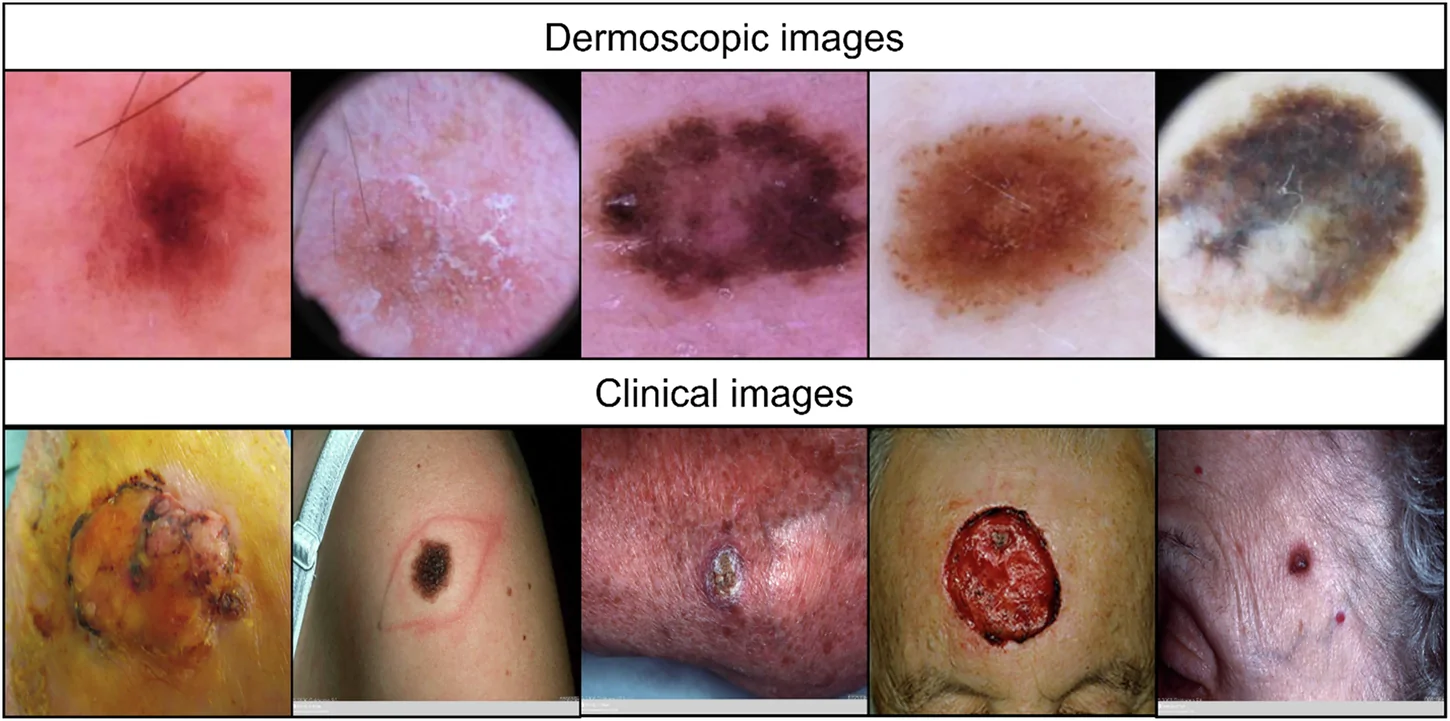
Representative comparison of dermoscopic and clinical images. The upper row shows dermoscopic images, and the lower row shows corresponding clinical images. Dermoscopic images (selected from BCN2000, SKINL2, HAM20000) are acquired under controlled conditions with magnification and specialized lighting, resulting in centered lesions with clearly visible diagnostic structures (e.g., pigment networks, globules, and vascular patterns). In contrast, clinical images (selected from Fitzpatrick17k, SD198, PAD-UFES-20) are standard photographs captured without magnification, often containing background skin, variable illumination, shadows and off-center lesions.

The adoption of images collected from multiple sources guarantees broad variability in visual appearance. The visual content exhibits variability in visual appearance arising from differences in lighting, magnification, anatomical site, skin pigmentation, and lesion structure. To capture such variability, the dataset includes two types of images: dermoscopic and clinical images. Dermoscopic images are captured in a controlled setting, centered on the lesion with relatively less noise. They represent high-resolution, magnified views obtained under polarized or non-polarized specific devices. On the other hand, clinical images provide wider, non-magnified context photographs where lesions may not be centered. Figure 3 presents representative examples of both dermoscopic and clinical images.

The dataset is split into training, validation, and testing partitions, which are presented in Table 12. Both dermoscopic and clinical images are present in the training and validation partitions, allowing the model to learn from heterogeneous visual input and preventing overfitting to a single imaging type. The test partition includes both internal data, which are independent samples collected from the same sources used for training and validating the architecture, and external data, which are samples collected from independent repositories. This configuration allows quantitative assessment of performance under both in-distribution and out-of-distribution.

**Table 12:** Overview of the dataset composition

Dataset	BEK	DF	NEV	VASC	ACK	BCC	MEL	SCC
*Training partition*								
BCN20000^42^ (D)	796	86	2944	77	515	1966	1999	391
Derm12345^13^ (D)	456	128	2116	214	56	300	271	241
Derm7pt^43^ (C)	46	14	18	17	0	26	160	0
DermNet^67^ (C)	154	40	39	0	38	133	62	21
FLUO_SC^68^ (C)	176	0	110	0	392	347	13	54
MIDAS^69^ (C)	215	39	555	26	135	419	164	259
TOTAL training	1843	307	5782	334	1136	3191	2669	966
*Validation partition*								
BCN20000 (D)	170	18	630	16	110	421	428	57
Derm12345 (D)	75	12	891	17	4	57	37	15
Derm7pt (C)	21	6	8	8	0	12	69	0
DermNet (C)	22	7	7	0	6	19	10	7
FLUO_SC (C)	26	0	17	0	56	50	3	9
MIDAS (C)	32	7	80	5	20	61	24	37
TOTAL validation	346	50	1633	46	196	620	571	125
Internal Testing partition								
BCN20000 (D)	172	20	632	18	112	422	430	111
Derm12345 (D)	175	40	1733	51	14	66	92	47
Derm7pt (C)	13	4	5	5	0	7	45	0
DermNet (C)	44	11	11	0	11	38	18	7
FLUO_SC (C)	50	0	31	0	112	99	3	15
MIDAS (C)	61	11	158	7	38	119	47	74
*External Testing Partition*								
HAM10000^21^ (D)	1338	160	7737	180	149	622	1305	229
SKINL2^44^ (D)	33	14	97	0	0	40	28	0
Fitzpatrick17k^45^ (C)	30	9	39	0	5	36	115	141
HIBA^46^ (D)	88	61	602	51	63	340	253	158
PAD UFES 20^47^ (C)	235	0	244	0	730	845	52	192
SD198^70^ (C)	60	18	32	3	62	13	38	0
MSK^71^ (D)	566	18	2595	67	251	741	1311	296
Milk10k^72^ (C)	544	52	746	47	303	2522	450	473
Milk10k^72^ (D)	544	52	746	47	303	2522	450	473
TOTAL Testing	3953	470	15,408	476	2153	8432	4637	2216

The dataset contains dermatology images paired with synthesized clinical notes, collected from multiple publicly available sources. Each sample is labeled with one of eight classes: BEK (Benign Keratosis), DF (Dermatofibroma), NEV (Benign Nevus), VASC (Vascular Lesion), ACK (Pre-cancer Actinic Keratosis), BCC (Basal-Cell Carcinoma), MEL (Melanoma), SCC (Squamous Cell Cancer). Images are either dermoscopic (D) or clinical (C). Data are split into three partitions: training, validation, testing. Training and validation partitions include four datasets, to exploit both dermoscopic and clinical samples. The testing partition includes ten datasets: the testing partitions of the four aforementioned datasets (internal partition) and six independent datasets (external partitions).

Samples used for training and validation are both dermoscopic (BCN20000^42^, Derm12345^13^) and clinical images (Derm7pt^43^, DermNet^67^, FLUO_SC^68^, MIDAS^69^). The test partition includes independent datasets not used for training, such as HAM10000^21^, SKINL2^44^, Fitzpatrick17k^45^, HIBA (Hospital Italiano Buenos Aires)^46^, PAD UFES 20^47^, SD198^70^, MSK^71^, Milk10k^72^ (the same lesions are acquired with both clinical and dermoscopic settings) in addition to the test subset of BCN20000, Derm12345, Derm7pt, DermNet, FLUO_SC, MIDAS. Partitions are strictly separated to prevent data leakage: a given image cannot appear in more than one partition, and where patient identifiers are available, all images from the same patient are assigned to a single partition. Separation was verified at image-ID level across all datasets and at patient-ID level where metadata permitted. Although several datasets in this work were contributed to the ISIC archive, they originate from distinct patient cohorts collected across different countries and institutions (Table 13) and do not share image.

**Table 13:** Overview of the dataset selection

Dataset	Origin	Selected samples	Total samples	Original classes	Mapping
BCN20000	Barcelona, Spain	12,514	12,514	9	Solar Lentigo -> BEK; Melanocytic Nevus -> NEV
Derm12345	Manisa, Türkiye and Istanbul, Türkiye	7108	12,346	40	Solar lentigo -> BEK; Lichenoid Keratosis -> BEK; Melanocytic Nevus -> NEV; Blue Nevus -> NEV; Congenital Nevus -> NEV; Bowen Disease -> ACK; Lentigo Maligna -> MEL
Derm7pt	San Francisco, California	484	1011	20	Solar Lentigo -> BEK; Blue Nevus -> NEV; Melanocytic Nevus -> NEV
DermNet	NZ Dermatological Society (online atlas)	705	1890	23	Melanocytic Nevus -> NEV
FLUO_SC	São Paulo, Brazil	1563	1563	6	–
MIDAS	Cleveland, Ohio	2593	3417	14	Benign-Seborrheic Keratosis -> BEK, Benign-Dermatofibroma -> DF, Other-Melanocytic Lesion with possible re-excision (severe/spitz nevus, aimp) -> NEV, Other-non-neoplastic/inflammatory/infectious -> NEV, Benign-Other -> NEV, Benign-Hemangioma -> VASC
HAM10000	Vienna, Austria, and Queensland, Australia	11,720	11,720	8	Melanocytic Nevus -> NEV
SKINL2	Leiria, Portugal	212	282	40	Lentigo Maligna -> MEL; Blue Nevus -> NEV; Melanocytic Nevus -> NEV; Atypical Nevus -> NEV
Fitzpatrick17k	Online atlases (DermaAmin, Atlas Dermatologico)	375	16,577	114	Lichen Planus -> BEK; Seborrheic Keratosis -> BEK; Nevocytic Nevus -> NEV; Halo Nevus -> NEV; Halo Nevus -> NEV; Melanocytic Nevus -> NEV
HIBA	Buenos Aires, Argentina	1616	1616	8	Lichen Planus -> BEK; Seborrheic Keratosis -> BEK; Solar Lentigo -> BEK
PAD UFES 20	Espírito Santo state, Brazil	2298	2298	6	–
SD198	Nankai, China	226	6584	198	Seborrheic Keratosis -> BEK; Bowen Disease -> BEK; Lichen Planus -> BEK; Solar Lentigo -> BEK; Halo Nevus -> NEV; Actinic Solar -> ACK
MSK	Memorial Sloan Kettering Cancer Center, New York	5845	5845	14	Seborrheic keratosis -> BEK, Solar Lentigo -> BEK, Lichen Planus like Keratosis -> BEK, Nevus -> NEV, Epidermal Nevus -> NEV, Atypical proliferative nodules in congenital Melanocytic Nevus -> NEV, Angiokeratoma -> VASC, Angiofibroma -> VASC, Melanoma invasive -> MEL, Melanoma- nos -> MEL, Melanoma in situ -> MEL, Melanoma metastasis -> MEL, Squamous Cell Carcinoma in situ -> SCC, Squamous Cell Carcinoma- nos -> SCC, Squamous Cell Carcinoma- invasive -> SCC, Baso-Squamous Carcinoma -> SCC
Milk10k	Multiple sources: Vienna, Austria; Ankara University, Ankara, Türkiye; Memorial Sloan Kettering Cancer Center, New York; Skopje, North Macedonia; Capalaba, Australia	5137	5137	11	–

The Table shows the number of samples collected from each original repository, the total classes in the original datasets, and how these classes were mapped to the final five target categories.

From all sources, only eight diagnostic categories are retained: Benign Keratosis (BEK, benign), Dermatofibroma (DF, benign), Benign Nevus (NEV, benign), Vascular Lesion (VASC, Benign), Actinic Keratosis (ACK, pre-cancerous), Basal-Cell Carcinoma (BCC, malignant), Melanoma (MEL, malignant), Squamous Cell Cancer (SCC, malignant). Furthermore, the PH2^73^ dataset is adopted to validate the capability of LLMs to synthesize clinical notes including valuable features related to the lesion. The dataset includes 200 images, annotated on specific lesion characteristics, such as the asymmetry/symmetry of the lesion, dermatology features, color of the lesion.

Both images and synthesized clinical notes are used during training to simultaneously learn visual and textual representations within a shared multimodal space, despite the training datasets includes primarily dermoscopic images. However, in clinical settings, interpreting the images correctly is more important than interpreting the notes, since the notes generally describe what a clinician already saw in the image. For this reason, the evaluation of textual content has limited clinical application, while the application of the MM on images has more clinical relevance.

### Data selection

The dataset adopted for the experiments is collected from multiple sources and includes a subset of samples from the original repositories.

Datasets are publicly available and collected from multiple sources, which contributes to increasing the heterogeneity due to different populations and different acquisition procedures across institutions. From each dataset, only eight clinically relevant classes are selected: Benign Keratosis (BEK), Dermatofibroma (DF), Benign Nevus (NEV), Vascular Lesion (VASC), Actinic Keratosis (ACK), Basal-Cell Carcinoma (BCC), Melanoma (MEL), Squamous Cell Cancer (SCC), which are the most adopted lesion types in scientific literature related to AI applications on dermatology. Other classes present in the original datasets were discarded to mitigate the effect of extreme class imbalance. Furthermore, several datasets are labeled with different terminology or classes; to guarantee consistency, some labels were mapped to the eight target classes. This approach standardizes the classes from the original datasets aiming to reduce class imbalance. Table 13 presents the origin of the repository, the number of samples selected and class mapping.

### Metadata pre-processing

Publicly available dermatology datasets mainly consist of images associated with heterogeneous metadata and annotations, with classes referring to specific skin lesions. Both metadata and annotations are heterogeneous, as they describe different types of information or different lesion categories. Metadata required pre-processing and mapping to shared medical terminology (i.e., vocabulary alignment) to prevent such limitations from hindering the learning process. For each dataset, metadata are structured in a database that includes the identified lesion (identified as the *current_class* variable during the template and prompting description in Clinical note generation structure, Supplementary Material), the nature of the class (*current_superclass*), a possible lesion specialization (*class_subclass_suffix*, which is a specific subclass specification for a lesion), and additional descriptive information (*current_description*). Moreover, two additional fields are introduced to align the lesion and its specialization with a common vocabulary, ensuring the use of consistent textual content: *current_match_class* matching *current_class*, *matching_subclass_suffix* matching *class_subclass_suffix*. The common vocabulary is the one presented on the zero-shot learning task. Regarding the lesion, the medical concepts (i.e., terminology to describe lesion information) adopted for the mapping in *current_match_class* are: ‘actinic keratosis’, ‘basal cell cancer’, ‘benign/seborrheic keratosis’, ‘dermatofibroma’, ‘melanocytic/benign nevus’, ‘melanoma’, ‘squamous cell cancer’, ‘vascular lesion’. Regarding the subclass specialization, the medical concepts adopted in *matching_subclass_suffix* for the mapping are: ‘actinic keratosis’, ‘basal cell carcinoma’, ‘benign melanocytic nevus’, ‘blue nevus’, ‘bowen disease/SCC in situ’, ‘congenital/special-pattern nevi’, ‘dermatofibroma/fibrous lesions’, ‘dysplastic/atypical nevus (clark-type)’, ‘lentigo maligna’, ‘lichenoid keratosis’, ‘melanoma’, ‘seborrheic keratosis & pigmented keratoses solar lentigo’, ‘squamous cell carcinoma (invasive)’, ‘vascular lesions’.

### Clinical note synthesis

The synthesis of clinical notes describing the skin lesion relies on the exploitation of metadata associated with the images and existing LLMs.

Most dermatologic datasets come with metadata that specify information such as lesion class (e.g., melanocytic nevus, melanoma) and, in certain cases, finer-grained annotations that map to one of the eight diagnostic categories used in this work (e.g., melanocytic nevus maps to Benign Nevus; Bowen’s disease maps to Actinic Keratosis). As discussed in the section “Metadata pre-processing”, such metadata are highly heterogeneous across datasets and primarily focus on lesion type, often lacking detailed morphological characteristics about the lesion. Among all possible metadata, we selected information about the lesion type, their malignant nature, possible subclass specification (when available), additional morphological information regarding the lesion (when available).

Lesion classes or subtypes can be leveraged as textual descriptors to populate a fixed template, leading to the first synthesis strategy, referred to in this paper as M (short for metadata).

Beyond metadata-based templating, textual descriptions of skin lesions can also be generated by prompting LLMs. Four LLMs are tested in this paper: GPT-4o-mini (non-medical), MedGemma (medical), SkinGPT4 and DermLIP (both dermatology-specific). This strategy enables to enrich skin lesion textual descriptions, exploiting targeted queries about specific lesion attributes. However, LLM-generated content may suffer from hallucinations or increased noise, which can negatively impact MM training. To mitigate these effects, the adoption of LLMs for clinical note synthesis is constrained in two ways. First, the LLM prompts are designed to focus also on non-specialized dermatologic aspects of the lesion, emphasizing natural descriptors such as asymmetry, color, and border shape, together with dermatologic aspects of a lesion. Second, metadata information is explicitly incorporated into the prompt to guide and condition the generation process. Metadata information added to the prompt is the same used to populate clinical note templates in previous strategy (M). This metadata-based prompting strategy is identified as L (short for LLM). It combines multiple prompts with different levels of detail, as described in Section Clinical note generation structure (Supplementary Materials), to increase variability during training. For the SkinGPT4 and DermLIP datasets, the prompts adopted are derived from the original model repositories. LLM-based synthesis without metadata conditioning is referred to as OL (Only LLM), described in Section Clinical note generation structure (Supplementary Materials) to illustrate how easily unconstrained prompting can lead to hallucinated or unreliable clinical descriptions. Finally, clinical notes generated with different strategies can be combined during training. The strategy (called LM) is described in Section LM setup: combination of L and M (Supplementary Materials), reporting performance on Supplementary Tables 8 and 9.

A relevant aspect to stress involves the use of metadata to generate clinical notes and prompts. Both L and M use the same type of metadata information. Strategy M uses the metadata information explicitly into the prompt, using it to populate the specific placeholders. On the other hand, strategy L uses the metadata to populate the prompt used to query a LLM. Depending on the LLM architecture, the information is not directly transferred to the clinical notes, as shown in Table 4 (capability of LLM to output the right type of lesion). Supplementary Table 3 shows how metadata are used for each strategy.

### Data pre-processing

Data pre-processing involves both visual and textual modalities, to produce inputs compatible with the respective encoders. The image encoder requires images that are 224 × 224 in size. Portions of images including skin lesions are cropped and then resized to the input size. The image pre-processing pipeline includes different elements, depending on image type. Dermoscopic images are segmented generating a lesion mask with Otsu thresholding and cropping the surrounding region. Clinical (non-dermoscopic) images are paired with manually annotated bounding boxes because heterogeneous acquisition conditions preclude a single automatic rule. The textual encoder requires tokenized input words, which are word units collected from the BERT vocabulary^74^ (including approximately 30,000 tokens). The BERT architecture accepts sequences that can include up to 512 tokens. The entire sequence is using the final hidden state associated with the designated special token, after the pooling layer.

### Data augmentation

The data augmentation strategy is applied to images during training to increase robustness of the model. It includes geometric transformations (90-degree rotations, horizontal and vertical flips) and color transformations on the RGB components of the images (range of perturbation is −50 to 10). The data augmentation aims to improve the generalization of algorithms on unseen datasets and addresses class imbalance, exploiting the class-wise data augmentation (CWDA) technique. The CWDA oversamples underrepresented classes, proposing augmented versions to the model. Clinical notes are not augmented with any transformation during training.

### Experimental setup

The experimental setup covers the hyperparameter optimization and how results are aggregated and reported. Hyperparameter selection was performed via grid search on the validation partition, which is strictly separate from all test partitions and was not accessed during final evaluation. Early stopping was applied based on validation loss; the final checkpoint corresponds to the epoch of minimum validation loss. Internal and external test partitions were not accessed during training or model development. The parameter exploration involves learning rates (1e-4, validating 1e-3, 1e-4, 1e-5), optimizer settings (Adam, validating Adam, SGD), batch size (32, validating 16, 32, 64), weight decay (1e-5, validating 1e-3, 1e-4, 1e-5, 1e-6) and epoch count (15 with early stopping, validating 10, 15, 20). Loss weights were tuned so that most terms received unit weight, while the self-supervised contrastive component was down-weighted (0.); the temperature used for NT-Xent is 0.5. The training of external components (clinical note generator) involves similar setups. For each report generation strategy, ten models are trained, using the same partitions but changing the initialization seeds, reporting the results as mean ± standard deviation across the ten runs. Statistical significance between conditions is assessed using the Wilcoxon signed-rank test^75^ applied pairwise across the ten runs; an asterisk (*) in result tables denotes *p* < 0.05. Foundation model baselines are evaluated at fixed publicly available checkpoints without retraining; standard deviations and statistical tests are therefore not available for these comparisons.

### Architectural choices

The development of the MM architecture involved multiple architectural choices: the choice of the image encoder, the choice of the loss function adopted to align the embeddings across modalities, the adoption of the classifier, the adoption of different LLMs to synthesize reports.

The choice of the image encoder turned to PanDerm, after testing small CNNs (best model DenseNet121, Section Experiments on other image-encoders, Supplementary Tables 1 and 2, Supplementary Materials). The initial assumptions on the choice of the CNN involved the limited size of training datasets, which could have led to overfitting. However, PanDerm, despite being larger and including more parameters, is pre-trained on dermatology images, guaranteeing a robust image representation. The choice of the loss function adopted to align the embeddings across modalities involves the comparison between NT-Xent and InfoNCE^76^, described in the Supplementary Material Section InfoNCE as loss function (Supplementary Tables 6 and 7). The adoption of the classifier in the MM architecture involves the comparison on the tasks of cross-modal retrieval and zero-shot learning, with results reported in Ablation Study: Classifier (Supplementary Materials).

### Hardware and software

The experiments are implemented using Python libraries. Deep learning algorithms are developed and trained with PyTorch 2.60. The BERT architecture is implemented using Transformers 4.52.4, which is also used to pre-process textual reports. All deep learning experiments are conducted on a V100x GPU. The model performance is evaluated using the metrics provided by scikit-learn 0.23. Numpy 2.0.2 is used to support other operations during training. Image pre-processing and augmentation are carried out with Albumentations^77^ 2.0.5. Clinical notes are synthesized using open 1.74.0, using GPT-4o-mini as backbone model (temperature set to 0.2, 250 tokens as output).

### Limitations and future works

Although the proposed method allows the synthesis of multimodal training pairs from limited unimodal image datasets, some limitations may limit its application in clinical practice. For example, the need for a supervised classifier, the lack of clinical evaluation, the limitation of noise within clinical notes, the restricted variability of generated reports, the limited size of real images, the labels adopted to evaluate the cross-modal retrieval task, and the problems to transfer the framework to other modalities.

The classifier head adopted on the multimodal architecture aims to mitigate overfitting given the limited size of the training partition. This choice does not follow the current state-of-the-art approach related to foundation models, with are task-agnostic and are only trained on the MM alignment^78^. The current trend aims to reduce the dependence on reliable annotations, which are time-consuming and require the workload of medical experts, under the hypothesis that large datasets can provide enough information to align modalities. This condition does not apply to the clinical scenario presented in the paper, where the training dataset is limited in size. The MM architecture, when trained without classifier, shows limited performance, as shown in Section Ablation Study: Classifier (Supplementary Materials). Furthermore, the adoption of a classifier forces the model to focus on a limited number of possible lesions (the classes), preventing the generalization of the architecture on completely different diseases.

Synthesized clinical notes are not reviewed by medical experts, which raises possible concerns regarding both their clinical validity and structural consistency. Although the proposed framework relies entirely on the automatic generation of medical notes, only the information related to the type of a skin lesion is validated, since the corresponding annotations are available. Additional aspects related to skin lesions are not manually revised, primarily due to the lack of annotations and the resulting need to involve a dermatologist, which is a time-consuming process requiring cross-validating images with their corresponding descriptions. The large number of clinical notes generated made the involvement of dermatologists impractical, thereby leaving room for potential hallucinations or noise introduced by LLMs regarding specific dermatology characteristics of a lesion. While the adopted strategies are designed to limit artifacts and hallucinated content on the information directly available in the metadata, it is not possible to estimate *ex ante* the proportion of noisy or unreliable notes, despite the results on downstream tasks are promising.

The absence of clinical validation also impacts the selection of categories used to query LLMs. Designing effective prompts would ideally require continuous feedback from medical experts to identify relevant elements, evaluate LLM outputs, and assess the influence of synthesized notes on multimodal (MM) algorithms. For instance, the categories proposed in this work to structure the synthesized clinical notes, symmetry, border, color, and dermoscopic structures, may not consistently capture the most clinically informative features, particularly for lesions that do not involve malignant conditions. When a clinical note emphasizes characteristics that are absent, ambiguous, or secondary in the visual content, the model may learn spurious links across modalities that fail to generalize to real clinical notes or to images acquired under different conditions. This mismatch is especially critical considering the zero-shot learning scenarios and in tasks that rely on semantic alignment between image embeddings and textual concept embeddings. In such cases, the shared latent space may overemphasize description-driven cues at the expense of image-driven diagnostic signals, ultimately limiting robustness and clinical relevance.

Report variability is constrained by the synthesis strategies employed. The collection of real notes from multiple sources would guarantee descriptions produced by multiple dermatologists, each one with his/her own style and focus on different details. Despite the combination of multiple notes, this limited spectrum of report styles may not reflect the clinical variability related to disease descriptions, limiting the flexibility of the representation. Consequently, the learned multimodal features may underperform when encountering real clinical notes that differ in sentence structure or clinical focus, reducing the translational potential of the approach.

The size of skin lesion images (224 × 224) allows to query LLMs and generate descriptions without major problems. However, medical images are usually acquired with high-quality standards, leading to larger images, which can be harder to feed to an LLM and would be more computationally expensive, especially considering other types of medical modalities (e.g., radiography or histopathology), such as histopathology or CT-scans. Larger medical images may include relevant findings corresponding to 1–2% of the image, which can be lost due to LLM pre-processing. Furthermore, the framework is not evaluated on non-medical imaging modalities (such as EEG), raising questions about its adoption.

The cross-modal retrieval evaluation adopts lesion-level classes as a proxy for alignment quality, not capturing features regarding the lesion morphology, such as the lesion asymmetry, border description or morphological features, due to the lack of annotations at this level of granularity. Future work with lesion-level annotations would enable finer-grained retrieval evaluation.

All these limitations light the path for future research and development efforts toward alleviating or overcoming the identified constraints. The first aspect to investigate involves the removal of the need for annotations and predefined classes, allowing the MM architecture to learn from a broader variety and diverse set of data (including exploiting of unlabeled data), providing a more robust tool to dermatologists. The second aspect involves clinical validation, which is essential given that the proposed architecture is designed to support dermatological practice. Establishing a development process based on continuous feedback and systematic assessment by dermatologists could substantially improve tool effectiveness, guiding the focus toward clinically relevant aspects. While clinical validation would be beneficial, the inherited noise included in the synthesized notes can be limited in the meanwhile. A possible approach would be to develop ensemble systems (like what medical experts do in clinical practice) with two LLMs describing a lesion and an additional network as tie-break, in case their outputs are not converging to a possible diagnosis. The combination of notes from multiple LLMs during training would also improve the variability, providing more heterogeneous content to the MM system and allowing to better filter noisy content. Finally, the idea of synthesizing textual descriptions can be extended beyond clinical notes to other data modalities, with appropriate adaptations. Such extensions would require prompt redesign and modality-specific considerations, with the possibility of leveraging similar strategies across heterogeneous data sources, further strengthening multimodal learning frameworks. For example, when considering other non-imaging medical modalities, a possible approach could involve prompting the LLM on general features, non-strictly modality-related, such as spikes, or introducing the feature investigated directly into the prompt.

## Supplementary information

Supplementary_material_clean.

## Data Availability

All datasets are publicly available: • BCN20000: https://api.isic-archive.com/collections/249/; • derm12345: https://dataverse.harvard.edu/dataset.xhtml?persistentId=doi:10.7910/DVN/DAXZ7P; • Derm7pt: https://derm.cs.sfu.ca/Welcome.html; • DermNet: https://www.kaggle.com/datasets/shubhamgoel27/dermnet; • FLUO_SC: https://data.mendeley.com/datasets/s8n68jj678/1; • MRA_MIDAS: https://aimi.stanford.edu/datasets/mra-midas-Multimodal-Image-Dataset-for-AI-based-Skin-Cancer; • HAM10000: https://dataverse.harvard.edu/dataset.xhtml?persistentId=doi:10.7910/DVN/DBW86T; • SKINL2: https://www.it.pt/AutomaticPage?id=3459; • Fitzpatrick17k: https://github.com/mattgroh/fitzpatrick17k; • HIBA: https://api.isic-archive.com/collections/251/; • PAD UFES 20: https://data.mendeley.com/datasets/zr7vgbcyr2/1; • SD198: https://huggingface.co/datasets/resyhgerwshshgdfghsdfgh/SD-198; • MSK: composed from https://api.isic-archive.com/collections/289/, https://api.isic-archive.com/collections/290/, https://api.isic-archive.com/collections/288/, https://api.isic-archive.com/collections/287/, https://api.isic-archive.com/collections/286/; • Milk10k: https://challenge.isic-archive.com/landing/milk10k/; • PH2: https://www.fc.up.pt/addi/ph2%20database.html. The synthesized clinical notes paired with images are generated using GPT-4o-mini, as described in the manuscript and in the Supplementary Material (Section Clinical note generation structure). The benchmark LLMs are publicly available: • MedGemma: https://huggingface.co/google/medgemma-4b-it; • SkinGPT4: https://github.com/JoshuaChou2018/SkinGPT-4?tab=readme-ov-file; • DermLIP: https://huggingface.co/moxeeeem/dermlip-gpt2-captioner. The benchmark foundation models are publicly available: • BioMedCLIP: https://huggingface.co/microsoft/BiomedCLIP-PubMedBERT_256-vit_base_patch16_224; • MedImageInsights: https://huggingface.co/lion-ai/MedImageInsights; • MONET: https://huggingface.co/suinleelab/monet; • Derm1M: https://huggingface.co/datasets/redlessone/Derm1M/tree/main.

## Code availability

Models and code to replicate the framework will be available, upon paper acceptance, at: https://github.com/antani-lab/Synthesized-Clinical-Notes-Multimodal-AI-Models.
