# Supplementary material for: Synthesized clinical notes enable training robust multimodal AI models from unimodal dermatology datasets
Source: NPJ Digit Med. 2026 Jul 17;9:873. doi: 10.1038/s41746-026-03040-3 (PMC13748555; doi:10.1038/s41746-026-03040-3)
Supplement: Supplementary file 1 [file 41746_2026_3040_MOESM1_ESM.docx]

Synthesized Clinical Notes Enable Training Robust Multimodal AI Models from Unimodal Dermatology Datasets – Supplementary Material

# Experiments on other image-encoders

This section compares the performance of two different image-encoders used to train the MM architecture: PanDerm (chosen) and DenseNet121, considering cross-modal retrieval and zero-shot learning as tasks. The two encoders are different: PanDerm is vision transformer (ViT), pre-trained on dermatology data, including around 85M parameters; while DenseNet121 is a CNN, pre-trained on ImageNet and including around 7M parameters. DenseNet121 was chosen because it guaranteed the most robust and highest performance, compared to other image encoders pre-trained on ImageNet (e.g. ResNet18).

Considering the cross-modal retrieval task (TableSupplementary 1), results show a clear difference in performance in favor of PanDerm, which reaches the highest performance in mostly every dataset.

The same trend is shown when considering the zero-shot learning task (TableSupplementary 2), with the model trained with PanDerm as image-encoder generally outperforming the same architecture trained with DenseNet121, in almost every dataset and setup, despite the 15-classes setup shows some more datasets where DenseNet reaches the highest performance.

TableSupplementary 1. Results on the cross-modal retrieval task for the MM architecture, comparing the performance reached when the image-encoder is a PanDerm or DenseNet121 architecture. The architecture is trained using L strategy (clinical notes synthesized with a LLM, prompted with metadata). Performance is reported as average mAP ± standard deviation for image-to-text (upper part) and text-to-image (lower part) retrieval across datasets. For each couple of columns, the highest performance is marked in bold.

| **Dataset** | **PanDerm, GPT** | **Densenet121, GPT** | **PanDerm, MedGemma** | **Densenet121, MedGemma** | **PanDerm, SkinGPT4** | **Densenet121, SkinGPT4** | **PanDerm, DermLIP** | **Densenet121, DermLIP** |
| --- | --- | --- | --- | --- | --- | --- | --- | --- |
| **Image-to-text** | | | | | | | | |
| **BCN20000** | **0.741 ± 0.022** | 0.715 ± 0.016 | **0.785 ± 0.013** | 0.749 ± 0.019 | **0.758 ± 0.013** | 0.708 ± 0.024 | **0.791 ± 0.012** | 0.705 ± 0.02 |
| **derm12345** | **0.846 ± 0.011** | 0.828 ± 0.007 | **0.872 ± 0.009** | 0.859 ± 0.011 | **0.861 ± 0.006** | 0.836 ± 0.02 | **0.879 ± 0.007** | 0.82 ± 0.011 |
| **Derm7pt** | **0.711 ± 0.027** | 0.686 ± 0.023 | **0.785 ± 0.032** | 0.737 ± 0.03 | **0.751 ± 0.01** | 0.737 ± 0.022 | **0.774 ± 0.023** | 0.622 ± 0.024 |
| **DermNet** | **0.612 ± 0.030** | 0.566 ± 0.019 | **0.717 ± 0.032** | 0.660 ± 0.01 | **0.696 ± 0.017** | 0.626 ± 0.021 | **0.717 ± 0.018** | 0.495 ± 0.039 |
| **FLUO_SC** | **0.682 ± 0.017** | 0.669 ± 0.02 | **0.737 ± 0.016** | 0.709 ± 0.031 | **0.726 ± 0.016** | 0.698 ± 0.011 | **0.736 ± 0.024** | 0.555 ± 0.052 |
| **MRA_MIDAS** | **0.538 ± 0.013** | 0.492 ± 0.014 | **0.591 ± 0.016** | 0.53 ± 0.014 | **0.526 ± 0.009** | 0.479 ± 0.028 | **0.597 ± 0.015** | 0.494 ± 0.023 |
| **HAM10000** | **0.754 ± 0.016** | 0.726 ± 0.01 | **0.785 ± 0.009** | 0.781 ± 0.01 | **0.767 ± 0.011** | 0.732 ± 0.026 | **0.788 ± 0.007** | 0.737 ± 0.015 |
| **SKINL2** | **0.750 ± 0.019** | 0.693 ± 0.028 | **0.808 ± 0.013** | 0.76 ± 0.037 | **0.767 ± 0.017** | 0.701 ± 0.023 | **0.804 ± 0.016** | 0.651 ± 0.014 |
| **Fitzpatrick17k** | **0.512 ± 0.036** | 0.432 ± 0.014 | **0.575 ± 0.030** | 0.507 ± 0.011 | **0.517 ± 0.016** | 0.486 ± 0.025 | **0.620 ± 0.012** | 0.503 ± 0.032 |
| **HIBA** | **0.524 ± 0.045** | 0.478 ± 0.03 | **0.577 ± 0.035** | 0.517 ± 0.033 | **0.569 ± 0.035** | 0.531 ± 0.012 | **0.593 ± 0.036** | 0.487 ± 0.038 |
| **PAD UFES 20** | **0.575 ± 0.022** | 0.549 ± 0.015 | **0.622 ± 0.020** | 0.596 ± 0.017 | **0.607 ± 0.022** | 0.567 ± 0.006 | **0.633 ± 0.022** | 0.513 ± 0.025 |
| **SD198** | **0.411 ± 0.041** | 0.458 ± 0.042 | **0.440 ± 0.037** | 0.514 ± 0.031 | 0.437 ± 0.037 | 0.469 ± 0.017 | **0.464 ± 0.034** | 0.405 ± 0.033 |
| **MSK** | **0.613 ± 0.015** | 0.600 ± 0.014 | **0.635 ± 0.009** | 0.646 ± 0.006 | **0.624 ± 0.013** | 0.601 ± 0.025 | **0.657 ± 0.005** | 0.615 ± 0.012 |
| **Milk10k (clinic)** | **0.499 ± 0.024** | 0.474 ± 0.024 | **0.573 ± 0.035** | 0.52 ± 0.023 | **0.522 ± 0.032** | 0.508 ± 0.034 | **0.571 ± 0.032** | 0.457 ± 0.018 |
| **Milk10k (dermo)** | **0.619 ± 0.024** | 0.600 ± 0.006 | **0.666 ± 0.013** | 0.622 ± 0.025 | **0.61 ± 0.016** | 0.575 ± 0.019 | **0.660 ± 0.012** | 0.578 ± 0.014 |
| **Text-to-image** | | | | | | | | |
| **BCN20000** | **0.580 ± 0.016** | 0.566 ± 0.011 | **0.711 ± 0.017** | 0.705 ± 0.016 | **0.510 ± 0.004** | 0.502 ± 0.014 | **0.756 ± 0.012** | 0.474 ± 0.021 |
| **derm12345** | **0.813 ± 0.007** | 0.805 ± 0.003 | **0.840 ± 0.012** | 0.826 ± 0.008 | **0.778 ± 0.020** | 0.761 ± 0.01 | **0.867 ± 0.005** | 0.754 ± 0.013 |
| **Derm7pt** | **0.448 ± 0.043** | 0.437 ± 0.047 | **0.613 ± 0.036** | 0.601 ± 0.031 | **0.371 ± 0.015** | 0.366 ± 0.019 | **0.681 ± 0.008** | 0.420 ± 0.030 |
| **DermNet** | 0.357 ± 0.033 | **0.38 ± 0.023** | **0.556 ± 0.034** | 0.549 ± 0.027 | **0.520 ± 0.022** | 0.508 ± 0.018 | **0.650 ± 0.007** | 0.321 ± 0.009 |
| **FLUO_SC** | **0.374 ± 0.025** | 0.362 ± 0.011 | **0.549 ± 0.044** | 0.526 ± 0.042 | **0.451 ± 0.015** | 0.444 ± 0.017 | **0.616 ± 0.007** | 0.284 ± 0.014 |
| **MRA_MIDAS** | **0.522 ± 0.027** | 0.510 ± 0.025 | **0.650 ± 0.024** | 0.642 ± 0.023 | **0.518 ± 0.010** | 0.512 ± 0.024 | **0.712 ± 0.012** | 0.44 ± 0.021 |
| **HAM10000** | **0.370 ± 0.035** | 0.345 ± 0.021 | **0.475 ± 0.039** | 0.422 ± 0.022 | 0.512 ± 0.047 | **0.525 ± 0.056** | 0.578 ± 0.019 | **0.600 ± 0.024** |
| **SKINL2** | 0.598 ± 0.022 | **0.615 ± 0.013** | 0.711 ± 0.024 | **0.72 ± 0.021** | **0.669 ± 0.014** | 0.655 ± 0.015 | **0.782 ± 0.005** | 0.565 ± 0.007 |
| **Fitzpatrick17k** | **0.401 ± 0.030** | 0.381 ± 0.029 | **0.619 ± 0.017** | 0.612 ± 0.026 | **0.471 ± 0.021** | 0.446 ± 0.026 | **0.640 ± 0.010** | 0.448 ± 0.01 |
| **HIBA** | **0.620 ± 0.013** | 0.601 ± 0.011 | 0.723 ± 0.014 | **0.741 ± 0.016** | **0.557 ± 0.004** | 0.537 ± 0.017 | **0.771 ± 0.011** | 0.522 ± 0.024 |
| **PAD UFES 20** | **0.551 ± 0.015** | 0.545 ± 0.015 | 0.613 ± 0.024 | **0.622 ± 0.017** | **0.433 ± 0.017** | 0.432 ± 0.033 | **0.661 ± 0.015** | 0.289 ± 0.038 |
| **SD198** | **0.471 ± 0.021** | 0.456 ± 0.011 | **0.554 ± 0.029** | 0.527 ± 0.023 | 0.509 ± 0.019 | **0.529 ± 0.015** | **0.622 ± 0.012** | 0.373 ± 0.007 |
| **MSK** | **0.607 ± 0.015** | 0.589 ± 0.008 | **0.740 ± 0.017** | 0.722 ± 0.013 | **0.567 ± 0.004** | 0.566 ± 0.012 | **0.767 ± 0.012** | 0.536 ± 0.014 |
| **Milk10k (clinic)** | **0.559 ± 0.017** | 0.579 ± 0.022 | 0.673 ± 0.023 | **0.686 ± 0.019** | 0.432 ± 0.012 | **0.437 ± 0.027** | **0.707 ± 0.015** | 0.363 ± 0.047 |
| **Milk10k (dermo)** | **0.559 ± 0.017** | 0.579 ± 0.022 | 0.673 ± 0.023 | **0.686 ± 0.019** | 0.432 ± 0.012 | **0.435 ± 0.029** | **0.707 ± 0.015** | 0.363 ± 0.047 |

TableSupplementary 2. Results on zero-shot learning task for the MM architecture, comparing the performance reached when the image-encoder is a PanDerm or DenseNet121 architecture. The architecture is trained using L strategy (clinical notes synthesized with a LLM, prompted with metadata). Performance is reported as average weighted F1-score ± standard deviation for 8-classes (upper part) and 15-classes (lower part) retrieval across datasets. For each couple of columns, the highest performance is marked in bold.

| **Dataset** | **PanDerm, GPT** | **Densenet121, GPT** | **PanDerm, MedGemma** | **Densenet121, MedGemma** | **PanDerm, SkinGPT4** | **Densenet121, SkinGPT4** | **PanDerm, DermLIP** | **Densenet121, DermLIP** |
| --- | --- | --- | --- | --- | --- | --- | --- | --- |
| **8-classes** | | | | | | | | |
| **BCN20000** | **0.738 ± 0.014** | 0.673 ± 0.054 | **0.718 ± 0.050** | 0.607 ± 0.031 | **0.724 ± 0.045** | 0.64 ± 0.025 | **0.691 ± 0.053** | 0.632 ± 0.038 |
| **derm12345** | **0.811 ± 0.026** | 0.758 ± 0.029 | **0.837 ± 0.016** | 0.783 ± 0.027 | **0.840 ± 0.017** | 0.816 ± 0.017 | **0.845 ± 0.017** | 0.817 ± 0.012 |
| **Derm7pt** | **0.744 ± 0.036** | 0.634 ± 0.128 | **0.664 ± 0.125** | 0.342 ± 0.094 | **0.669 ± 0.072** | 0.505 ± 0.071 | **0.670 ± 0.094** | 0.562 ± 0.057 |
| **DermNet** | **0.671 ± 0.064** | 0.563 ± 0.084 | **0.729 ± 0.103** | 0.599 ± 0.053 | **0.784 ± 0.080** | 0.642 ± 0.042 | **0.763 ± 0.077** | 0.627 ± 0.066 |
| **FLUO_SC** | **0.743 ± 0.052** | 0.712 ± 0.049 | **0.780 ± 0.061** | 0.726 ± 0.04 | **0.839 ± 0.033** | 0.789 ± 0.064 | 0.753 ± 0.082 | **0.801 ± 0.05** |
| **MRA_MIDAS** | **0.468 ± 0.026** | 0.41 ± 0.03 | **0.501 ± 0.021** | 0.434 ± 0.031 | **0.474 ± 0.017** | 0.432 ± 0.033 | **0.501 ± 0.018** | 0.44 ± 0.036 |
| **HAM10000** | **0.734 ± 0.012** | 0.707 ± 0.029 | **0.726 ± 0.018** | 0.694 ± 0.01 | **0.735 ± 0.015** | 0.718 ± 0.008 | **0.732 ± 0.015** | 0.715 ± 0.012 |
| **SKINL2** | **0.772 ± 0.025** | 0.693 ± 0.062 | **0.771 ± 0.029** | 0.656 ± 0.038 | **0.740 ± 0.051** | 0.673 ± 0.02 | **0.744 ± 0.023** | 0.637 ± 0.032 |
| **Fitzpatrick17k** | **0.516 ± 0.050** | 0.396 ± 0.049 | **0.533 ± 0.048** | 0.377 ± 0.039 | **0.525 ± 0.051** | 0.432 ± 0.1 | **0.545 ± 0.038** | 0.52 ± 0.024 |
| **HIBA** | **0.464 ± 0.073** | 0.355 ± 0.072 | **0.503 ± 0.041** | 0.391 ± 0.035 | **0.499 ± 0.065** | 0.442 ± 0.032 | **0.496 ± 0.031** | 0.394 ± 0.066 |
| **PAD UFES 20** | **0.523 ± 0.027** | 0.448 ± 0.023 | **0.542 ± 0.017** | 0.497 ± 0.02 | **0.567 ± 0.019** | 0.525 ± 0.017 | **0.570 ± 0.018** | 0.503 ± 0.02 |
| **SD198** | 0.367 ± 0.042 | **0.402 ± 0.016** | 0.353 ± 0.036 | **0.397 ± 0.033** | 0.384 ± 0.040 | **0.422 ± 0.042** | **0.429 ± 0.027** | 0.367 ± 0.036 |
| **MSK** | **0.570 ± 0.015** | 0.548 ± 0.03 | **0.550 ± 0.025** | 0.522 ± 0.022 | **0.573 ± 0.024** | 0.554 ± 0.01 | **0.569 ± 0.022** | 0.545 ± 0.022 |
| **Milk10k (clinic)** | **0.506 ± 0.027** | 0.493 ± 0.017 | **0.534 ± 0.025** | 0.507 ± 0.012 | **0.510 ± 0.026** | 0.487 ± 0.022 | **0.502 ± 0.023** | 0.499 ± 0.019 |
| **Milk10k (dermo)** | **0.642 ± 0.010** | 0.605 ± 0.022 | **0.627 ± 0.013** | 0.59 ± 0.023 | **0.613 ± 0.016** | 0.568 ± 0.027 | **0.618 ± 0.009** | 0.6 ± 0.018 |
| **15-classes** | | | | | | | | |
| **BCN20000** | 0.386 ± 0.015 | **0.398 ± 0.052** | **0.419 ± 0.107** | 0.346 ± 0.042 | 0.511 ± 0.087 | **0.555 ± 0.052** | **0.590 ± 0.009** | 0.552 ± 0.02 |
| **derm12345** | **0.450 ± 0.007** | 0.439 ± 0.035 | 0.440 ± 0.056 | **0.452 ± 0.006** | 0.156 ± 0.045 | **0.198 ± 0.034** | **0.464 ± 0.103** | 0.246 ± 0.003 |
| **Derm7pt** | **0.653 ± 0.047** | 0.496 ± 0.133 | **0.395 ± 0.151** | 0.205 ± 0.03 | **0.679 ± 0.020** | 0.646 ± 0.037 | **0.402 ± 0.116** | 0.242 ± 0.071 |
| **DermNet** | **0.570 ± 0.053** | 0.531 ± 0.073 | **0.537 ± 0.106** | 0.523 ± 0.038 | **0.739 ± 0.062** | 0.574 ± 0.054 | **0.642 ± 0.046** | 0.525 ± 0.065 |
| **FLUO_SC** | 0.436 ± 0.095 | **0.448 ± 0.077** | 0.282 ± 0.104 | **0.322 ± 0.094** | **0.650 ± 0.123** | 0.512 ± 0.153 | **0.727 ± 0.061** | 0.708 ± 0.138 |
| **MRA_MIDAS** | 0.233 ± 0.014 | **0.249 ± 0.022** | **0.256 ± 0.068** | 0.221 ± 0.023 | **0.314 ± 0.043** | 0.296 ± 0.035 | **0.391 ± 0.033** | 0.359 ± 0.022 |
| **HAM10000** | 0.156 ± 0.009 | **0.191 ± 0.019** | **0.341 ± 0.141** | 0.267 ± 0.066 | 0.279 ± 0.134 | **0.468 ± 0.151** | 0.554 ± 0.121 | **0.639 ± 0.026** |
| **SKINL2** | **0.373 ± 0.016** | 0.337 ± 0.055 | **0.426 ± 0.088** | 0.336 ± 0.043 | 0.425 ± 0.057 | **0.5 ± 0.043** | **0.525 ± 0.067** | 0.466 ± 0.034 |
| **Fitzpatrick17k** | 0.215 ± 0.019 | **0.288 ± 0.058** | **0.274 ± 0.086** | 0.205 ± 0.051 | 0.446 ± 0.013 | **0.466 ± 0.057** | **0.421 ± 0.021** | 0.397 ± 0.04 |
| **HIBA** | **0.207 ± 0.032** | 0.165 ± 0.026 | **0.208 ± 0.094** | 0.151 ± 0.044 | **0.300 ± 0.029** | 0.282 ± 0.043 | 0.301 ± 0.087 | **0.34 ± 0.065** |
| **PAD UFES 20** | **0.336 ± 0.048** | 0.325 ± 0.012 | **0.281 ± 0.053** | 0.269 ± 0.02 | **0.439 ± 0.066** | 0.395 ± 0.029 | **0.541 ± 0.019** | 0.424 ± 0.028 |
| **SD198** | **0.275 ± 0.021** | 0.253 ± 0.02 | **0.237 ± 0.033** | 0.229 ± 0.016 | 0.268 ± 0.035 | **0.31 ± 0.025** | **0.285 ± 0.018** | 0.24 ± 0.046 |
| **MSK** | **0.313 ± 0.012** | 0.285 ± 0.021 | 0.235 ± 0.029 | **0.274 ± 0.014** | **0.253 ± 0.009** | 0.25 ± 0.011 | **0.254 ± 0.055** | 0.194 ± 0.018 |
| **Milk10k (clinic)** | 0.374 ± 0.027 | **0.393 ± 0.023** | 0.300 ± 0.062 | **0.36 ± 0.038** | **0.368 ± 0.036** | 0.367 ± 0.031 | **0.422 ± 0.029** | 0.408 ± 0.019 |
| **Milk10k (dermo)** | 0.462 ± 0.009 | **0.480 ± 0.019** | 0.379 ± 0.083 | **0.44 ± 0.028** | **0.478 ± 0.032** | 0.461 ± 0.023 | **0.516 ± 0.011** | 0.505 ± 0.013 |

# Hallucinations and noisy LLM outputs

This section analyzes the hallucinated content of LLM outputs focusing on the lesion type. For each LLM, two confusion matrices are analyzed, considering the clinical note synthesized with L (synthetic clinical notes generated with a LLM, using metadata in the prompt) and OL (synthetic notes generated with a LLM, without any metadata in the prompt), shown in SupplementaryFigure 1. The Figure shows a clear trend on the accuracy of LLMs: when metadata are provided in the prompt (L) the errors are limited and the lesion is described with right lesion type, while instead when LLMs are prompted with metadata, clinical notes likely include the wrong lesion type. Among the LLMs, GPT and MedGemma show more robust predicitions, which can be explained considering the larger queries to prompt the model. An interesting detail involves the column UL (unlabeled) representing clinical notes not including any of the 8 lesion types analyzed in the paper. As predictable, OL strategies lead to higher numbers, with SkinGPT4 and DermLIP showing high percentage. This can be explained considering the content of the output, which can likely include artifacts when prompted without metadata.

SkinGPT4, when generating notes with OL, outputs descriptions of the image, often omitting details about the lesion type, but rather describing the anatomical part of the body where the lesion lies. Here is some examples:

- “This is an image of a woman's skin, showing various blemishes and acne. The skin tone is light brown, and the texture appears rough. Small brown spots and pimples cover the area, with some darker patches on the left side of the image. The image is taken from a close up angle, focusing on the woman's facial skin.”
- “This is an image of a woman's arm, showing the bone and muscle structure. The skin appears smooth and healthy, with visible veins running along the length of the arm. The color of the skin is light brown, matching the tone of the image. The woman's arm is thin but well-defined, indicating a fit and athletic build.”
- “This is an image of a woman's arm, showing the bone structure and muscles. The skin appears smooth and unblemished, with some small freckles and pores visible. The arm is covered by a thin layer of hair, which is darker in color than the surrounding skin. The bone structure is visible through the skin, with the humerus, radius, and ulna bones visible on the top and bottom of the image, respectively.”
- “This is an image of a woman's abdomen, showing her navel, skin, and other surrounding tissues. The image appears to be in black and white, with some minor contrast and shading to highlight the texture and details of the skin.”
- “The image shows a woman's torso with dark brown hair and a small mole on her left breast. Her skin appears smooth and unblemished, with visible veins visible on her arms and legs. She has no visible tattoos or scars. Her aureola is small and dark brown, surrounded by a thin ring of lighter skin. The image is in black and white.”

Clearly, the LLM cannot infer the location of the lesion, and it focuses on details that are not particularly relevant to describe specific aspects of the lesion. Furthermore, the architectural design of the network constrains the output, limiting the output tokens.

DermLIP shows similar limitations. It outputs personal information about the patient, which are clearly not true or inferred from the input image. For example:

- “Age (approx): 30, Anatomic site (general): anterior torso, Anatomic type (special): No, Concomitant biopsy: No, Diagnosis 1: Benign, Diagnosis 2: Benign melanocytic proliferations, Diagnosis 3: Nevus, Diagnosis”
- “Smokes: No, Alcohol use: No, Father background: GERMANY, Mother background: ITALY, Age (approx): 30, Pesticide exposure: No, Gender: Female, Skin cancer history: No, Diagnosis 1: Dermatofibroma, Diagnosis”
- “Age (approx): 45, Anatomic site (general): lower extremity, Anatomic region (special): Unknown, Concomitant biopsy: No, Diagnosis 1: Benign, Diagnosis 2: Benign melanocytic proliferations, Diagnosis 3: Nevus, Diagn”

In this case, the nationality of the patient cannot clearly being inferred from the image.

SupplementaryFigure 1. Overview of the LLMs capability to output the right lesion class. The Figure includes multiple confusion matrices reporting the percetange of correct class lesions, where the rows represent the correct class and the columns represent the predicted class. The last column (UL, unlabeled) represents the case when the LLM does not output any class. For each LLM, two confusion matrices are shown: the one obtained with L strategy (L – LLM, synthetic clinical notes generated with a LLM, using metadata in the prompt) and OL (OL – LLM, synthetic notes generated with a LLM, without any metadata in the prompt).

# Clinical note generation structure

Section Clinical note synthesis shows the strategies to generate clinical notes.

1. **M**: Metadata are used to populate a fixed template:

*“The image is classified within the* ***$current_superclass$*** *category. Its primary diagnostic class is* ***$current_class$*** *(****$class_subclass_suffix$****). Morphologically, it is characterized by* ***$current_description$****. The lesion falls within the* ***$current_match_class$*** *(****$matching_subclass_suffix$****) spectrum, representing a pattern consistent with this diagnostic group.”*

The elements reported in bold are variables, changing at run-time execution. **$*current_superclass$*** can be *benign*, *malignant*, *pre-cancerous*. **$*current_class$*** represents the lesion class and **$*class_subclass_suffix$*** its subclass (if reported in the metadata). **$*current_description$*** can include additional information. Finally **$*current_match_class$*** and **$*matching_subclass_suffix$*** are repetitions of the class and subclass, using a common vocabulary, as explained in Section Metadata Pre-processing. The same variables are used in the other prompt-based strategies, described in the following paragraphs.

2. **L**: The metadata-based prompt strategy, based on querying a LLM.

Clinical notes obtained from multiple prompts are combined during training.

**Prompt 1 (L1)**: The LLM selects predefined options for symmetry, border type, colors, and dermoscopic structures (e.g., pigment network, dots), expressed as bullet points, ensuring consistency and minimal hallucination. The prompt is split into two roles: instruction (system) and question (user), following modern chat model structures, where the system includes high-level directives (e.g. behavior, constraints, rules), while the user includes the actual query or task. It is used with gpt-4o-mini, MedGemma, SkinGPT4, DermLip. Variable values are highlighted in bold and are described in the M paragraph, except *specific_class_sentences,* which can include either the subclass specification, when available on the original metadata, or be empty.

instruction = “*You are a dermatologist. Your task is to describe the content in the image, using dermatologic terminology. You have to compile the structured report, using only the options provided among brackets. Report the category (Symmetric lesion, Border lesion, Color lesion, Dermoscopic structure). Your reply should be within 250 words. Underline explicitly that the lesion is* ***$current_superclass$*** *and includes a* ***$current_class$*** *(****class_subclass_suffix****) and it includes* ***$current_description$****.* ***$specific_class_sentence$****. Do not summarize at the end.*"

question = *"Report the following characteristics, choosing only the options among brackets, using the notation letter) (.e.g A), B)):*

A) *Asymmetry (only for the skin lesion)*

*Symmetric: Color and structure are mirrored across both axes.*

*Asymmetric: Uneven color or structure.*

*B) Border (only for the skin lesion)*

*Sharp border: Clear, abrupt pigment cut-off at the edge.*

*Indistinct border: Gradual or blurry transition at lesion edge.*

*Mixed border: Combination of sharp and indistinct segments.*

*C) Color (considering the lesion only, not the skin)*

*White (lighter than surrounding skin), Red, Light brown, Dark brown, Blue-gray, Black.*

*D) Dermoscopic Structures*

*Structureless areas: Uniform zones lacking visible patterns.*

*Pigment network: Reticular or mesh-like pigmentation.*

*Branched streaks: Irregular, atypical branching lines.*

*Dots: Small, round dark spots.*

*Globules: Larger, clustered round pigmented areas.”*

**Prompt 2 (L2)**: similar to Prompt 1, it includes less details describing the lesion to characterize it. It is used with gpt-4o-mini, MedGemma, SkinGPT4, DermLip. Variable values are highlighted in bold and are described in the M paragraph, except *specific_class_sentences,* which can include either an additional lesion description, depending on the original metadata, or be empty.

instruction = "*You are a dermatologist. Your task is to describe the content in the uploaded, using medical terminology. Be stick to what you identify, do not explain nature of diseases and do not imply. Your reply should be within 250 words. Underline explicitly that the lesion includes a* ***current_class*** *(****class_subclass_suffix****), (****current_superclass****) and some morphological characteristics are* ***current_description****.* ***specific_class_sentence****. Do not summarize at the end."*

question = “*What Characteristics of the dermatoscopic structure of the skin lesions you identify, among:*

*1) Type of Lesion: The report might describe whether the lesion is benign (e.g., mole, freckle) or suspicious for malignancy (e.g., melanoma, basal cell carcinoma).*

*Common types of lesions include:*

*Macules: Flat spots on the skin.*

*Papules: Raised, solid bumps.*

*Nodules: Larger, deeper lumps.*

*Plaques: Raised, flat areas.*

*Ulcers: Open sores.*

*Cysts: Fluid-filled sacs.*

*Vesicles/Bullae: Fluid-filled blisters.*

*2) Color: The color (e.g., brown, red, black, or pink) of the lesion is described as changes in color can be significant for malignancy.*

*3) Border Definition: Whether the lesion's edges are well-defined or irregular (which can suggest malignancy).*

*4) Symmetry: Whether the lesion is symmetric or asymmetric. Asymmetry may raise concern for malignancy.*”

**Prompt SkinGPT4**: additional prompts are used for SkinGPT4, aiming to mimic the ones used in the original publication. It includes a version with metadata and without metadata: the component *instruction* is attached to the prompt in the first case, while it is removed in the latter case. Variable values are highlighted in bold and are described in the M paragraph, except *specific_class_sentences,* which can include either an additional lesion description, depending on the original metadata, or be empty.

Possible prompt options (randomly chosen):

- *“Describe this image in detail.”,*
- *“Take a look at this image and describe what you notice.”,*
- *“Please provide a detailed description of the picture.”,*
- *“Could you describe the contents of this image for me?”*

instruction = “*Underline explicitly that the lesion includes a* ***current_class*** *(****class_subclass_suffix)****,* ***current_superclass*** *and some morphological characteristics are* ***current_description****.* ***specific_class_sentence***.”

**Prompt DermLip**: additional prompts are used for DermLip, aiming to mimic the ones used in the original publication. It includes a version with metadata and without metadata: the component instruction is removed in the latter case. Variable values are highlighted in bold and are described in the M paragraph, except *specific_class_sentences,* which can include either an additional lesion description, depending on the original metadata, or be empty.

prompt = "*Describe the skin lesion concisely (morphology, color, scale, border, location) in one sentence. Conclude with the most likely diagnosis 250 words)*."

instruction = “*Underline explicitly that the lesion includes a* ***current_class*** *(****class_subclass_suffix****),* ***current_superclass*** *and some morphological characteristics are* ***current_description****.* ***specific_class_sentence****.”*

TableSupplementary 3 reports how metadata are mapped into variables and adopted into the clinical note templates and to prompt LLMs, differentiating for each specific strategy (M, L, OL).

TableSupplementary 3. Overview of the metadata application across different strategies. The Table includes the description of each variable, used for clinical note template or prompting, and its use on M, L and OL strategies.

| **Information** | **Description** | **M** | **L** | **OL** |
| --- | --- | --- | --- | --- |
| **current_superclass** | Benign/Pre-Cancerous/Malignant | Embodied in clinical note | Used in the prompt | Not used |
| **current_class** | Lesion class | Embodied in clinical note | Used in the prompt | Not used |
| **class_subclass_suffix** | Lesion class specification (if available) | Embodied in clinical note | Used in the prompt | Not used |
| **current_description** | Additional morphological information | Embodied in clinical note | Used in the prompt | Not used |
| **current_match_class** | Current_class mapping | Embodied in clinical note | Used in the prompt | Not used |
| **matching_subclass_suffix** | class_subclass_suffix mapping | Embodied in clinical note | Used in the prompt | Not used |

# Loss function to align representation

The alignment of modalities is achieved through the optimization of a loss function including three complementary terms: the normalized temperature-scaled cross-entropy (NT-Xent) loss, the L1 loss, and the cosine similarity loss. Each term is designed to address a specific limitation of multimodal learning, particularly in limited training dataset setups. Their combination enforces semantic consistency, direct cross-modal alignment, and geometric regularization of the embedding space. Formally, the overall objective is defined as:

$$L=L_{NT\_Xent}+L_{L1}+ L_{Cos}$$

No weighting coefficients are introduced between the three terms. During the design of the architecture, the unweighted combination provided the most stable optimization and the best performance. Introducing additional weights did not lead to consistent improvements and increased the complexity of the training procedure.

The normalized temperature-scaled cross-entropy loss (NT-Xent) is a contrastive objective originally introduced in SimCLR^1^ to learn representations by pulling together different augmented views of the same sample while pushing apart all other samples within the minibatch. Given a batch of B samples, two stochastic augmentations are applied, producing 2B embeddings. For each anchor embedding, the corresponding augmented view is treated as the single positive, while the remaining 2(B−1) embeddings are treated as negatives. Similarity is measured via cosine similarity and scaled by a temperature parameter. This formulation is fully self-supervised and assumes that all non-matching samples are semantically unrelated. While effective for large batches, NT-Xent can be problematic in small batch setups (such as the one proposed in the paper) when samples belong to a limited number of classes. In this setting, embeddings from the same class but different instances are treated as negatives, and the loss explicitly pushes them apart. With only a few samples per batch and eight classes, this repulsive effect can dominate and hinder the learning of class-consistent representations. To address this limitation, a supervised variant of NT-Xent is adopted. Unlike the standard formulation, which treats all non-matching samples as negatives, this approach leverages class labels to distinguish between semantically similar and dissimilar samples. Samples belonging to the same class are treated as positives, while samples from different classes are treated as negatives. The loss is computed by combining contributions from both groups. This modification aims to prevent samples belonging to the same class to be pushed apart in the latent space, making the loss more stable and semantically consistent in small-batch, multi-class settings.

The L1 loss (mean absolute error) is applied directly to paired image and text embeddings, to minimize their element-wise absolute differences. Its role is to enforce a strict alignment between corresponding multimodal representations, encouraging them to occupy nearby regions in the embedding space. Unlike contrastive objectives, the L1 loss operates at the representation level and provides a stable, scale-sensitive constraint that complements the relative comparisons imposed by NT-Xent.

Finally, the cosine similarity loss forces alignment by minimizing the angular distance between paired image and text embeddings. By focusing on the orientation of vectors rather than their magnitude, this loss enforces directional consistency between modalities and helps preserve structures in the embedding space. When combined with the L1 loss, it prevents degenerate solutions in which representations collapse into a small cluster, a risk that is particularly pronounced when training on relatively small multimodal datasets.

# Ablation study: Classifier

The section investigates the important of the classifier component in the MM architecture, comparing the performance of the model trained with (Cl) and without (No Cl) the optimization of the classifier. The first setup is the one presented in the paper, while the second involves the optimization of the modality alignment only. For both architectures, the model is trained using the L strategy (clinical notes synthesized with LLM, using metadata in the prompt). The evaluation involves two tasks: cross-modal retrieval and zero-shot learning.

Results on cross-modal retrieval (TableSupplementary 8) show that the classifier generally improves the performance in most datasets, with large gaps (10-30 points if mAP). While in the image-to-text setup all the performance difference is largely in favor of the MM architecture trained with a classifier, the text-to-image setup shows smaller gaps, sometimes with the MM trained without a classifier reaching higher performance.

Results on zero-shot learning (TableSupplementary 9) show a trend similar to image-to-text images, since the MM architecture trained with a classifier leads to large performance gaps (10-30 points in F1-score).

The explanation for this gap can be identified in the training dataset: the limited amount of training samples (around 16,000 image-text couples) does not fit with the requirements of self-supervised algorithms used to align modalities, leading the model to overfit on training data. Furthermore, the classifier provides an additional optimization that helps to regularize the multimodal representation, forcing separation between a small set of discriminative disease categories (e.g. the eight classes). However, the classifier represents a limitation, since it forces the model to be trained with annotated data.

TableSupplementary 4. Results on the ablation study regarding the classifier component, evaluated on the cross-modal task for the MM architecture. The Table compares the performance considering the L strategy, comparing the performance reached training the model with (reported as name LLM – Cl) and without a classifier (reported as name LLM – No Cl). Performance is reported as average mAP ± standard deviation for image-to-text setup (upper part) and text-to-image setup (lower part) across datasets. For each couple of columns, the highest performance is marked in bold.

| **Dataset** | **L, GPT** | **LM, GPT** | **L, MedGemma** | **LM, MedGemma** | **L, SkinGPT4** | **LM, SkinGPT4** | **L, DermLIP** | **LM, DermLIP** |
| --- | --- | --- | --- | --- | --- | --- | --- | --- |
| **Image-to-text** | | | | | | | | |
| **BCN20000** | **0.741 ± 0.022** | 0.456 ± 0.024 | **0.785 ± 0.013** | 0.530 ± 0.028 | **0.758 ± 0.013** | 0.540 ± 0.010 | **0.791 ± 0.012** | 0.526 ± 0.014 |
| **derm12345** | **0.846 ± 0.011** | 0.710 ± 0.010 | **0.872 ± 0.009** | 0.779 ± 0.025 | **0.861 ± 0.006** | 0.753 ± 0.033 | **0.879 ± 0.007** | 0.760 ± 0.021 |
| **Derm7pt** | **0.711 ± 0.027** | 0.531 ± 0.047 | **0.785 ± 0.032** | 0.514 ± 0.047 | **0.751 ± 0.01** | 0.493 ± 0.055 | **0.774 ± 0.023** | 0.472 ± 0.092 |
| **DermNet** | **0.612 ± 0.03** | 0.441 ± 0.021 | **0.717 ± 0.032** | 0.42 ± 0.035 | **0.696 ± 0.017** | 0.42 ± 0.042 | **0.717 ± 0.018** | 0.425 ± 0.036 |
| **FLUO_SC** | **0.682 ± 0.017** | 0.492 ± 0.051 | **0.737 ± 0.016** | 0.475 ± 0.034 | **0.726 ± 0.016** | 0.506 ± 0.029 | **0.736 ± 0.024** | 0.452 ± 0.018 |
| **MRA_MIDAS** | **0.538 ± 0.013** | 0.459 ± 0.011 | **0.591 ± 0.016** | 0.447 ± 0.03 | **0.526 ± 0.009** | 0.445 ± 0.025 | **0.597 ± 0.015** | 0.464 ± 0.028 |
| **HAM10000** | **0.754 ± 0.016** | 0.598 ± 0.012 | **0.785 ± 0.009** | 0.685 ± 0.016 | **0.767 ± 0.011** | 0.675 ± 0.034 | **0.788 ± 0.007** | 0.643 ± 0.034 |
| **SKINL2** | **0.75 ± 0.019** | 0.634 ± 0.018 | **0.808 ± 0.013** | 0.638 ± 0.023 | **0.767 ± 0.017** | 0.592 ± 0.052 | **0.804 ± 0.016** | 0.658 ± 0.018 |
| **Fitzpatrick17k** | **0.512 ± 0.036** | 0.401 ± 0.049 | **0.575 ± 0.030** | 0.427 ± 0.049 | **0.517 ± 0.016** | 0.369 ± 0.023 | **0.62 ± 0.012** | 0.395 ± 0.039 |
| **HIBA** | **0.524 ± 0.045** | 0.466 ± 0.029 | **0.577 ± 0.035** | 0.444 ± 0.02 | **0.569 ± 0.035** | 0.486 ± 0.028 | **0.593 ± 0.036** | 0.484 ± 0.036 |
| **PAD UFES 20** | **0.575 ± 0.022** | 0.45 ± 0.033 | **0.622 ± 0.020** | 0.432 ± 0.016 | **0.607 ± 0.022** | 0.481 ± 0.03 | **0.633 ± 0.022** | 0.463 ± 0.023 |
| **SD198** | **0.411 ± 0.041** | 0.338 ± 0.043 | **0.44 ± 0.037** | 0.335 ± 0.008 | **0.437 ± 0.037** | 0.315 ± 0.053 | **0.464 ± 0.034** | 0.367 ± 0.023 |
| **MSK** | **0.613 ± 0.015** | 0.457 ± 0.015 | **0.635 ± 0.009** | 0.523 ± 0.016 | **0.624 ± 0.013** | 0.521 ± 0.023 | **0.657 ± 0.005** | 0.515 ± 0.025 |
| **Milk10k (clinic)** | **0.499 ± 0.024** | 0.419 ± 0.035 | **0.573 ± 0.035** | 0.412 ± 0.039 | **0.522 ± 0.032** | 0.48 ± 0.053 | **0.571 ± 0.032** | 0.496 ± 0.033 |
| **Milk10k (dermo)** | **0.619 ± 0.024** | 0.366 ± 0.031 | **0.666 ± 0.013** | 0.425 ± 0.046 | **0.610 ± 0.016** | 0.476 ± 0.039 | **0.660 ± 0.012** | 0.492 ± 0.031 |
| **Text-to-image** | | | | | | | | |
| **BCN20000** | 0.580 ± 0.016 | **0.596 ± 0.017** | **0.711 ± 0.017** | 0.565 ± 0.015 | 0.51 ± 0.004 | 0.587 ± 0.026 | **0.756 ± 0.012** | 0.584 ± 0.026 |
| **derm12345** | **0.813 ± 0.007** | 0.809 ± 0.014 | **0.840 ± 0.012** | 0.774 ± 0.023 | 0.778 ± 0.02 | 0.768 ± 0.048 | **0.867 ± 0.005** | 0.797 ± 0.019 |
| **Derm7pt** | **0.448 ± 0.043** | 0.321 ± 0.029 | **0.613 ± 0.036** | 0.338 ± 0.042 | 0.371 ± 0.015 | 0.325 ± 0.064 | **0.681 ± 0.008** | 0.343 ± 0.055 |
| **DermNet** | **0.357 ± 0.033** | 0.296 ± 0.008 | **0.556 ± 0.034** | 0.331 ± 0.028 | **0.520 ± 0.022** | 0.32 ± 0.026 | **0.650 ± 0.007** | 0.315 ± 0.039 |
| **FLUO_SC** | **0.374 ± 0.025** | 0.380 ± 0.013 | **0.549 ± 0.044** | 0.406 ± 0.024 | **0.451 ± 0.015** | 0.409 ± 0.016 | **0.616 ± 0.007** | 0.395 ± 0.023 |
| **MRA_MIDAS** | **0.522 ± 0.027** | 0.421 ± 0.016 | **0.650 ± 0.024** | 0.483 ± 0.017 | **0.518 ± 0.01** | 0.463 ± 0.021 | **0.712 ± 0.012** | 0.487 ± 0.012 |
| **HAM10000** | **0.37 ± 0.035** | 0.35 ± 0.017 | **0.475 ± 0.039** | 0.375 ± 0.041 | **0.512 ± 0.047** | 0.278 ± 0.012 | **0.578 ± 0.019** | 0.46 ± 0.017 |
| **SKINL2** | **0.598 ± 0.022** | 0.426 ± 0.02 | **0.711 ± 0.024** | 0.499 ± 0.024 | **0.669 ± 0.014** | 0.496 ± 0.022 | **0.782 ± 0.005** | 0.504 ± 0.024 |
| **Fitzpatrick17k** | **0.401 ± 0.03** | 0.296 ± 0.016 | **0.619 ± 0.017** | 0.399 ± 0.044 | **0.471 ± 0.021** | 0.368 ± 0.047 | **0.640 ± 0.010** | 0.391 ± 0.05 |
| **HIBA** | **0.62 ± 0.013** | 0.606 ± 0.02 | **0.723 ± 0.014** | 0.576 ± 0.017 | 0.557 ± 0.004 | **0.598 ± 0.029** | **0.771 ± 0.011** | 0.599 ± 0.022 |
| **PAD UFES 20** | **0.551 ± 0.015** | 0.473 ± 0.025 | **0.613 ± 0.024** | 0.437 ± 0.027 | 0.433 ± 0.017 | **0.460 ± 0.044** | **0.661 ± 0.015** | 0.433 ± 0.024 |
| **SD198** | **0.471 ± 0.021** | 0.351 ± 0.02 | **0.554 ± 0.029** | 0.358 ± 0.023 | **0.509 ± 0.019** | 0.36 ± 0.035 | **0.622 ± 0.012** | 0.353 ± 0.029 |
| **MSK** | 0.607 ± 0.015 | **0.669 ± 0.017** | **0.740 ± 0.017** | 0.630 ± 0.025 | 0.567 ± 0.004 | **0.637 ± 0.043** | **0.767 ± 0.012** | 0.641 ± 0.035 |
| **Milk10k (clinic)** | 0.559 ± 0.017 | **0.561 ± 0.029** | **0.673 ± 0.023** | 0.534 ± 0.027 | 0.432 ± 0.012 | **0.557 ± 0.041** | **0.707 ± 0.015** | 0.539 ± 0.024 |
| **Milk10k (dermo)** | 0.559 ± 0.017 | **0.561 ± 0.029** | **0.673 ± 0.023** | 0.534 ± 0.027 | 0.432 ± 0.012 | **0.557 ± 0.041** | **0.707 ± 0.015** | 0.539 ± 0.024 |

TableSupplementary 5. Results on the ablation study regarding the classifier component, evaluated on the zero-shot learning task for the MM architecture. The Table compares the performance considering the L strategy, comparing the performance reached training the model with (reported as name LLM – Cl) and without a classifier (reported as name LLM – No Cl). Performance is reported as average F1-score ± standard deviation for 8-classes setup (upper part) and 15-classes setup (lower part) across datasets. For each couple of columns, the highest performance is marked in bold.

| **Dataset** | **GPT - Cl** | **GPT – No Cl** | **MedGemma - Cl** | **MedGemma – No Cl** | **SkinGPT4 - Cl** | **SkinGPT4 – No Cl** | **DermLIP - Cl** | **DermLIP – No Cl** |
| --- | --- | --- | --- | --- | --- | --- | --- | --- |
| **8-classes** | | | | | | | | |
| **BCN20000** | **0.738 ± 0.014** | 0.278 ± 0.07 | **0.718 ± 0.05** | 0.363 ± 0.147 | **0.724 ± 0.045** | 0.445 ± 0.086 | **0.691 ± 0.053** | 0.372 ± 0.073 |
| **derm12345** | **0.811 ± 0.026** | 0.553 ± 0.269 | **0.837 ± 0.016** | 0.295 ± 0.233 | **0.84 ± 0.017** | 0.631 ± 0.111 | **0.845 ± 0.017** | 0.689 ± 0.095 |
| **Derm7pt** | **0.744 ± 0.036** | 0.441 ± 0.194 | **0.664 ± 0.125** | 0.111 ± 0.026 | **0.669 ± 0.072** | 0.163 ± 0.081 | **0.670 ± 0.094** | 0.193 ± 0.162 |
| **DermNet** | **0.671 ± 0.064** | 0.414 ± 0.067 | **0.729 ± 0.103** | 0.323 ± 0.112 | **0.784 ± 0.08** | 0.247 ± 0.045 | **0.763 ± 0.077** | 0.221 ± 0.052 |
| **FLUO_SC** | **0.743 ± 0.052** | 0.410 ± 0.133 | **0.78 ± 0.061** | 0.281 ± 0.156 | **0.839 ± 0.033** | 0.331 ± 0.035 | **0.753 ± 0.082** | 0.226 ± 0.112 |
| **MRA_MIDAS** | **0.468 ± 0.026** | 0.258 ± 0.07 | **0.501 ± 0.021** | 0.090 ± 0.030 | **0.474 ± 0.017** | 0.273 ± 0.033 | **0.501 ± 0.018** | 0.286 ± 0.051 |
| **HAM10000** | **0.734 ± 0.012** | 0.355 ± 0.188 | **0.726 ± 0.018** | 0.352 ± 0.197 | **0.735 ± 0.015** | 0.603 ± 0.063 | **0.732 ± 0.015** | 0.582 ± 0.049 |
| **SKINL2** | **0.772 ± 0.025** | 0.526 ± 0.167 | **0.771 ± 0.029** | 0.233 ± 0.102 | **0.74 ± 0.051** | 0.456 ± 0.117 | **0.744 ± 0.023** | 0.448 ± 0.090 |
| **Fitzpatrick17k** | **0.516 ± 0.05** | 0.344 ± 0.122 | **0.533 ± 0.048** | 0.099 ± 0.047 | **0.525 ± 0.051** | 0.215 ± 0.091 | **0.545 ± 0.038** | 0.346 ± 0.062 |
| **HIBA** | **0.464 ± 0.073** | 0.324 ± 0.116 | **0.503 ± 0.041** | 0.045 ± 0.023 | **0.499 ± 0.065** | 0.200 ± 0.084 | **0.496 ± 0.031** | 0.250 ± 0.064 |
| **PAD UFES 20** | **0.523 ± 0.027** | 0.357 ± 0.037 | **0.542 ± 0.017** | 0.234 ± 0.085 | **0.567 ± 0.019** | 0.267 ± 0.045 | **0.57 ± 0.018** | 0.290 ± 0.074 |
| **SD198** | **0.367 ± 0.042** | 0.277 ± 0.048 | **0.353 ± 0.036** | 0.209 ± 0.044 | **0.384 ± 0.04** | 0.228 ± 0.021 | **0.429 ± 0.027** | 0.299 ± 0.017 |
| **MSK** | **0.570 ± 0.015** | 0.304 ± 0.081 | **0.55 ± 0.025** | 0.257 ± 0.121 | **0.573 ± 0.024** | 0.412 ± 0.059 | **0.569 ± 0.022** | 0.400 ± 0.043 |
| **Milk10k (clinic)** | **0.506 ± 0.027** | 0.433 ± 0.027 | **0.534 ± 0.025** | 0.147 ± 0.069 | **0.51 ± 0.026** | 0.245 ± 0.099 | **0.502 ± 0.023** | 0.225 ± 0.098 |
| **Milk10k (dermo)** | **0.642 ± 0.010** | 0.349 ± 0.131 | **0.627 ± 0.013** | 0.470 ± 0.076 | **0.613 ± 0.016** | 0.530 ± 0.030 | **0.618 ± 0.009** | 0.431 ± 0.160 |
| **15-classes** | | | | | | | | |
| **BCN20000** | **0.386 ± 0.015** | 0.228 ± 0.082 | **0.419 ± 0.107** | 0.342 ± 0.12 | **0.511 ± 0.087** | 0.303 ± 0.107 | **0.590 ± 0.009** | 0.366 ± 0.083 |
| **derm12345** | **0.45 ± 0.007** | 0.37 ± 0.078 | **0.44 ± 0.056** | 0.289 ± 0.103 | **0.156 ± 0.045** | 0.114 ± 0.049 | **0.464 ± 0.103** | 0.181 ± 0.023 |
| **Derm7pt** | **0.653 ± 0.047** | 0.238 ± 0.123 | **0.395 ± 0.151** | 0.098 ± 0.025 | **0.679 ± 0.02** | 0.123 ± 0.074 | **0.402 ± 0.116** | 0.217 ± 0.219 |
| **DermNet** | **0.57 ± 0.053** | 0.384 ± 0.08 | **0.537 ± 0.106** | 0.255 ± 0.081 | **0.739 ± 0.062** | 0.271 ± 0.061 | **0.642 ± 0.046** | 0.264 ± 0.033 |
| **FLUO_SC** | **0.436 ± 0.095** | 0.254 ± 0.096 | **0.282 ± 0.104** | 0.061 ± 0.018 | **0.65 ± 0.123** | 0.071 ± 0.016 | **0.727 ± 0.061** | 0.117 ± 0.017 |
| **MRA_MIDAS** | **0.233 ± 0.014** | 0.185 ± 0.049 | **0.256 ± 0.068** | 0.108 ± 0.061 | **0.314 ± 0.043** | 0.188 ± 0.019 | **0.391 ± 0.033** | 0.192 ± 0.015 |
| **HAM10000** | **0.156 ± 0.009** | 0.237 ± 0.2 | **0.341 ± 0.141** | 0.458 ± 0.192 | **0.279 ± 0.134** | 0.314 ± 0.221 | **0.554 ± 0.121** | 0.531 ± 0.067 |
| **SKINL2** | **0.373 ± 0.016** | 0.357 ± 0.108 | **0.426 ± 0.088** | 0.284 ± 0.077 | **0.425 ± 0.057** | 0.286 ± 0.087 | **0.525 ± 0.067** | 0.348 ± 0.064 |
| **Fitzpatrick17k** | **0.215 ± 0.019** | 0.23 ± 0.093 | **0.274 ± 0.086** | 0.045 ± 0.026 | **0.446 ± 0.013** | 0.126 ± 0.069 | **0.421 ± 0.021** | 0.185 ± 0.063 |
| **HIBA** | **0.207 ± 0.032** | 0.184 ± 0.091 | **0.208 ± 0.094** | 0.039 ± 0.027 | **0.3 ± 0.029** | 0.106 ± 0.061 | **0.301 ± 0.087** | 0.199 ± 0.068 |
| **PAD UFES 20** | **0.336 ± 0.048** | 0.282 ± 0.04 | **0.281 ± 0.053** | 0.126 ± 0.04 | **0.439 ± 0.066** | 0.115 ± 0.058 | **0.541 ± 0.019** | 0.133 ± 0.033 |
| **SD198** | **0.275 ± 0.021** | 0.199 ± 0.013 | **0.237 ± 0.033** | 0.161 ± 0.016 | **0.268 ± 0.035** | 0.15 ± 0.023 | **0.285 ± 0.018** | 0.193 ± 0.022 |
| **MSK** | **0.313 ± 0.012** | 0.173 ± 0.047 | **0.235 ± 0.029** | 0.135 ± 0.027 | **0.253 ± 0.009** | 0.09 ± 0.046 | **0.254 ± 0.055** | 0.088 ± 0.036 |
| **Milk10k (clinic)** | **0.374 ± 0.027** | 0.304 ± 0.034 | **0.300 ± 0.062** | 0.071 ± 0.029 | **0.368 ± 0.036** | 0.100 ± 0.065 | **0.422 ± 0.029** | 0.107 ± 0.029 |
| **Milk10k (dermo)** | **0.462 ± 0.009** | 0.302 ± 0.145 | **0.379 ± 0.083** | 0.359 ± 0.158 | **0.478 ± 0.032** | 0.329 ± 0.139 | **0.516 ± 0.011** | 0.360 ± 0.160 |

# Classification results

This section describes the classification performance of the MM architecture, comparing multiple setups: Img (the unimodal training of the architecture using only images), M (multimodal training using clinical notes generated with metadata), OL (multimodal training using clinical notes synthesized with a LLM, without any metadata), L (multimodal training using clinical notes synthesized with a LLM, with metadata included in the prompt). TableSupplementary 5 reports the results, as weighted F1-score and standard deviation.

The MM training of the architecture leads to improving classification performance, with the exception of SD198. However, differently from what is observed for cross-modal retrieval and zero-shot learning, the adoption of clinical notes generated without using metadata does not negatively impact classification performance. In particular, OL reaches the highest performance on half of the datasets. This result is counterintuitive, since the content of those clinical notes can be noisy, potentially forcing the model to learn inaccurate cross-modal relationships. A possible explanation for these results can be identified in the optimization of the model, which involves two groups of functions, the classification terms and the alignment functions. The classifier output is mostly influenced by the first couple of terms, therefore the effects of noise within the clinical notes have smaller effects. For the same reason, the effects of noise on the two other tasks are more pronounced because they involve the last layer before the classifier, where the alignment is forced with the loss functions.

In addition, the results suggest that classification benefits from the inclusion of textual inputs, independently of their specific content. Even when the clinical notes are noisy or weakly informative, their presence provides complementary information that supports the learning of more discriminative visual representations. This indicates that the MM training, rather than the semantic accuracy of the text, plays a key role in improving classification performance. Therefore, textual inputs act as an auxiliary source of supervision that enhances robustness during training.

TableSupplementary 6. Results on the classification performance of the MM architecture. The Table compares the performance considering multiple strategies: the architecture trained with images only (Img), M, OL (for each LLM) and L (for each LLM). Performance is reported as average F1-score ± standard deviation across datasets. For each dataset, the highest performance is marked in bold.

| **Dataset** | **Img** | **M** | **OL-GPT** | **L-GPT** | **OL-MedGemma** | **L-MedGemma** | **OL-SkinGPT4** | **L- SkinGPT4** | **OL-DermLIP** | **L-DermLIP** |
| --- | --- | --- | --- | --- | --- | --- | --- | --- | --- | --- |
| **BCN20000** | 0.764 ± 0.01 | 0.736 ± 0.041 | 0.760 ± 0.021 | 0.759 ± 0.013 | 0.78 ± 0.01 | 0.759 ± 0.013 | 0.771 ± 0.011 | **0.783 ± 0.011** | 0.739 ± 0.046 | 0.721 ± 0.058 |
| **derm12345** | 0.854 ± 0.009 | 0.879 ± 0.039 | **0.892 ± 0.041** | 0.849 ± 0.012 | 0.854 ± 0.01 | 0.849 ± 0.012 | 0.855 ± 0.007 | 0.845 ± 0.011 | 0.848 ± 0.019 | 0.833 ± 0.023 |
| **Derm7pt** | 0.77 ± 0.032 | 0.745 ± 0.052 | 0.780 ± 0.029 | 0.744 ± 0.040 | **0.781 ± 0.026** | 0.744 ± 0.04 | 0.753 ± 0.012 | 0.773 ± 0.031 | 0.742 ± 0.039 | 0.779 ± 0.054 |
| **DermNet** | 0.695 ± 0.036 | 0.629 ± 0.101 | 0.686 ± 0.028 | 0.691 ± 0.034 | 0.667 ± 0.033 | 0.691 ± 0.034 | **0.700 ± 0.051** | 0.683 ± 0.039 | 0.654 ± 0.05 | 0.610 ± 0.086 |
| **FLUO_SC** | 0.707 ± 0.034 | 0.661 ± 0.066 | 0.693 ± 0.027 | 0.693 ± 0.032 | **0.719 ± 0.023** | 0.693 ± 0.032 | 0.717 ± 0.014 | 0.717 ± 0.011 | 0.67 ± 0.033 | 0.661 ± 0.028 |
| **MRA_MIDAS** | 0.523 ± 0.026 | 0.463 ± 0.047 | 0.512 ± 0.026 | 0.513 ± 0.012 | 0.515 ± 0.022 | 0.513 ± 0.012 | 0.499 ± 0.018 | **0.538 ± 0.011** | 0.509 ± 0.033 | 0.451 ± 0.087 |
| **HAM10000** | 0.747 ± 0.01 | 0.743 ± 0.015 | 0.750 ± 0.009 | 0.747 ± 0.010 | **0.752 ± 0.007** | 0.747 ± 0.01 | 0.751 ± 0.005 | 0.747 ± 0.004 | 0.748 ± 0.018 | 0.751 ± 0.015 |
| **SKINL2** | 0.788 ± 0.016 | 0.765 ± 0.044 | 0.768 ± 0.025 | 0.778 ± 0.022 | **0.791 ± 0.028** | 0.778 ± 0.022 | 0.771 ± 0.012 | 0.775 ± 0.011 | 0.767 ± 0.048 | 0.756 ± 0.035 |
| **Fitzpatrick17k** | 0.533 ± 0.042 | 0.565 ± 0.029 | 0.571 ± 0.026 | 0.567 ± 0.034 | 0.530 ± 0.023 | 0.567 ± 0.034 | 0.567 ± 0.027 | 0.547 ± 0.05 | **0.577 ± 0.020** | 0.552 ± 0.044 |
| **HIBA** | 0.511 ± 0.038 | 0.5 ± 0.049 | 0.517 ± 0.023 | 0.509 ± 0.064 | 0.518 ± 0.02 | 0.509 ± 0.064 | 0.526 ± 0.043 | **0.546 ± 0.033** | 0.529 ± 0.033 | 0.51 ± 0.06 |
| **PAD UFES 20** | 0.557 ± 0.026 | 0.538 ± 0.035 | 0.565 ± 0.018 | 0.568 ± 0.021 | **0.588 ± 0.031** | 0.568 ± 0.021 | 0.577 ± 0.022 | 0.572 ± 0.016 | 0.565 ± 0.019 | 0.571 ± 0.03 |
| **SD198** | **0.421 ± 0.05** | 0.403 ± 0.047 | 0.400 ± 0.035 | 0.407 ± 0.051 | 0.406 ± 0.042 | 0.407 ± 0.051 | 0.383 ± 0.037 | 0.404 ± 0.04 | 0.42 ± 0.038 | 0.389 ± 0.042 |
| **MSK** | 0.572 ± 0.014 | 0.574 ± 0.021 | 0.565 ± 0.013 | 0.579 ± 0.012 | 0.578 ± 0.01 | 0.579 ± 0.012 | 0.576 ± 0.008 | 0.575 ± 0.015 | **0.581 ± 0.022** | 0.574 ± 0.013 |
| **Milk10k (clinic)** | 0.504 ± 0.037 | 0.472 ± 0.053 | 0.490 ± 0.028 | 0.505 ± 0.034 | 0.53 ± 0.018 | 0.505 ± 0.034 | 0.501 ± 0.023 | **0.532 ± 0.014** | 0.496 ± 0.033 | 0.463 ± 0.039 |
| **Milk10k (dermo)** | 0.609 ± 0.038 | 0.602 ± 0.023 | 0.594 ± 0.019 | **0.623 ± 0.013** | 0.611 ± 0.019 | 0.623 ± 0.013 | 0.6 ± 0.012 | 0.622 ± 0.022 | 0.619 ± 0.014 | 0.605 ± 0.029 |

# InfoNCE as loss function

This section compares two self-supervised loss functions used to align modality representations: NT-Xent (adopted in the paper) and InfoNCE^2^. The first setup is the one presented in the paper, while the second involves the optimization of the modality alignment only. For both architectures, the model is trained using the L strategy (clinical notes synthesized with LLM, using metadata in the prompt). The evaluation involves two tasks: cross-modal retrieval and zero-shot learning.

Considering the cross-modal retrieval tasks (TableSupplementary 6), the model trained with NT-Xent leads to generally higher results, considering both tasks, despite not being consistently the highest. While there are isolated cases where InfoNCE matches or slightly exceeds NT-Xent, the performance differences are not systematic across models.

The results on zero-shot learning tasks (TableSupplementary 7) confirm the trend: in both the setups, NT-Xent generally reaches the highest performance, with relevant gaps, despite being outperformed in some datasets.

Considering both tasks together, the results suggest that although InfoNCE can be competitive and provide robust performance in some retrieval cases, NT-Xent provides the most reliable improvements across multiple datasets and tasks, therefore it was seleced as the self-supervised alignment loss in the paper.

TableSupplementary 7. Results on cross-modal retrieval for the MM architecture, comparing two loss functions to align representations across modalities: NT-Xent and InfoNCE. The MM architecture is trained using strategy L (LLM prompts including metadata information), considering the four LLMs. Performance is reported as average mAP ± standard deviation for image-to-text setup (upper part) and text-to-image setup (lower part) across datasets; * denotes statistically significant differences between M and L (Wilcoxon test). For each couple of columns, the highest performance is marked in bold.

| **Dataset** | **NT-Xent, GPT** | **InfoNCE, GPT** | **NT-Xent, MedGemma** | **InfoNCE, MedGemma** | **NT-Xent, SkinGPT4** | **InfoNCE, SkinGPT4** | **NT-Xent, DermLIP** | **InfoNCE, DermLIP** |
| --- | --- | --- | --- | --- | --- | --- | --- | --- |
| **Image-to-text** | | | | | | | | |
| **BCN20000** | **0.741 ± 0.022** | 0.713 ± 0.011 | **0.785 ± 0.013** | 0.763 ± 0.006 | **0.758 ± 0.013** | 0.722 ± 0.023 | **0.791 ± 0.012** | 0.691 ± 0.018 |
| **derm12345** | **0.846 ± 0.011** | 0.838 ± 0.011 | **0.872 ± 0.009** | 0.871 ± 0.01 | **0.861 ± 0.006** | 0.855 ± 0.006 | **0.879 ± 0.007** | 0.793 ± 0.019 |
| **Derm7pt** | **0.711 ± 0.027** | 0.702 ± 0.04 | **0.785 ± 0.032** | 0.748 ± 0.026 | **0.751 ± 0.01** | 0.731 ± 0.017 | **0.774 ± 0.023** | 0.606 ± 0.072 |
| **DermNet** | 0.612 ± 0.03 | **0.662 ± 0.027** | 0.717 ± 0.032 | **0.721 ± 0.023** | **0.696 ± 0.017** | 0.688 ± 0.021 | **0.717 ± 0.018** | 0.592 ± 0.021 |
| **FLUO_SC** | 0.682 ± 0.017 | **0.736 ± 0.021** | **0.737 ± 0.016** | 0.752 ± 0.009 | **0.726 ± 0.016** | 0.687 ± 0.019 | **0.736 ± 0.024** | 0.527 ± 0.014 |
| **MRA_MIDAS** | 0.538 ± 0.013 | **0.548 ± 0.006** | **0.591 ± 0.016** | 0.581 ± 0.006 | **0.526 ± 0.009** | 0.521 ± 0.016 | **0.597 ± 0.015** | 0.524 ± 0.016 |
| **HAM10000** | **0.754 ± 0.016** | 0.727 ± 0.011 | **0.785 ± 0.009** | 0.779 ± 0.011 | **0.767 ± 0.011** | 0.757 ± 0.01 | **0.788 ± 0.007** | 0.731 ± 0.012 |
| **SKINL2** | 0.75 ± 0.019 | **0.763 ± 0.007** | **0.808 ± 0.013** | 0.804 ± 0.022 | **0.767 ± 0.017** | 0.762 ± 0.012 | **0.804 ± 0.016** | 0.725 ± 0.016 |
| **Fitzpatrick17k** | **0.512 ± 0.036** | 0.481 ± 0.038 | **0.575 ± 0.03** | 0.492 ± 0.04 | **0.517 ± 0.016** | 0.491 ± 0.009 | **0.620 ± 0.012** | 0.435 ± 0.037 |
| **HIBA** | 0.524 ± 0.045 | **0.583 ± 0.018** | 0.577 ± 0.035 | **0.589 ± 0.013** | 0.569 ± 0.035 | **0.586 ± 0.024** | **0.593 ± 0.036** | 0.493 ± 0.032 |
| **PAD UFES 20** | 0.575 ± 0.022 | **0.601 ± 0.013** | 0.622 ± 0.02 | **0.633 ± 0.01** | **0.607 ± 0.022** | 0.543 ± 0.023 | **0.633 ± 0.022** | 0.52 ± 0.012 |
| **SD198** | **0.411** ± **0.041** | 0.38 ± 0.057 | 0.440 ± 0.037 | **0.448 ± 0.058** | **0.437 ± 0.037** | 0.4 ± 0.036 | **0.464 ± 0.034** | 0.375 ± 0.015 |
| **MSK** | **0.613 ± 0.015** | 0.592 ± 0.004 | **0.635 ± 0.009** | 0.634 ± 0.004 | **0.624 ± 0.013** | 0.614 ± 0.009 | **0.657 ± 0.005** | 0.59 ± 0.01 |
| **Milk10k (clinic)** | 0.499 ± 0.024 | **0.586 ± 0.023** | 0.573 ± 0.035 | **0.618 ± 0.016** | **0.522 ± 0.032** | 0.514 ± 0.015 | **0.571 ± 0.032** | 0.533 ± 0.017 |
| **Milk10k (dermo)** | 0.619 ± 0.024 | **0.629 ± 0.016** | 0.666 ± 0.013 | **0.673 ± 0.007** | **0.61 ± 0.016** | 0.602 ± 0.026 | **0.660 ± 0.012** | 0.64 ± 0.013 |
| **Text-to-image** | | | | | | | | |
| **BCN20000** | 0.580 ± 0.016 | **0.621 ± 0.008** | **0.711 ± 0.017** | 0.707 ± 0.01 | 0.510 ± 0.004 | **0.586 ± 0.020** | **0.756 ± 0.012** | 0.581 ± 0.023 |
| **derm12345** | **0.813 ± 0.007** | 0.805 ± 0.008 | **0.840 ± 0.012** | 0.839 ± 0.006 | **0.778 ± 0.02** | 0.767 ± 0.006 | **0.867 ± 0.005** | 0.752 ± 0.018 |
| **Derm7pt** | 0.448 ± 0.043 | **0.557 ± 0.02** | **0.613 ± 0.036** | 0.605 ± 0.027 | **0.371 ± 0.015** | 0.332 ± 0.009 | **0.681 ± 0.008** | 0.379 ± 0.028 |
| **DermNet** | 0.357 ± 0.033 | **0.512 ± 0.022** | 0.556 ± 0.034 | **0.591 ± 0.012** | **0.520 ± 0.022** | 0.507 ± 0.007 | **0.650 ± 0.007** | 0.360 ± 0.011 |
| **FLUO_SC** | 0.374 ± 0.025 | **0.545 ± 0.022** | **0.549 ± 0.044** | 0.537 ± 0.023 | 0.451 ± 0.015 | **0.525 ± 0.02** | **0.616 ± 0.007** | 0.375 ± 0.017 |
| **MRA_MIDAS** | 0.522 ± 0.027 | **0.619 ± 0.011** | 0.650 ± 0.024 | **0.671 ± 0.009** | 0.518 ± 0.01 | **0.577 ± 0.024** | **0.712 ± 0.012** | 0.535 ± 0.021 |
| **HAM10000** | 0.37 ± 0.035 | **0.396 ± 0.028** | 0.475 ± 0.039 | **0.580 ± 0.032** | **0.512 ± 0.047** | 0.398 ± 0.045 | **0.578 ± 0.019** | 0.567 ± 0.017 |
| **SKINL2** | 0.598 ± 0.022 | **0.676 ± 0.016** | 0.711 ± 0.024 | **0.740 ± 0.010** | **0.669 ± 0.014** | 0.637 ± 0.008 | **0.782 ± 0.005** | 0.562 ± 0.013 |
| **Fitzpatrick17k** | 0.401 ± 0.03 | **0.512 ± 0.015** | **0.619 ± 0.017** | 0.606 ± 0.014 | 0.471 ± 0.021 | **0.494 ± 0.024** | **0.640 ± 0.01** | 0.535 ± 0.019 |
| **HIBA** | 0.62 ± 0.013 | **0.657 ± 0.007** | **0.723 ± 0.014** | 0.718 ± 0.009 | 0.557 ± 0.004 | **0.606 ± 0.02** | **0.771 ± 0.011** | 0.599 ± 0.015 |
| **PAD UFES 20** | 0.551 ± 0.015 | **0.619 ± 0.011** | **0.613 ± 0.024** | 0.607 ± 0.017 | 0.433 ± 0.017 | **0.504 ± 0.042** | **0.661 ± 0.015** | 0.415 ± 0.041 |
| **SD198** | 0.471 ± 0.021 | **0.583 ± 0.02** | 0.554 ± 0.029 | **0.583 ± 0.028** | **0.509 ± 0.019** | 0.505 ± 0.012 | **0.622 ± 0.012** | 0.395 ± 0.017 |
| **MSK** | 0.607 ± 0.015 | **0.665 ± 0.007** | **0.740 ± 0.017** | 0.729 ± 0.009 | 0.567 ± 0.004 | **0.636 ± 0.012** | **0.767 ± 0.012** | 0.626 ± 0.017 |
| **Milk10k (clinic)** | 0.559 ± 0.017 | **0.630 ± 0.014** | **0.673 ± 0.023** | 0.651 ± 0.009 | 0.432 ± 0.012 | **0.529 ± 0.033** | **0.707 ± 0.015** | 0.509 ± 0.038 |
| **Milk10k (dermo)** | 0.559 ± 0.017 | **0.630 ± 0.014** | **0.673 ± 0.023** | 0.651 ± 0.009 | 0.432 ± 0.012 | **0.529 ± 0.033** | **0.707 ± 0.015** | 0.509 ± 0.038 |

TableSupplementary 8. Results on the zero-shot learning task for the MM architecture, comparing two loss functions to align representations across modalities: NT-Xent and InfoNCE. The MM architecture is trained using strategy L (LLM prompts including metadata information), considering the four LLMs.. Performance is reported as average F1-score ± standard deviation for 8-classes setup (upper part) and 15-classes setup (lower part) across datasets; * denotes statistically significant differences between M and L (Wilcoxon test). For each couple of columns, the highest performance is marked in bold.

| **Dataset** | **NT-Xent, GPT** | **InfoNCE, GPT** | **NT-Xent, MedGemma** | **InfoNCE, MedGemma** | **NT-Xent, SkinGPT4** | **InfoNCE, SkinGPT4** | **NT-Xent, DermLIP** | **InfoNCE, DermLIP** |
| --- | --- | --- | --- | --- | --- | --- | --- | --- |
| **8-classes** | | | | | | | | |
| **BCN20000** | **0.738 ± 0.014** | 0.657 ± 0.032 | **0.718 ± 0.05** | 0.471 ± 0.025 | **0.724 ± 0.045** | 0.585 ± 0.088 | **0.691 ± 0.053** | 0.677 ± 0.036 |
| **derm12345** | **0.811 ± 0.026** | 0.744 ± 0.099 | **0.837 ± 0.016** | 0.754 ± 0.013 | **0.840 ± 0.017** | 0.831 ± 0.015 | **0.845 ± 0.017** | 0.830 ± 0.010 |
| **Derm7pt** | **0.744 ± 0.036** | 0.714 ± 0.062 | **0.664 ± 0.125** | 0.214 ± 0.017 | **0.669 ± 0.072** | 0.564 ± 0.089 | **0.670 ± 0.094** | 0.576 ± 0.055 |
| **DermNet** | **0.671 ± 0.064** | 0.648 ± 0.053 | **0.729 ± 0.103** | 0.574 ± 0.048 | **0.784 ± 0.08** | 0.665 ± 0.068 | **0.763 ± 0.077** | 0.668 ± 0.034 |
| **FLUO_SC** | **0.743 ± 0.052** | 0.388 ± 0.039 | **0.78 ± 0.061** | 0.518 ± 0.165 | **0.839 ± 0.033** | 0.798 ± 0.057 | **0.753 ± 0.082** | 0.739 ± 0.056 |
| **MRA_MIDAS** | **0.468 ± 0.026** | 0.379 ± 0.053 | **0.501 ± 0.021** | 0.392 ± 0.031 | **0.474 ± 0.017** | 0.455 ± 0.034 | **0.501 ± 0.018** | 0.485 ± 0.017 |
| **HAM10000** | **0.734 ± 0.012** | 0.720 ± 0.031 | **0.726 ± 0.018** | 0.655 ± 0.007 | **0.735 ± 0.015** | 0.691 ± 0.032 | **0.732 ± 0.015** | 0.716 ± 0.008 |
| **SKINL2** | **0.772 ± 0.025** | 0.745 ± 0.044 | **0.771 ± 0.029** | 0.643 ± 0.014 | **0.740 ± 0.051** | 0.669 ± 0.045 | **0.744 ± 0.023** | 0.702 ± 0.033 |
| **Fitzpatrick17k** | **0.516 ± 0.05** | 0.323 ± 0.016 | **0.533 ± 0.048** | 0.134 ± 0.044 | **0.525 ± 0.051** | 0.421 ± 0.106 | **0.545 ± 0.038** | 0.501 ± 0.022 |
| **HIBA** | **0.464 ± 0.073** | 0.394 ± 0.056 | **0.503 ± 0.041** | 0.370 ± 0.025 | **0.499 ± 0.065** | 0.512 ± 0.025 | **0.496 ± 0.031** | 0.52 ± 0.035 |
| **PAD UFES 20** | **0.523 ± 0.027** | 0.347 ± 0.018 | **0.542 ± 0.017** | 0.423 ± 0.054 | **0.567 ± 0.019** | 0.482 ± 0.052 | **0.57 ± 0.018** | 0.487 ± 0.045 |
| **SD198** | **0.367 ± 0.042** | 0.314 ± 0.045 | **0.353 ± 0.036** | 0.255 ± 0.038 | **0.384 ± 0.040** | 0.35 ± 0.041 | **0.429 ± 0.027** | 0.297 ± 0.028 |
| **MSK** | **0.570 ± 0.015** | 0.537 ± 0.012 | **0.550 ± 0.025** | 0.433 ± 0.010 | **0.573 ± 0.024** | 0.507 ± 0.042 | **0.569 ± 0.022** | 0.559 ± 0.011 |
| **Milk10k (clinic)** | **0.506 ± 0.027** | 0.444 ± 0.043 | **0.534 ± 0.025** | 0.489 ± 0.001 | **0.51 ± 0.026** | 0.482 ± 0.044 | **0.502 ± 0.023** | 0.546 ± 0.012 |
| **Milk10k (dermo)** | **0.642 ± 0.010** | 0.565 ± 0.016 | **0.627 ± 0.013** | 0.536 ± 0.024 | **0.613 ± 0.016** | 0.454 ± 0.110 | **0.618 ± 0.009** | 0.602 ± 0.009 |
| **15-classes** | | | | | | | | |
| **BCN20000** | 0.386 ± 0.015 | **0.522 ± 0.062** | **0.419 ± 0.107** | 0.236 ± 0.046 | **0.511 ± 0.087** | 0.444 ± 0.087 | **0.590 ± 0.009** | 0.523 ± 0.054 |
| **derm12345** | **0.450 ± 0.007** | 0.437 ± 0.061 | **0.44 ± 0.056** | 0.422 ± 0.01 | 0.156 ± 0.045 | **0.197 ± 0.051** | **0.464 ± 0.103** | 0.240 ± 0.007 |
| **Derm7pt** | **0.653 ± 0.047** | 0.448 ± 0.159 | **0.395 ± 0.151** | 0.153 ± 0.023 | **0.679 ± 0.02** | 0.635 ± 0.036 | **0.402 ± 0.116** | 0.292 ± 0.136 |
| **DermNet** | **0.57 ± 0.053** | 0.567 ± 0.061 | **0.537 ± 0.106** | 0.525 ± 0.031 | **0.739 ± 0.062** | 0.491 ± 0.046 | **0.642 ± 0.046** | 0.612 ± 0.041 |
| **FLUO_SC** | **0.436 ± 0.095** | 0.374 ± 0.042 | 0.282 ± 0.104 | **0.357 ± 0.031** | **0.650 ± 0.123** | 0.178 ± 0.040 | **0.727 ± 0.061** | 0.725 ± 0.064 |
| **MRA_MIDAS** | 0.233 ± 0.014 | **0.315 ± 0.028** | **0.256 ± 0.068** | 0.199 ± 0.019 | **0.314 ± 0.043** | 0.224 ± 0.053 | **0.391 ± 0.033** | 0.359 ± 0.024 |
| **HAM10000** | 0.156 ± 0.009 | **0.551 ± 0.085** | **0.341 ± 0.141** | 0.149 ± 0.079 | 0.279 ± 0.134 | **0.547 ± 0.157** | 0.554 ± 0.121 | **0.632 ± 0.035** |
| **SKINL2** | 0.373 ± 0.016 | **0.516 ± 0.076** | **0.426 ± 0.088** | 0.306 ± 0.023 | **0.425 ± 0.057** | 0.416 ± 0.072 | 0.525 ± 0.067 | **0.533 ± 0.033** |
| **Fitzpatrick17k** | **0.215 ± 0.019** | 0.165 ± 0.063 | **0.274 ± 0.086** | 0.043 ± 0.007 | **0.446 ± 0.013** | 0.276 ± 0.038 | **0.421 ± 0.021** | 0.346 ± 0.042 |
| **HIBA** | **0.207 ± 0.032** | 0.173 ± 0.071 | **0.208 ± 0.094** | 0.148 ± 0.006 | **0.300 ± 0.029** | 0.282 ± 0.083 | 0.301 ± 0.087 | **0.427 ± 0.054** |
| **PAD UFES 20** | 0.336 ± 0.048 | **0.329 ± 0.018** | **0.281 ± 0.053** | 0.272 ± 0.010 | **0.439 ± 0.066** | 0.115 ± 0.031 | **0.541 ± 0.019** | 0.473 ± 0.041 |
| **SD198** | **0.275 ± 0.021** | 0.224 ± 0.04 | **0.237 ± 0.033** | 0.192 ± 0.027 | **0.268 ± 0.035** | 0.23 ± 0.037 | **0.285 ± 0.018** | 0.178 ± 0.019 |
| **MSK** | **0.313 ± 0.012** | 0.196 ± 0.017 | **0.235 ± 0.029** | 0.207 ± 0.012 | **0.253 ± 0.009** | 0.157 ± 0.016 | **0.254 ± 0.055** | 0.188 ± 0.016 |
| **Milk10k (clinic)** | 0.374 ± 0.027 | **0.400 ± 0.026** | 0.300 ± 0.062 | **0.364 ± 0.012** | **0.368 ± 0.036** | 0.095 ± 0.021 | 0.422 ± 0.029 | **0.470 ± 0.013** |
| **Milk10k (dermo)** | **0.462 ± 0.009** | 0.460 ± 0.011 | 0.379 ± 0.083 | **0.380 ± 0.011** | **0.478 ± 0.032** | 0.195 ± 0.079 | **0.516 ± 0.011** | 0.515 ± 0.015 |

# LM setup: combination of L and M

The section compares two strategies to train the MM architecture: **L** involves clinical notes synthesized using LLM prompted with metadata information; LM involves two types of clinical notes, L and M, which are clinical notes generated filling a fixed template with metadata. The evaluation involves two tasks: cross-modal retrieval and zero-shot learning.

Results on cross-modal retrieval (TableSupplementary 8) show that LM generally improves the task performance, considering both setups, especially in text-to-image, while the improvement in image-to-text is lower.

Results on zero-shot learning (TableSupplementary 9) show a different trend, since both strategies lead to similar performance, without one outperforming the other, considering the 8-classes setup and 15-classes setup.

TableSupplementary 9. Results on the cross-modal retrieval task for the MM architecture, comparing the performance reached training the model with clinical notes synthesized prompting a LLM providing lesion metadata (L; GPT, MedGemma, SkinGPT4, DermLIP) and combining the same notes with generated filling a template with metadata (LM). Performance is reported as average mAP ± standard deviation for image-to-text (upper part) and text-to-image (lower part) retrieval across datasets; * denotes statistically significant differences between M and L (Wilcoxon test). For each couple of columns, the highest performance is marked in bold.

| **Dataset** | **L, GPT** | **LM, GPT** | **L, MedGemma** | **LM, MedGemma** | **L, SkinGPT4** | **LM, SkinGPT4** | **L, DermLIP** | **LM, DermLIP** |
| --- | --- | --- | --- | --- | --- | --- | --- | --- |
| **Image-to-text** | | | | | | | | |
| **BCN20000** | 0.741 ± 0.022 | **0.790 ± 0.015** | 0.785 ± 0.013 | **0.800 ± 0.013** | 0.758 ± 0.013 | **0.801 ± 0.009** | **0.791 ± 0.012** | 0.747 ± 0.022 |
| **derm12345** | 0.846 ± 0.011 | **0.867 ± 0.007** | 0.872 ± 0.009 | **0.875 ± 0.009** | 0.861 ± 0.006 | **0.875 ± 0.003** | **0.879 ± 0.007** | 0.835 ± 0.02 |
| **Derm7pt** | 0.711 ± 0.027 | **0.78 ± 0.039** | **0.785 ± 0.032** | 0.78 ± 0.021 | 0.751 ± 0.01 | **0.796 ± 0.022** | **0.774 ± 0.023** | 0.701 ± 0.046 |
| **DermNet** | 0.612 ± 0.03 | **0.705 ± 0.029** | 0.717 ± 0.032 | **0.731 ± 0.024** | 0.696 ± 0.017 | **0.75 ± 0.011** | **0.717 ± 0.018** | 0.592 ± 0.028 |
| **FLUO_SC** | 0.682 ± 0.017 | **0.738 ± 0.022** | 0.737 ± 0.016 | **0.758 ± 0.012** | 0.726 ± 0.016 | **0.756 ± 0.022** | **0.736 ± 0.024** | 0.660 ± 0.030 |
| **MRA_MIDAS** | 0.538 ± 0.013 | **0.583 ± 0.02** | 0.591 ± 0.016 | **0.592 ± 0.024** | 0.526 ± 0.009 | **0.573 ± 0.017** | **0.597 ± 0.015** | 0.556 ± 0.011 |
| **HAM10000** | 0.754 ± 0.016 | **0.788 ± 0.007** | 0.785 ± 0.009 | **0.793 ± 0.011** | 0.767 ± 0.011 | **0.792 ± 0.008** | **0.788 ± 0.007** | 0.751 ± 0.012 |
| **SKINL2** | 0.75 ± 0.019 | **0.789 ± 0.025** | **0.808 ± 0.013** | 0.802 ± 0.028 | 0.767 ± 0.017 | **0.782 ± 0.016** | **0.804 ± 0.016** | 0.739 ± 0.012 |
| **Fitzpatrick17k** | 0.512 ± 0.036 | **0.57 ± 0.036** | 0.575 ± 0.03 | **0.593 ± 0.027** | 0.517 ± 0.016 | **0.583 ± 0.011** | **0.62 ± 0.012** | 0.572 ± 0.044 |
| **HIBA** | 0.524 ± 0.045 | **0.57 ± 0.025** | **0.577 ± 0.035** | 0.571 ± 0.037 | 0.569 ± 0.035 | **0.590 ± 0.040** | **0.593 ± 0.036** | 0.560 ± 0.017 |
| **PAD UFES 20** | 0.575 ± 0.022 | **0.622 ± 0.023** | 0.622 ± 0.02 | **0.631 ± 0.014** | 0.607 ± 0.022 | **0.619 ± 0.013** | **0.633 ± 0.022** | 0.584 ± 0.031 |
| **SD198** | 0.411 ± 0.041 | **0.449 ± 0.042** | 0.44 ± 0.037 | **0.473 ± 0.044** | 0.437 ± 0.037 | **0.444 ± 0.041** | **0.464 ± 0.034** | 0.403 ± 0.02 |
| **MSK** | 0.613 ± 0.015 | **0.649 ± 0.01** | 0.635 ± 0.009 | **0.652 ± 0.013** | 0.624 ± 0.013 | **0.645 ± 0.011** | **0.657 ± 0.005** | 0.631 ± 0.008 |
| **Milk10k (clinic)** | 0.499 ± 0.024 | **0.551 ± 0.034** | 0.573 ± 0.035 | **0.576 ± 0.024** | 0.522 ± 0.032 | **0.570 ± 0.032** | **0.571 ± 0.032** | 0.508 ± 0.031 |
| **Milk10k (dermo)** | 0.619 ± 0.024 | **0.664 ± 0.018** | **0.666 ± 0.013** | 0.659 ± 0.033 | 0.61 ± 0.016 | **0.648 ± 0.006** | **0.66 ± 0.012** | 0.626 ± 0.025 |
| **Text-to-image** | | | | | | | | |
| **BCN20000** | 0.580 ± 0.016 | **0.743 ± 0.011** | 0.711 ± 0.017 | **0.751 ± 0.01** | 0.51 ± 0.004 | **0.748 ± 0.004** | **0.756 ± 0.012** | 0.502 ± 0.041 |
| **derm12345** | 0.813 ± 0.007 | **0.861 ± 0.008** | 0.84 ± 0.012 | **0.863 ± 0.008** | 0.778 ± 0.02 | **0.859 ± 0.004** | **0.867 ± 0.005** | 0.759 ± 0.01 |
| **Derm7pt** | 0.448 ± 0.043 | **0.651 ± 0.02** | 0.613 ± 0.036 | **0.676 ± 0.008** | 0.371 ± 0.015 | **0.667 ± 0.006** | **0.681 ± 0.008** | 0.401 ± 0.043 |
| **DermNet** | 0.357 ± 0.033 | **0.63 ± 0.011** | 0.556 ± 0.034 | **0.645 ± 0.01** | 0.52 ± 0.022 | **0.633 ± 0.006** | **0.650 ± 0.007** | 0.311 ± 0.015 |
| **FLUO_SC** | 0.374 ± 0.025 | **0.609 ± 0.007** | 0.549 ± 0.044 | **0.618 ± 0.016** | 0.451 ± 0.015 | **0.613 ± 0.011** | **0.616 ± 0.007** | 0.289 ± 0.021 |
| **MRA_MIDAS** | 0.522 ± 0.027 | **0.688 ± 0.008** | 0.65 ± 0.024 | **0.706 ± 0.009** | 0.518 ± 0.01 | **0.700 ± 0.008** | **0.712 ± 0.012** | 0.455 ± 0.02 |
| **HAM10000** | **0.370 ± 0.035** | 0.357 ± 0.024 | **0.475 ± 0.039** | 0.419 ± 0.027 | 0.512 ± 0.047 | **0.425 ± 0.027** | **0.578 ± 0.019** | 0.603 ± 0.04 |
| **SKINL2** | 0.598 ± 0.022 | **0.766 ± 0.007** | **0.711 ± 0.024** | **0.777 ± 0.006** | 0.669 ± 0.014 | **0.764 ± 0.003** | **0.782 ± 0.005** | 0.557 ± 0.018 |
| **Fitzpatrick17k** | 0.401 ± 0.03 | **0.625 ± 0.015** | 0.619 ± 0.017 | **0.65 ± 0.013** | 0.471 ± 0.021 | **0.627 ± 0.006** | **0.640 ± 0.010** | 0.463 ± 0.024 |
| **HIBA** | 0.62 ± 0.013 | **0.753 ± 0.008** | 0.723 ± 0.014 | **0.763 ± 0.011** | 0.557 ± 0.004 | **0.761 ± 0.004** | **0.771 ± 0.011** | 0.549 ± 0.036 |
| **PAD UFES 20** | 0.551 ± 0.015 | **0.648 ± 0.013** | 0.613 ± 0.024 | **0.655 ± 0.02** | 0.433 ± 0.017 | **0.658 ± 0.013** | **0.661 ± 0.015** | 0.332 ± 0.052 |
| **SD198** | 0.471 ± 0.021 | **0.604 ± 0.014** | 0.554 ± 0.029 | **0.624 ± 0.013** | 0.509 ± 0.019 | **0.607 ± 0.011** | **0.622 ± 0.012** | 0.382 ± 0.018 |
| **MSK** | 0.607 ± 0.015 | **0.757 ± 0.012** | 0.74 ± 0.017 | **0.766 ± 0.007** | 0.567 ± 0.004 | **0.762 ± 0.005** | **0.767 ± 0.012** | 0.556 ± 0.03 |
| **Milk10k (clinic)** | 0.559 ± 0.017 | **0.692 ± 0.011** | 0.673 ± 0.023 | **0.705 ± 0.009** | 0.432 ± 0.012 | **0.700 ± 0.010** | **0.707 ± 0.015** | 0.394 ± 0.060 |
| **Milk10k (dermo)** | 0.559 ± 0.017 | **0.692 ± 0.011** | 0.673 ± 0.023 | **0.705 ± 0.009** | 0.432 ± 0.012 | **0.700 ± 0.010** | **0.707 ± 0.015** | 0.394 ± 0.060 |

TableSupplementary 10. Results on the zero-shot learning task for the MM architecture, comparing the performance reached training the model with clinical notes synthesized prompting a LLM providing lesion metadata (L; GPT, MedGemma, SkinGPT4, DermLIP) and combining the same notes with generated filling a template with metadata (LM). Performance is reported as average F1-score ± standard deviation for 8-classes setup (upper part) and 15-classes setup (lower part) across datasets; * denotes statistically significant differences between M and L (Wilcoxon test). For each couple of columns, the highest performance is marked in bold.

| **Dataset** | **L, GPT** | **LM, GPT** | **L, MedGemma** | **LM, MedGemma** | **L, SkinGPT4** | **LM, SkinGPT4** | **L, DermLIP** | **LM, DermLIP** |
| --- | --- | --- | --- | --- | --- | --- | --- | --- |
| **8-classes** | | | | | | | | |
| **BCN20000** | **0.738 ± 0.014** | 0.725 ± 0.055 | **0.718 ± 0.05** | 0.694 ± 0.062 | 0.724 ± 0.045 | **0.738 ± 0.012** | 0.691 ± 0.053 | **0.732 ± 0.06** |
| **derm12345** | 0.811 ± 0.026 | **0.83 ± 0.015** | **0.837 ± 0.016** | 0.832 ± 0.017 | **0.84 ± 0.017** | 0.839 ± 0.018 | **0.845 ± 0.017** | 0.834 ± 0.028 |
| **Derm7pt** | **0.744 ± 0.036** | 0.687 ± 0.168 | **0.664 ± 0.125** | 0.553 ± 0.191 | 0.669 ± 0.072 | **0.727 ± 0.016** | 0.67 ± 0.094 | **0.699 ± 0.073** |
| **DermNet** | **0.671 ± 0.064** | 0.652 ± 0.119 | **0.729 ± 0.103** | 0.693 ± 0.087 | **0.784 ± 0.08** | 0.738 ± 0.097 | **0.763 ± 0.077** | 0.727 ± 0.049 |
| **FLUO_SC** | 0.743 ± 0.052 | **0.778 ± 0.067** | **0.780 ± 0.061** | 0.787 ± 0.043 | **0.839 ± 0.033** | 0.77 ± 0.077 | 0.753 ± 0.082 | **0.848 ± 0.053** |
| **MRA_MIDAS** | 0.468 ± 0.026 | **0.477 ± 0.033** | **0.501 ± 0.021** | 0.492 ± 0.025 | **0.474 ± 0.017** | 0.474 ± 0.033 | **0.501 ± 0.018** | 0.487 ± 0.016 |
| **HAM10000** | 0.734 ± 0.012 | **0.736 ± 0.017** | **0.726 ± 0.018** | 0.723 ± 0.018 | 0.735 ± 0.015 | **0.735 ± 0.010** | 0.732 ± 0.015 | **0.733 ± 0.023** |
| **SKINL2** | **0.772 ± 0.025** | 0.76 ± 0.048 | **0.771 ± 0.029** | 0.730 ± 0.055 | **0.740 ± 0.051** | 0.735 ± 0.025 | 0.744 ± 0.023 | **0.767 ± 0.036** |
| **Fitzpatrick17k** | **0.516 ± 0.05** | 0.493 ± 0.056 | **0.533 ± 0.048** | 0.492 ± 0.066 | 0.525 ± 0.051 | **0.529 ± 0.016** | 0.545 ± 0.038 | **0.555 ± 0.043** |
| **HIBA** | 0.464 ± 0.073 | **0.471 ± 0.039** | **0.503 ± 0.041** | 0.489 ± 0.042 | **0.499 ± 0.065** | 0.488 ± 0.073 | 0.496 ± 0.031 | **0.505 ± 0.018** |
| **PAD UFES 20** | 0.523 ± 0.027 | **0.545 ± 0.023** | 0.542 ± 0.017 | **0.55 ± 0.017** | **0.567 ± 0.019** | 0.532 ± 0.014 | **0.570 ± 0.018** | 0.562 ± 0.028 |
| **SD198** | 0.367 ± 0.042 | **0.395 ± 0.045** | 0.353 ± 0.036 | **0.381 ± 0.058** | 0.384 ± 0.04 | **0.396 ± 0.034** | **0.429 ± 0.027** | 0.394 ± 0.035 |
| **MSK** | **0.570 ± 0.015** | 0.566 ± 0.025 | 0.55 ± 0.025 | **0.551 ± 0.033** | 0.573 ± 0.024 | **0.573 ± 0.007** | 0.569 ± 0.022 | **0.580 ± 0.024** |
| **Milk10k (clinic)** | **0.506 ± 0.027** | 0.488 ± 0.026 | **0.534 ± 0.025** | 0.512 ± 0.024 | **0.51 ± 0.026** | 0.503 ± 0.027 | 0.502 ± 0.023 | **0.510 ± 0.021** |
| **Milk10k (dermo)** | **0.642 ± 0.010** | 0.636 ± 0.012 | **0.627 ± 0.013** | 0.62 ± 0.025 | **0.613 ± 0.016** | 0.607 ± 0.008 | 0.618 ± 0.009 | **0.624 ± 0.021** |
| **15-classes** | | | | | | | | |
| **BCN20000** | **0.386 ± 0.015** | 0.351 ± 0.06 | 0.419 ± 0.107 | **0.577 ± 0.083** | 0.511 ± 0.087 | **0.714 ± 0.017** | 0.59 ± 0.009 | **0.614 ± 0.046** |
| **derm12345** | **0.45 ± 0.007** | 0.445 ± 0.012 | **0.440 ± 0.056** | 0.419 ± 0.075 | 0.156 ± 0.045 | **0.274 ± 0.007** | **0.464 ± 0.103** | 0.304 ± 0.03 |
| **Derm7pt** | **0.653 ± 0.047** | 0.479 ± 0.169 | 0.395 ± 0.151 | **0.431 ± 0.171** | **0.679 ± 0.020** | 0.679 ± 0.021 | 0.402 ± 0.116 | **0.422 ± 0.199** |
| **DermNet** | 0.57 ± 0.053 | **0.585 ± 0.071** | 0.537 ± 0.106 | **0.637 ± 0.094** | **0.739 ± 0.062** | 0.713 ± 0.100 | **0.642 ± 0.046** | 0.619 ± 0.088 |
| **FLUO_SC** | 0.436 ± 0.095 | **0.626 ± 0.178** | 0.282 ± 0.104 | **0.738 ± 0.043** | 0.650 ± 0.123 | **0.745 ± 0.072** | **0.727 ± 0.061** | 0.712 ± 0.148 |
| **MRA_MIDAS** | 0.233 ± 0.014 | **0.253 ± 0.027** | 0.256 ± 0.068 | **0.356 ± 0.046** | 0.314 ± 0.043 | **0.390 ± 0.027** | **0.391 ± 0.033** | 0.352 ± 0.031 |
| **HAM10000** | **0.156 ± 0.009** | 0.153 ± 0.019 | 0.341 ± 0.141 | **0.541 ± 0.152** | 0.279 ± 0.134 | **0.739 ± 0.007** | 0.554 ± 0.121 | **0.682 ± 0.017** |
| **SKINL2** | **0.373 ± 0.016** | 0.346 ± 0.035 | 0.426 ± 0.088 | **0.541 ± 0.091** | 0.425 ± 0.057 | **0.634 ± 0.03** | 0.525 ± 0.067 | **0.578 ± 0.009** |
| **Fitzpatrick17k** | **0.215 ± 0.019** | 0.16 ± 0.064 | **0.274 ± 0.086** | 0.229 ± 0.078 | 0.446 ± 0.013 | **0.509 ± 0.017** | 0.421 ± 0.021 | **0.446 ± 0.067** |
| **HIBA** | **0.207 ± 0.032** | 0.201 ± 0.023 | 0.208 ± 0.094 | **0.308 ± 0.078** | 0.300 ± 0.029 | **0.468 ± 0.066** | 0.301 ± 0.087 | **0.422 ± 0.019** |
| **PAD UFES 20** | 0.336 ± 0.048 | **0.425 ± 0.068** | 0.281 ± 0.053 | **0.479 ± 0.037** | 0.439 ± 0.066 | **0.518 ± 0.017** | **0.541 ± 0.019** | 0.483 ± 0.029 |
| **SD198** | 0.275 ± 0.021 | **0.294 ± 0.041** | 0.237 ± 0.033 | **0.293 ± 0.049** | 0.268 ± 0.035 | **0.306 ± 0.031** | **0.285 ± 0.018** | 0.241 ± 0.032 |
| **MSK** | **0.313 ± 0.012** | 0.295 ± 0.029 | 0.235 ± 0.029 | **0.248 ± 0.028** | **0.253 ± 0.009** | 0.219 ± 0.008 | **0.254 ± 0.055** | 0.209 ± 0.02 |
| **Milk10k (clinic)** | **0.374 ± 0.027** | 0.363 ± 0.027 | 0.300 ± 0.062 | **0.397 ± 0.033** | 0.368 ± 0.036 | **0.413 ± 0.030** | 0.422 ± 0.029 | **0.433 ± 0.03** |
| **Milk10k (dermo)** | **0.462 ± 0.009** | 0.450 ± 0.010 | 0.379 ± 0.083 | **0.487 ± 0.029** | 0.478 ± 0.032 | **0.532 ± 0.010** | 0.516 ± 0.011 | **0.529 ± 0.016** |

# Synthetic clinical notes: length and duplicate rate

This section investigates the structure of synthetic clinical notes, focusing on note length and duplicate rate.

Note length is used to identify systematic patterns associated with the prompting strategy and the underlying LLM, while the duplicate rate aims to identify the variability of the outputs.

Specifically, the duplicate rate measures the proportion of notes that are near-identical to at least one other note in the same dataset, using TF-IDF^3^ representations and cosine similarity. A note is considered a near-duplicate when its cosine similarity exceeds a threshold of 0.9. Under this definition, a duplicate rate of 0.0 indicates fully unique outputs (i.e., each image is associated with a distinct description), whereas a duplicate rate of 1.0 indicates strongly template-like generation behavior, since each clinical note has a similarity with at least another synthetic notes.

TableSupplementary10 reports the average length of clinical notes generated with GPT and MedGemma. Both models are queried with L1, L2 (both including metadata), and OL (derived from L2 without metadata). Overall, the findings show a strong interaction between prompt design and model architecture: while GPT expands with richer prompts, MedGemma appears more sensitive to token budget or internal constraints, resulting in shorter outputs under similar conditions. For GPT, L1 consistently produces shorter notes compared to L2, indicating that L1 induces more constrained or concise outputs. In contrast, L2 systematically leads to substantially longer outputs across all datasets, suggesting that the additional structure or instructions in L2 encourage elaboration. OL, which removes metadata from L2, generally produces intermediate-length outputs or with a similar length to L2, indicating that metadata contributes to verbosity but is not the only factor leading to longer outputs. MedGemma shows an opposite or less consistent behavior. In several datasets, L1 produces longer notes than L2, highlighting a different sensitivity to prompt formulation. Furthermore, L2 often leads to shorter outputs in MedGemma, suggesting that the model may compress or truncate responses when faced with more complex or metadata-rich prompts. OL tends to restore note length toward intermediate values, again indicating that the removal of metadata alleviates some of the constraints introduced by L2.

TableSupplementary11 reports the duplicate rates of clinical notes generated with GPT and MedGemm. For GPT, L1 consistently leads to extremely high duplicate rates (often above 0.9), indicating highly structured, template-like outputs. This confirms that L1 enforces a rigid generation pattern, where variability across samples is minimal. In contrast, L2 and OL substantially reduce the duplicate rate, often by a large margin, demonstrating that these prompts guarantee a larger vocabulary and structural diversity. The similarity between L2 and OL further suggests that metadata inclusion is not the primary factor driving diversity; rather, it is the prompt formulation itself. Considering MedGemma, both L1 and L2 show high duplicate rates across most datasets, indicating limited variability regardless of prompt complexity. This behavior is consistent with the shorter outputs observed previously and can be attributed to constraints such as reduced token budgets or less expressive generation capacity. OL reduces the duplicate rate compared to L1 and L2, but the values remain relatively high, suggesting that MedGemma is more prone to output repetitive or template-like generation. These findings should also be interpreted considering the results presented in Section Lesion descriptions can be queried, where GPT and MedGemma show the highest accuracy in terms of skin lesion predicted as output, when queried with metadata.

TableSupplementary 11. Overview of the average length of clinical notes, synthesized with GPT and MedGemma. The Table reports the average, considering each dataset. L1 and L2 are prompts used in the L strategy, including metadata, while OL does not include any metadata.

|  | **GPT** | | | **MedGemma** | | |
| --- | --- | --- | --- | --- | --- | --- |
| **Dataset** | **L1** | **L2** | **OL** | **L1** | **L2** | **OL** |
| **BCN20000** | 57.42 | 160.71 | 105.18 | 73.46 | 50.29 | 65.48 |
| **derm12345** | 68.23 | 156.71 | 156.27 | 63.62 | 60.51 | 64.35 |
| **Derm7pt** | 46.05 | 147.04 | 100.03 | 47.32 | 33.07 | 61.62 |
| **DermNet** | 50.41 | 146.27 | 107.39 | 50.45 | 27.91 | 62.87 |
| **FLUO_SC** | 50.63 | 150.21 | 178.50 | 64.31 | 89.94 | 64.2 |
| **MRA_MIDAS** | 66.21 | 161.11 | 182.20 | 94.35 | 134.97 | 68.9 |
| **HAM10000** | 61.90 | 157.52 | 100.67 | 61.55 | 36.58 | 66.12 |
| **SKINL2** | 57.54 | 147.76 | 100.25 | 45.17 | 84.41 | 63.5 |
| **Fitzpatrick17k** | 53.29 | 154.83 | 129.18 | 71.07 | 64.39 | 63.4 |
| **HIBA** | 57.50 | 159.45 | 109.20 | 65.9 | 39.99 | 63.53 |
| **PAD_UFES_20** | 60.21 | 165.79 | 170.80 | 78.07 | 41.54 | 64.11 |
| **SD198** | 51.46 | 148.46 | 102.98 | 54.81 | 56.46 | 59.46 |
| **MSK** | 61.46 | 161.69 | 178.47 | 71.06 | 57.5 | 65.77 |
| **Milk10k_clinic** | 57.93 | 165.86 | 178.31 | 92.05 | 51.95 | 65.2 |
| **Milk10k_dermo** | 57.41 | 166.45 | 181.08 | 83.53 | 49.31 | 65.06 |

TableSupplementary 12. Overview of the duplicate rate of clinical notes, synthesized with GPT and MedGemma. The Table reports the average, considering each dataset. L1 and L2 are prompts used in the L strategy, including metadata, while OL does not include any metadata.

|  | **GPT** | | | **MedGemma** | | |
| --- | --- | --- | --- | --- | --- | --- |
| **Dataset** | **L1** | **L2** | **OL** | **L1** | **L2** | **OL** |
| **BCN20000** | 0.9875 | 0.2745 | 0.2769 | 0.9662 | 0.9696 | 0.7475 |
| **derm12345** | 0.9894 | 0.3376 | 0.3098 | 0.9672 | 0.9729 | 0.8076 |
| **Derm7pt** | 0.9012 | 0.1309 | 0.079 | 0.7309 | 0.8716 | 0.3926 |
| **DermNet** | 0.9461 | 0.156 | 0.0596 | 0.8213 | 0.9106 | 0.6567 |
| **FLUO_SC** | 0.9744 | 0.1875 | 0.0403 | 0.8906 | 0.8746 | 0.5758 |
| **MRA_MIDAS** | 0.904 | 0.108 | 0.1805 | 0.843 | 0.902 | 0.5611 |
| **HAM10000** | 0.9893 | 0.3927 | 0.3794 | 0.9685 | 0.9817 | 0.8222 |
| **SKINL2** | 0.8632 | 0.2925 | 0.0094 | 0.7028 | 0.8632 | 0.4104 |
| **Fitzpatrick17k** | 0.8853 | 0.0107 | 0.04 | 0.576 | 0.7733 | 0.3867 |
| **HIBA** | 0.9616 | 0.1374 | 0.0811 | 0.8818 | 0.935 | 0.4994 |
| **PAD_UFES_20** | 0.9743 | 0.0679 | 0.069 | 0.9356 | 0.9382 | 0.6506 |
| **SD198** | 0.8805 | 0.0708 | 0.0265 | 0.6283 | 0.7478 | 0.4071 |
| **MSK** | 0.9759 | 0.1548 | 0.3949 | 0.9396 | 0.956 | 0.7526 |
| **Milk10k_clinic** | 0.9776 | 0.1018 | 0.1795 | 0.9323 | 0.958 | 0.7695 |
| **Milk10k_dermo** | 0.9743 | 0.1003 | 0.2721 | 0.9548 | 0.9519 | 0.7226 |

TableSupplementary12 reports the average length of clinical notes generated with SkinGPT4 and DermLIP. As in the previous cases, both models are queried with L1, L2 (both including metadata), and OL (derived from L2 without metadata). However, those prompts are not efficient when used on the two LLMs, therefore specific prompts (reported in Clinical note generation structure Section) are used in this paper: L(P), including metadata in the prompt; OL(P), without including metadata in the prompt. A consistent reduction in output length is observed compared to GPT and MedGemma, confirming the impact of architectural differences on generation behavior. Across both models, the absence of metadata (OL and OL(P)) leads to a substantial drop in note length, a trend that is stable across datasets. This indicates that metadata plays a central role in sustaining descriptive richness in these two LLMs. Additionally, model-specific prompts (L(P), OL(P)) produce shorter and more controlled outputs, suggesting that prompt specialization aligns better with the internal generation priors of these networks.

TableSupplementary13 reports the duplicate rate of clinical notes generated with SkinGPT4 and DermLIP. As in the previous cases, both models are queried with L1, L2 (both including metadata), and OL (derived from L2 without metadata). However, those prompts are not efficient when used on the two LLMs, therefore specific prompts (reported in Clinical note generation structure Section) are used: L(P), including metadata in the prompt; OL(P), without including metadata in the prompt. For generic prompts (L1, L2), SkinGPT4 achieves very low duplicate rates, indicating high diversity, while DermLIP shows significantly higher duplication, reflecting more rigid generation. However, when metadata is removed (OL), duplicate rates increase sharply for both models, often approaching extreme values. This suggests that, in the absence of contextual grounding (i.e. metadata added to the prompt), both models collapse to repetitive output patterns. With model-specific prompts, duplicate rates increase further, particularly for DermLIP, indicating that while these prompts lead to concise outputs, they also reduce variability and collapse to template-like responses. Considering that these two LLMs are more prone to hallucinate, reporting the wrong skin lesion, two distinct patterns can be identified across prompting strategies. When the duplicate rate is low, outputs are diverse but often include hallucinations that vary across samples (e.g., inferred attributes such as nationality, ethnicity, or age). Conversely, when the duplicate rate is high, outputs become highly repetitive and often collapse to generic or non-informative responses (e.g., “I am ready to review the image”). This trade-off highlights a fundamental limitation: increasing diversity does not necessarily improve correctness, while enforcing structure reduces hallucination variability but at the cost of informativeness. Notably, model-specific prompts mitigate this effect by producing concise and more controlled outputs, although at the expense of diversity.

TableSupplementary 13. Overview of the average length of clinical notes, synthesized with SkinGPT4 and DermLIP. The Table reports the average, considering each dataset. L1 and L2 are prompts used in the L strategy, including metadata, while OL does not include any metadata. Considering these two models, specific prompts designed with the architecture are used in the paper: L(P), including metadata; OL(P), without metadata.

|  | **SkinGPT4** | | | | | **DermLIP** | | | | |
| --- | --- | --- | --- | --- | --- | --- | --- | --- | --- | --- |
| **Dataset** | **L1** | **L2** | **OL** | **L (P)** | **OL (P)** | **L1** | **L2** | **OL** | **L (P)** | **OL (P)** |
| **BCN20000** | 86.96 | 63.48 | 11.38 | 27.23 | 19.09 | 33.44 | 29.57 | 23.77 | 20.52 | 10.46 |
| **derm12345** | 87.23 | 64.5 | 10.22 | 33.75 | 23.88 | 32.23 | 30.51 | 23.02 | 21.93 | 10.01 |
| **Derm7pt** | 84.48 | 68.14 | 10.3 | 32.02 | 31.56 | 27.65 | 29.09 | 21.12 | 22.85 | 9.59 |
| **DermNet** | 87.5 | 62.34 | 10.45 | 24.32 | 25.36 | 38.69 | 47.5 | 22.21 | 26.41 | 11.18 |
| **FLUO_SC** | 82.15 | 59.55 | 10.23 | 31.77 | 33.49 | 31.5 | 31.8 | 24.73 | 19.07 | 10.64 |
| **MRA_MIDAS** | 76.59 | 62.63 | 13.23 | 33.68 | 19.96 | 34.14 | 36.06 | 23.57 | 21.49 | 12.93 |
| **HAM10000** | 87.12 | 63 | 10.26 | 28.96 | 29.56 | 30.12 | 28.44 | 21.83 | 20.04 | 9.56 |
| **SKINL2** | 83 | 64.77 | 10.41 | 27.1 | 19.39 | 33.64 | 35.77 | 23.38 | 20.03 | 11.91 |
| **Fitzpatrick17k** | 85.66 | 60.84 | 8.51 | 14.45 | 26.89 | 35.26 | 44.42 | 25.67 | 29.24 | 12.71 |
| **HIBA** | 88.42 | 65.58 | 12.21 | 38.11 | 30.63 | 33.91 | 38.89 | 27.09 | 23.62 | 10.33 |
| **PAD_UFES_20** | 86.62 | 58.84 | 10.56 | 31.98 | 30.66 | 32.17 | 31.12 | 26.44 | 19.96 | 10.41 |
| **SD198** | 83.13 | 64.47 | 9.65 | 14.67 | 36.9 | 34.33 | 40.02 | 25.73 | 17.81 | 10.06 |
| **MSK** | 87.65 | 65.56 | 11.56 | 34.55 | 24.88 | 31.1 | 28.63 | 23.42 | 22.09 | 9.58 |
| **Milk10k_clinic** | 86.15 | 56.77 | 11.5 | 41.09 | 53.07 | 32.26 | 33.19 | 28.19 | 22.14 | 9.74 |
| **Milk10k_dermo** | 90.08 | 65.29 | 12.12 | 42.73 | 25.09 | 36.87 | 30.12 | 26.54 | 22.88 | 9.89 |

TableSupplementary 14. Overview of the duplicate rate of clinical notes, synthesized with SkinGPT4 and DermLIP. The Table reports the average, considering each dataset. L1 and L2 are prompts used in the L strategy, including metadata, while OL does not include any metadata. Considering these two models, specific prompts designed with the architecture are used in the paper: L(P), including metadata; OL(P), without metadata.

|  | **SkinGPT4** | | | | | **DermLIP** | | | | |
| --- | --- | --- | --- | --- | --- | --- | --- | --- | --- | --- |
| **Dataset** | **L1** | **L2** | **OL** | **L (P)** | **OL (P)** | **L1** | **L2** | **OL** | **L (P)** | **OL (P)** |
| **BCN20000** | 0.0207 | 0.0264 | 0.9743 | 0.761 | 0.9084 | 0.8487 | 0.9215 | 0.9463 | 0.9876 | 0.9927 |
| **derm12345** | 0.0423 | 0.0369 | 0.8574 | 0.6004 | 0.8606 | 0.8726 | 0.9331 | 0.9451 | 0.9905 | 0.9913 |
| **Derm7pt** | 0 | 0 | 0.9309 | 0.5821 | 0.7037 | 0.4765 | 0.6642 | 0.7975 | 0.8716 | 0.9506 |
| **DermNet** | 0.0142 | 0.0681 | 0.9404 | 0.7952 | 0.8383 | 0.7333 | 0.7121 | 0.7915 | 0.9489 | 0.9631 |
| **FLUO_SC** | 0.0154 | 0.0377 | 0.952 | 0.6878 | 0.7274 | 0.8695 | 0.9437 | 0.8125 | 0.9917 | 0.9898 |
| **MRA_MIDAS** | 0.0015 | 0.0031 | 0.9121 | 0.5838 | 0.8476 | 0.5607 | 0.6479 | 0.7536 | 0.9244 | 0.9653 |
| **HAM10000** | 0.0635 | 0.062 | 0.9497 | 0.7511 | 0.8279 | 0.898 | 0.9551 | 0.961 | 0.9927 | 0.9951 |
| **SKINL2** | 0 | 0.0094 | 0.8915 | 0.4857 | 0.7642 | 0.3491 | 0.4717 | 0.467 | 0.783 | 0.8821 |
| **Fitzpatrick17k** | 0 | 0.0053 | 0.92 | 0.728 | 0.72 | 0.7253 | 0.5973 | 0.648 | 0.88 | 0.9387 |
| **HIBA** | 0.0037 | 0.005 | 0.9108 | 0.5267 | 0.7203 | 0.6609 | 0.6244 | 0.7593 | 0.9616 | 0.974 |
| **PAD_UFES_20** | 0.0039 | 0.0104 | 0.9595 | 0.6397 | 0.7772 | 0.903 | 0.9682 | 0.8312 | 0.9869 | 0.9852 |
| **SD198** | 0 | 0 | 0.8673 | 0.7478 | 0.6239 | 0.5929 | 0.6195 | 0.6239 | 0.9469 | 0.8982 |
| **MSK** | 0.0091 | 0.0154 | 0.9208 | 0.6531 | 0.8393 | 0.7911 | 0.9127 | 0.9273 | 0.9805 | 0.9935 |
| **Milk10k_clinic** | 0.0165 | 0.0753 | 0.9784 | 0.6615 | 0.6157 | 0.9122 | 0.9397 | 0.8918 | 0.9897 | 0.9909 |
| **Milk10k_dermo** | 0.0047 | 0.0119 | 0.9344 | 0.647 | 0.8295 | 0.8141 | 0.9289 | 0.9192 | 0.986 | 0.9918 |

# OL strategy: patterns of inaccuracy

This section analyzes the content of the skin lesion included into the clinical notes generated with OL strategy, considering the four largest datasets: BCN20000, Derm12345, HAM10000, Milk10k.

For each LLM adopted into the paper, the confusion matrix for the four datasets is reported: FigureSupplementary1 includes the clinical notes generated with GPT, FigureSupplementary2 includes the clinical notes generated with MedGemma, FigureSupplementary3 includes the clinical notes generated with SkinGPT4, FigureSupplementary4 includes the clinical notes generated with DermLIP.

FigureSupplementary 1. Overview of the skin lesion accuracy considering the clinical notes synthesized with GPT.

FigureSupplementary 2. Overview of the skin lesion accuracy considering the clinical notes synthesized with MedGemma.

FigureSupplementary 3. Overview of the skin lesion accuracy considering the clinical notes synthesized with SkinGPT4.

FigureSupplementary 4. Overview of the skin lesion accuracy considering the clinical notes synthesized with DermLIP.

# Bibliography

1.	Chen, T., Kornblith, S., Norouzi, M. & Hinton, G. A Simple Framework for Contrastive Learning of Visual Representations. Preprint at https://doi.org/10.48550/arXiv.2002.05709 (2020).

2.	Oord, A. van den, Li, Y. & Vinyals, O. Representation Learning with Contrastive Predictive Coding. Preprint at https://doi.org/10.48550/arXiv.1807.03748 (2019).

3.	Christian, H., Agus, M. P. & Suhartono, D. Single Document Automatic Text Summarization using Term Frequency-Inverse Document Frequency (TF-IDF). *ComTech* **7**, 285–294 (2016).
